# The Thermo-Oxidative Degradation of Polyurethane Open-Cell Soft Foam Investigated Through Gas Chromatography and Mass Spectrometry of Volatile Organic Compounds

**DOI:** 10.3390/polym16233342

**Published:** 2024-11-28

**Authors:** Christian Stefan Sandten, Martin Kreyenschmidt, Rolf Albach, Ursula E. A. Fittschen

**Affiliations:** 1 Department of Chemical Engineering, University of Applied Sciences Muenster, 48565 Steinfurt, Germany; martin.kreyenschmidt@fh-muenster.de; 2 Covestro Deutschland AG, 51373 Leverkusen, Germany; rolf.albach@covestro.com; 3 Institute of Inorganic and Analytic Chemistry, Clausthal University of Technology, 38678 Clausthal-Zellerfeld, Germany; ursula.fittschen@tu-clausthal.de

**Keywords:** thermal oxidation, emission chamber, degradation, segregated block copolymer, polyurethane soft foam, VOC, TD-GC-MS, PUR

## Abstract

Polyurethane (PUR) soft foams release malodorous and potentially toxic compounds when exposed to oxidative conditions. Current chamber test methods cannot distinguish between pre-existing volatiles and those formed during oxidation, nor can they assess the formation rates of oxidation products. We subjected PUR soft foam to oxidative treatment in a continuous air flow at 120 °C. Emissions were convectively transferred from the foam to an exhaust port and analyzed using a thermodesorption–gas chromatography–mass spectrometry (TD-GC-MS) system, with external calibration employed for the quantification of selected analytes. The study identified hydroperoxide formation and degradation as key mechanisms in the breakdown of the polyether soft segments. This process predominantly produces volatiles, such as carboxylic acids, formates, acetates, alpha-hydroxy-ketones, (unsaturated) aldehydes, substituted dioxolanes and dioxanes, glycols, and allyl ethers. Volatiles associated with the degradation of the hard segments include aniline, benzoxazole, 2-methylbenzoxazole, and benzaldehyde. This experimental setup enables reproducible qualitative and quantitative analysis of volatiles formed during the oxidative degradation of PUR soft foams, providing new insights into the segment-dependent chemical pathways of the polymer’s molecular breakdown.

## 1. Introduction

Open-cell flexible polyurethane (PUR) foams are indispensable in numerous applications due to their customizable properties, particularly their ability to offer comfort and low weight. These characteristics make them ideal for applications in automotives, furniture, and mattresses. The polymer matrix of flexible PUR foams consists of approximately 60–80 wt% polyether or polyester soft segments and 20–40 wt% polyurea hard segments, linked by urethane bonds. The polyether polyols are predominantly synthesized as statistic or block copolymers using the monomers ethylene oxide (EO) and propylene oxide (PO), forming polyethylene oxide (PEO) and polypropylene oxide (PPO). Common isocyanates are methylene diphenyl diisocyanate derived oligomer blends (MDIs) or toluene di-isocyanate isomer blends (TDIs) [1,2,3,4,5,6,7,8].

Volatile organic compounds (VOCs) are a known issue in flexible polyurethane foams. They stem from the thermal–oxidative degradation of the polymer, impure reactants, or byproducts. These VOCs include concerning compounds like formaldehyde, acetaldehyde, and acrolein [9]. Given the prevalence of VOCs in PUR foam emissions, extensive research has been conducted on the oxidative stability of the precursors forming the soft segments. Studies show that PEO is more resistant to oxidation than PPO, although both degrade under heat and oxidative conditions, leading to the formation of harmful compounds [10].

PEO–PPO block copolymers have been observed to release formaldehyde and acetaldehyde. The release of formaldehyde is attributed to both the PEO and PPO segments, while acetaldehyde is associated exclusively with the PPO segment [11]. Under an oxidizing atmosphere, PEO degrades to form water, CO_2_, formaldehyde, acetaldehyde, and methylformate. Acetaldehyde is only produced in trace amounts. PPO, on the other hand, forms the same degradation products, along with methylacetate and acetone, but it produces acetaldehyde in larger quantities [12]. Numerous studies demonstrate that polyethers, when oxidized, form formaldehyde, acetaldehyde, and potentially volatile alcohols, acetates, formates, and acetals [13,14,15,16]. The degradation of polyether polyols is mediated by hydroperoxide formation, which occurs randomly along the polymer backbone [13,17,18]. Hydroperoxides in polyether alcohols show temperature- and concentration-dependent formation and degradation rates. At 100 °C, a PEO–PPO copolymer accumulates hydroperoxide groups for approximately 100 min, after which the concentration drops to one-fifth of the maximum and then stabilizes [19]. Hydroxy groups inhibit the thermo-oxidative breakdown of polyethers [10,20,21]. Various degradation pathways for hydroperoxides in both PEO and PPO have been described in the literature [22,23], as has the oxidative behavior of allyl-terminated polyether monools, formed as side products in the synthesis of PPO for polyurethane production [20,24].

Research on the oxidative stability of isocyanates and their carbamates is limited. For isocyanates, studies have examined the formation of hydroperoxide groups on the methylene bridge between the two aromatic rings of unreacted MDI, as well as after urethane formation [25,26]. Investigations into the oxidative stability of polyurethanes have focused on the relationship between hydroperoxide concentrations in polyethers used for foam synthesis and the yellowing behavior of the resulting polyurethanes [27], the mechanisms of soft segment chain degradation as a function of the temperature [28], and the influence of antioxidant concentrations on VOC formation and emission [29].

VOC research in polyurethanes typically focuses on emissions resulting from the initial loading of a product. Studies on VOC emissions due to thermo-oxidative degradation remain relatively limited. Investigations have been conducted on emissions from foam mattresses [28] and the formation of odorous substances after both natural and artificial aging [29]. Further research has examined acetaldehyde emissions as a function of the molecular structure of polyether polyols used in PUR synthesis [30], as well as the formation of oxidatively generated VOCs during the aging of rigid polyurethane foam in an oxidizing atmosphere at 150 °C [31].

Generating reliable and reproducible VOC analysis results is particularly challenging when investigating cellular materials, such as flexible PUR foams. The material’s open-cell content, tortuosity, surface area, adsorptive interaction, and thermal self-insulation have varying but substantial impacts on the results of an emission measurement. Furthermore, the initial loading, the formation rate, and the sample age are parameters with an unknown relevance to the test results [32,33]. On the side of the analytical methods, the temperature of the measurement, the sampling gas flow rate, the humidity, and the test chamber volume are additional parameters with unknown impacts [34]. Therefore, variations in any of these parameters have an unknown influence on the results attained using analytical VOC determination methods [35,36,37,38,39,40]. A review of sampling volatile organic compound emissions from consumer products is available, and it lists a variety of different methods [41]. Presently, there is no reliable analytical method available to selectively investigate the multitude of thermo-oxidatively formed volatile compounds from polyurethane flexible foams. None of these methods provide any long-term emission information about the oxidation of the polyurethane matrix.

Given the growing importance of reducing VOC emissions for consumer protection, especially in sectors like automotive production and mattresses, there is a clear need for better analytical methods. The existing techniques do not adequately account for the oxidative degradation of polyurethane foams over time, nor are they reliable in quantifying the variety of VOCs formed.

In response to this, we aimed to develop a novel analytical method for the qualitative and quantitative VOC analysis of open-cell polyurethane foams by addressing the limitations of current techniques. In a previous study, we demonstrated a reproducible method for analyzing volatile oxidation products specifically focusing on low molecular weight aldehydes [9]. Building on this work, we now present a broader approach using thermodesorption–gas chromatography–mass spectrometry (TD-GC-MS), which provides a more comprehensive analyte overview, including VOCs and semi-volatile organic compounds. Our investigations were conducted at 120 °C to accelerate the polyurethane autoxidation process, thus allowing the system to reach a quasi-steady state for improved reproducibility. This elevated temperature not only increased the formation rate of oxidation products but also enhanced their desorption and diffusion from the foam matrix. Consequently, this higher temperature allowed us to generate a more extensive dataset compared to the lower temperatures typically used in oxidation studies. Importantly, the experimental setup can be easily adjusted to study oxidative processes at different temperatures, both lower and higher.

This method allows us to overcome the limitations of previous VOC measurements by offering a reproducible, long-term analysis of thermo-oxidative emissions. Our approach eliminates the variability caused by initial sample loading or atmospheric adsorption and enables a quantitative assessment of the degradation products formed within the polymer structure. These data are invaluable for developing computational models of polymer degradation and VOC emission, offering manufacturers a reliable tool for targeted research and ensuring both consumer protection and product stability.

## 2. Materials and Methods

### 2.1. Materials

#### 2.1.1. Chemical Compounds

Formic Acid (Cat: 8.22254.1001; Lot: S6088454 412), acrolein 90% (Cat: 110221), propylene glycol diacetate (Cat: 528072-1L; Batch: 05028CJ), and p-toluidine (Cat: 8.05315.0250; Lot: S8083915 211) were sourced from Sigma-Aldrich, Steinheim, Germany. Paraformaldehyde 97% was sourced from Alfa Aesar, Karlsruhe, Germany (Cat: A11313; Lot: 10199884). Acetic acid (glacial) 100% (Cat: 1.00063.2511; Lot: K49116063 720), Butyric Aldehyde (Cat: 8.01555.0100; Lot: S7554365-040), Aniline pro analysi (Cat: 1.01261.0250; Lot: K38994761 835), N,N-Dimethylformamide SupraSolv (Cat: 1.10983.2500; Lot: I466983 903), and acetaldehyde (Cat: 8.00004.0500; Lot: S4983404 805) were sourced from Merck KGaA, Darmstadt, Germany. Isoquinoline (Cat: 8.02406.0100; Lot: S3972306 511), acridine (Cat: 8.21655.0005; Lot: S4548655 839), and Triethylene diamine (Cat: 8.03456.0250; Lot: S27812 008) were sourced from Merck Schuchardt OHG, Hohenbrunn, Germany. Propionic aldehyde (Cat: 427211000; Lot: A0458037), hydroxyacetone (Cat: L15008; Lot: 10229401), benzaldehyde (Cat: A10348.30; Lot: 10243680), 2-Methylbenzoxazole (Cat: A13198; Lot: 10228765), and 2,6-Di-tert-butyl-p-benzoquinone (oxidized BHT) (Cat: A13091.06; Lot: 10196260) were sourced from Thermo Scientific, Darmstadt, Germany. Acetone Chromasolv (Cat: 34850-2.5L; Lot: L2250S) was sourced from Honeywell Research Chemicals, Seelze, Germany. Butanone (Cat: 8403.3; Lot: 029275171) was sourced from Carl Roth GmbH, Karlsruhe, Germany. Propane-1,2-diol (Cat: P/7440/17; Batch: 1001976) was sourced from Fisher Scientific Darmstadt, Germany. Diethylene glycol (Cat: 0194-500ML; Lot: 19C0456800) was sourced from VWR Chemicals, LLC, Darmstadt, Germany. Dipropylene glycol (mixture of isomers) (Cat: 62581; Lot: 21890) was sourced from Riedel-de Haen AG, Seelze, Germany. o-Toluidine purum p.a. (Cat: 89610; Lot: 1313539 20208237) was sourced from Fluka, Darmstadt, Germany. 2,2,6,6-Tetramethylpiperidinyloxyl (Cat: OR59926; Lot: AS475157) and 1,3-Benzoxazole (Cat: OR13697; Lot: AS403722) were sourced from Apollo Scientific, Stockport, UK.

#### 2.1.2. Polyethers

The polyol was provided by the Covestro Deutschland AG, Leverkusen, Germany. It was a block copolymer initiated with glycerol and propylene glycol, featuring a PPO–PEO block structure.

The polyether polyols are produced batchwise through anionic ring-opening polymerization and have the following structure:

(C_3_H_5_O_3_)-((O-CH_2_CH(CH_3_)-)_b_(O-CH_2_-CH_2_)_a_-OH)_3_

These polyether polyols contain a certain amount of allyl terminated polymer chains, as propylene oxide can rearrange to allyl alcohol during anionic ring-opening polymerization. Allyl alcohol can then act as an additional starter for the polymerization itself.

CH_3_-C_2_H_3_O → CH_2_=CH-CH_2_OH → CH_2_=CH-CH_2_-(O-CH_2_CH(CH_3_)-)_b_(O-CH_2_-CH_2_)_a_-OH

#### 2.1.3. Polyisocyanate

The isocyanate component consisted of a blend of monomeric isomers of MDI and oligomeric homologs, with an isocyanate content of 33.1% blended by Covestro Deutschland AG, Leverkusen, Germany.

The isocyanate employed had the following structure:

O=C=N-Ar-(CH_2_-Ar-NCO)_c_-CH_2_-Ar-N=C=O

**Figure 1:**
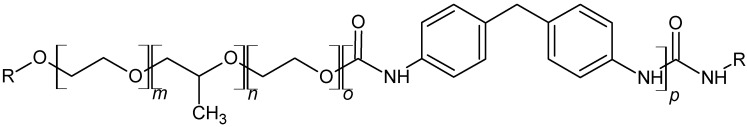
Idealized molecular structure of examined polyurethane.

The polymer resulting from the polyaddition of these compounds and water is a segmented polyurethane (Figure 1).

At index 90, the recipe ideally leads to 223 mmol (approx. 0.44 mmol/g foam) of excess of isocyanate-reactive groups. These are for kinetic reasons dominantly secondary hydroxyl groups. The reaction of water and isocyanate ideally leads to 2033 mmol of urea (Table 1). The polymerization reaction eventually becomes diffusion-controlled due to the reaction mixture’s increasing viscosity during polymer formation. Therefore, some of the isocyanate will not react to urea or urethane but to allophanate and biuret. These groups have a lower thermal stability than urea and urethane groups and reversibly open in the range of 120 °C.

The composition of the soft segment is comprised of glycerol:propylene oxide:ethylene oxide 1:50:8 by weight and a molar ratio of 0.06:8.7:1.8. This leads to 10.9 mmol of ether bonds per g of foam (8.95 mmol of them based on propylene oxide) with 41 mol-% of the bond-adjacent carbons being secondary.

The chemical crosslink density in the polyether backbone is 0.15 mol/kg foam. The stabilizer (1%) contains an alkylene oxide segment, but the composition is not disclosed. The foam sample consists of 62 weight-% polyalkylen oxide soft segments.

#### 2.1.4. Synthesis of Qualitative and Quantitative References

To generate qualitative comparisons through mass spectra and retention times, several possible analytes were synthesized and characterized. The synthetic procedures are outlined in Supplemental Material I.

##### Dioxolanes

All six dioxolanes resulting from ethylene glycol or propylene glycol with formaldehyde, acetaldehyde, and propionic aldehyde were synthesized through condensation reactions. An excess of the glycol was reacted with the aldehyde under sulfuric acid catalysis. The mixtures were stirred at room temperature overnight, and the reaction product was removed from the reaction mixture through distillation after neutralization with sodium bicarbonate.

##### Glycol Esters

The synthesis and purification of glycol esters carrying one or two of the same parent acid ester groups presented notable challenges due to the minor differences in boiling points and chromatographic behaviors among the parent glycol, mono-ester, and di-ester.

To solve this challenge, the mono- and di-formates of the glycols were synthesized in situ within the calibration solutions. Calibration solutions for mono-esters were crafted by employing the glycols as solvents and adding the targeted ester’s equivalent molar amount of acid. For diester calibration, acids were utilized as solvents along with the addition of the targeted diester’s equivalent molar amount of glycol. The completion of acid or glycol conversion was confirmed through GC-MS measurements. The generated compounds were used for qualification and, for some compounds, for calibration (Table 2).

The positional isomers of the monoesters of propylene glycol (1,2-propanediol-1-formate, 1,2-propanediol-2-formate, 1,2-propanediol-1-acetate, 1,2-propanediol-2-acetate) were identified by their mass spectra. The detector response of the respective formates and acetates was assumed to be identical, enabling the assignment of mass fractions corresponding to peak areas and facilitating emission quantification.

#### 2.1.5. Sample Chamber Creation

**Figure 2:**
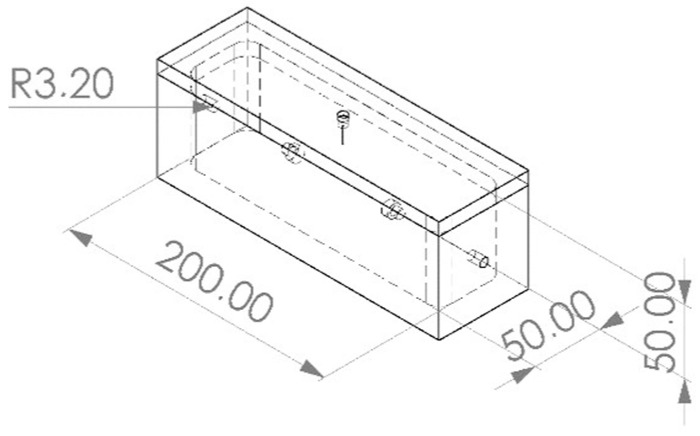
Geometry of the sampling chamber. Annotated lengths in mm.

A PTFE block measuring 70 mm by 70 mm by 220 mm underwent milling processes to carve out a cavity resembling the shape of a sarcophagus measuring 50 mm by 50 mm by 200 mm. Subsequently, a second PTFE block was milled to fabricate a fitting lid. The resulting closed sampling chamber features a cavity of approximately 500 mL accommodating foam samples measuring 50 mm by 50 mm by 200 mm (Figure 2). To facilitate sealing, a ¼ inch diameter hole was drilled into each of the two short ends of the chamber, into which sockets were inserted to allow for the insertion of sealing gaskets composed of fluoroelastomer material. A total of five chambers of this design were produced for this experiment.

#### 2.1.6. Polyurethane Foam Slab Synthesis

**Figure 3:**
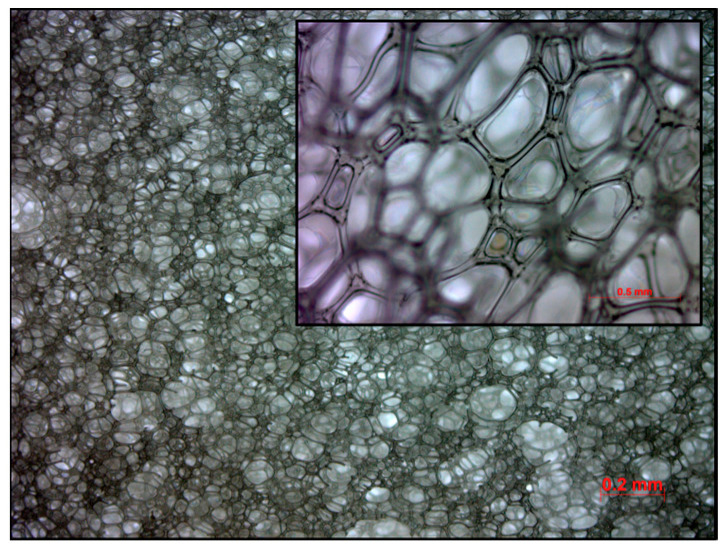
Cell structure of investigated foam sample under microscope.

A foam slab was synthesized within a preheated mold with a capacity of 16 L, maintained at a temperature of 90 °C. The mass of the raw material mixture was calculated to be 650 g, ensuring minimal overpacking of the mold. Synthesis was conducted with an index of 90 (the molar ratio of isocyanate groups to hydroxy groups multiplied by 100). The blend of polyether polyol and additives was premixed. In the following step, the isocyanate was added, and the resulting mixture was stirred at a speed of 4200 rpm with a Pendraulik LM 34 stirrer, disperlux-pendraulik, 31832 Springe, Germany, for 15 s. The cream time, when the CO_2_-generating reaction of isocyanate and water started, was approximately 25 s. After 45 min, the foam was demolded. The resulting foam possesses a density of 40.6 g/L (Figure 3). The temperature of the mold and the residence time in the mold are much higher than typical for industrial production to accommodate for lower mixing energy compared to industrial higher-energy mixing and higher foam core temperatures.

### 2.2. Sample Preparation

**Figure 4:**
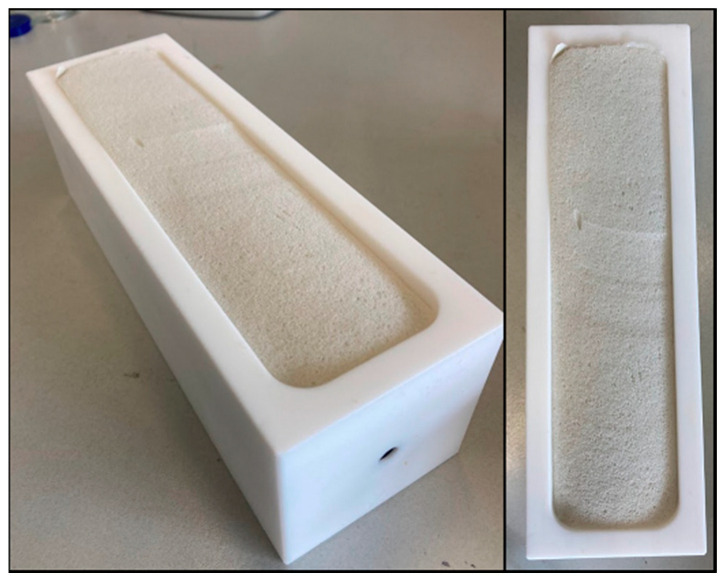
Sample foam in sampling chamber.

A ceramic knife was used to trim the outer edges of the foam slab. Following this, the slab was divided into five pieces, each taking on a roughly cuboid shape. These pieces were further refined using a standard household slicing machine to achieve the desired dimensions of 55 mm by 55 mm by 210 mm (635.25 mL) for each cuboid. These dimensions were deliberately chosen to slightly surpass the inner volume of the chamber, thus ensuring a snug fit against the chamber walls to maintain gas tightness. The cuboids were extracted from the central region of the original slab, devoid of any foam skin, resulting in sample cuboids with uniform composition. Subsequently, these cuboids were tightly fitted into Teflon PTFE chambers and sealed hermetically from the outside using conventional one-part silicone sealant and zip-ties (Figure 4).

### 2.3. Methods

#### 2.3.1. Aging Methodology

**Figure 5:**
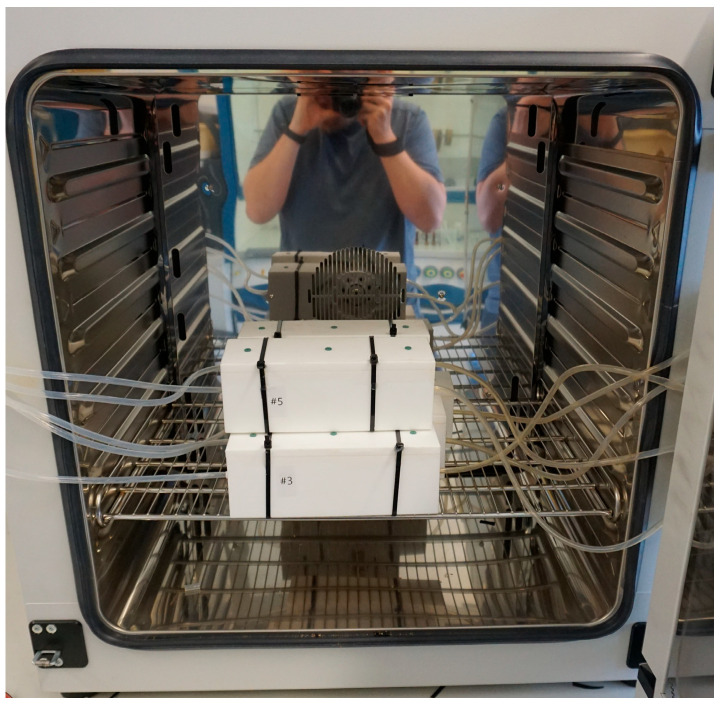
Sample chambers in laboratory oven. PTFE tubing attached to inlet (left) and outlet (right) of chambers.

**Figure 6:**
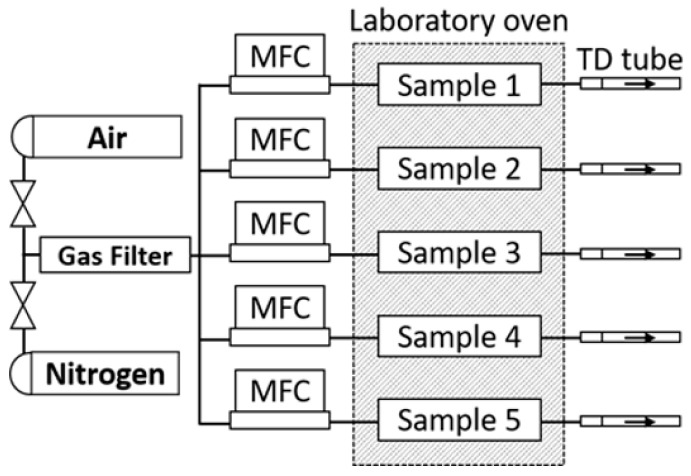
Flow chart of sampling system.

The experimental setup for investigating foam sample conditioning and aging comprises a polytetrafluoroethylene (PTFE) chamber, a gas supply line equipped with mass flow control, an oven for precise temperature regulation, a gas purification cartridge, and a sampling protocol enabling time-dependent analyses (Figure 5). PTFE was selected to minimize surface interactions between the chamber walls and the analytes. The gas supply is employed to continuously purge samples with purified pressurized air or nitrogen. This continual replacement of the atmosphere within the foam induces a shift in the adsorption equilibrium of volatile organic compounds (VOCs) towards the gaseous phase. The removal of adsorbed analytes diminishes the surface concentration, prompting an augmentation in analyte diffusion towards the polymer surface. Owing to the chamber’s design and the cellular structure of the sample, a plug flow is established, ensuring uniform gas velocity throughout the sample’s cross-section [42]. Plug flow precludes back mixing of emitted substances within the sample, thereby enhancing sample purging efficiency. The VOCs emitted from the sample are convectively transported outside of the oven, adsorbed onto thermo-desorption tubes, and subsequently analyzed via TD-GC-MS. Sampling of the emissions took place by attaching ¼” Tenax TA tube by Camsco, Houston, TX, USA to the emission testing chamber’s exhaust port. A laboratory oven, the Binder Model FD 115, Binder GmbH, 78532 Tuttlingen, Germany, was used to maintain precise temperature control throughout the experiments. Flow control was managed utilizing a Buerkert single-phase primary switched power supply, coupled with a CM22-0-10 V potentiometer by Cobi Electronic, TME Germany GmbH, Leipzig, Germany, and mass flow controllers of the type 8741, also by Buerkert, Ingelfingen, Germany. The gas supplies underwent filtration via a gas purifier cartridge, the Big Hydrocarbon Trap Model BHT-4, by Agilent, 76337 Waldbronn, Germany, thus effectively reducing hydrocarbon levels to below 15 ppb. Teflon tubing was used for supplying gas flow to the sample chambers and directing emissions from within the oven to the exterior for sampling purposes (Figure 6).

#### 2.3.2. Thermo-Oxidation at 120 °C

Before commencing the investigations, the samples underwent a seven-day equilibration period at 120 °C within a constant air stream. This temperature selection aligns with the standards outlined in VDA 278 [43], facilitating the concurrent determination of VOC and semi-volatile organic compounds.

The volume flow rate was 200 mL/min, resulting in a gas exchange rate of approximately 2.5 min^−1^. The relative humidity of the pressurized air was maintained at 8% rH at 19.7 °C, translating to 1.15 g/kg or 1.48 g/m^3^. This is equivalent to 82.4 mmol/m^3^ or 16 µmol/min or 274 nmol/s.

The temperature and the gas stream were maintained throughout all experiments.

#### 2.3.3. Reproducibility Investigations

Sampling was conducted five times on five individual samples equilibrated at 120 °C for a week for over 20 min each. The temperature of the thermodesorption tubes was monitored with an IR thermometer. The temperature at the end attached to the exhaustion port increased to 27 °C. One centimeter from the exhaust port, the temperature dropped to ambient room temperature.

#### 2.3.4. Sampling Time Variation

After the initial equilibration phase, samples were taken from one sample over different time intervals of 0.5 min, 1 min, 1.5 min, 2 min, 3 min, 5 min, 7 min, 10 min, 15 min, 25 min, and 32 min to assess the impact of their variation.

#### 2.3.5. Identification of Oxidation Products

To investigate the influence of the atmospheric oxygen concentration on the formed emissions, a mass flow controller was connected to a compressed air supply and another mass flow controller to a nitrogen supply (for the experiment at 100% O_2_, a grade 5.0 oxygen cylinder was used). The volume flow rate was maintained at 200 mL/min. The oxygen content was adjusted by the ratios of the volumetric flows of the two gases. To avoid accumulation of oxidatively formed species in the system, the experiment was conducted incrementally from 0% oxygen upwards. The emissions were investigated at oxygen concentrations of 0%, 1%, 2.5%, 4%, 7%, 20%, and 100%. The sample was flushed for 72 h with nitrogen before sampling started. A waiting period of 24 h was maintained between each sampling. Each sampling was performed in triplicate.

#### 2.3.6. Thermal Desorption Unit (TD)

The thermal desorption unit (PerkinElmer TurboMatrix 350 ATD, Rodgau, Germany) was set to −30 °C for cryo focusing with a valve temperature of 250 °C, a temperature of 280 °C for the ten-minute tube desorption, and a transfer line temperature of 200 °C. The column flow was set to 1 mL/min. The inlet and outlet splits were set so that 5% of the total sample mass was injected into the GC system.

Following the measurement, the used tubes were conditioned using a TC-20 by Markes International, Offenbach am Main, Germany. The tubes were heated to 280 °C for 2 h in a constant nitrogen flow.

#### 2.3.7. Gas Chromatography and Mass Spectrometry (GC-MS) Parameters

A GCMS-QP2020 by Shimadzu Deutschland GmbH, Duisburg, Germany, with a PerkinElmer TurboMatrix350 was employed for the analysis of the samples. A Shimadzu SH-Rtx-200MS crossbond trifluoropropyl-methylpolysiloxane GC column with an inner diameter of 0.25 mm and a length of 30 m was used for the chromatographic separation of the analytes.

The oven temperature was held at 30 °C for 5 min and then heated to 120 °C with a heat rate of 2 °C/min, followed by a heat rate of 5 °C min^−1^ to the final temperature of 240 °C. The total program time was 74 min.

The ion source temperature was set to 200 °C, and the interface temperature was held at 250 °C. The MS started at 1.7 min with a scan speed of 10,000 and an event time of 70 ms, and it scanned the mass range from 19 to 500 *m*/*z*.

Qualitative analysis of the VOC was based on reference standards or proceeded through comparison of mass spectra with NIST 05, NIST 05s, NIST08, and NIST08s databases using Shimadzu’s LabSolutions GCMS solution version 4.45.

#### 2.3.8. External Calibration Protocol for Quantification

Camsco ¼” Tenax TA tubes were spiked with the appropriate amounts of the respective analytes. Analytes were dissolved in methanol, a dilution series was created, and 5 µL of each standard was injected into the tube while a continuous flow of 200 mL/min of purified nitrogen ran through the tube. Each tube was purged of the solvent for five minutes. For aldehyde standards, pentane was used as the solvent to avoid the formation of acetals. Each calibration set consists of 7 levels with three replicate measurements. The calibrations of glycol esters were performed with 7 levels with only one measurement per concentration level.

## 3. Results

### 3.1. Qualitative Results

#### 3.1.1. Most Prominent Peaks

**Figure 7:**
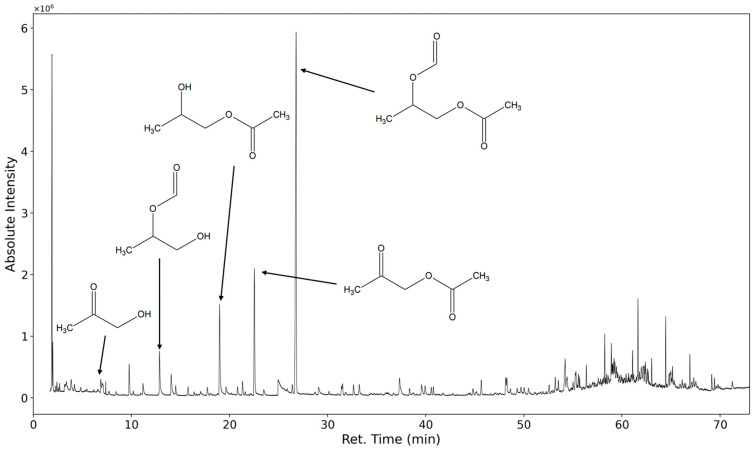
TIC of TD-GC-MS chromatogram of polyurethane sample emissions thermo-oxidized at 120 °C for one week.

In Figure 7 the chromatogram of the emissions collected for 20 min of a polyurethane flexible foam thermo-oxidized for one week at 120 °C are shown. In total, several hundred distinct analytes are observed. The identification of these substances is challenging because the mass spectra of higher oligomers and their derivatives strongly resemble the spectra of the lower molecular weight homologs.

The five marked peaks at 27 min, 22.5 min, 19 min, 12,8 min, and 8 min are caused by 1,2-propanediol-1-acetate-2-formate, acetyloxyacetone, 1,2-propanediol-1-acetate, 1,2-propanediol-2-formate, and hydroxyacetone.

#### 3.1.2. Degradation Products of the Soft Segment (EO Phase)

**Figure 8:**
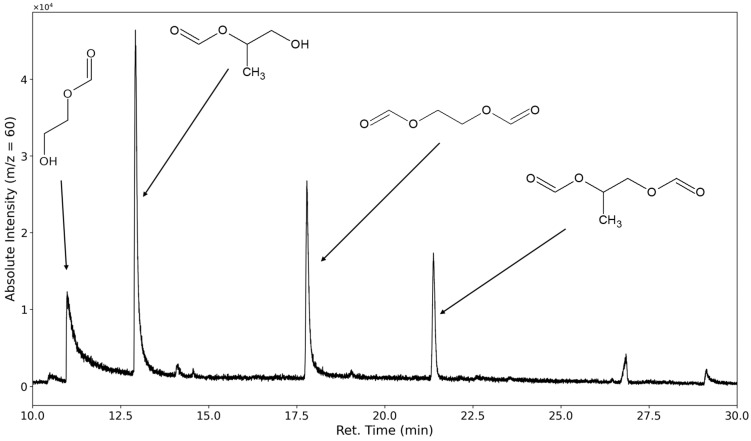
Selected ion chromatogram of the mass-to-charge ratio 60. 1,2-ethanediol-1-formate, 1,2-ethanediol diformate, 1,2-propanediol-2-formate, and 1,2-propanediol diformate are marked.

The decomposition of polyethylene oxide through hydroperoxide formation and degradation should lead to the formation of terminal formates and aldehydes. The most selective ion to investigate for PEO degradation was *m*/*z* = 60, as the formates would form a fragment with the molecular formula of C_2_H_4_O_2_ after ionization on the carbonyl oxygen followed by a McLafferty rearrangement (see Figure 8).

The mono- and diformate of 1,2-ethanediol could be identified. Peaks corresponding to the formates of the higher molecular ethylene glycols, such as diethylene glycol, could only be found in limited cases under severe oxidative stress (see Section 3.3). The only aldehyde found that forms from the degradation of the polyethylene oxide is the dialdehyde derived from diethylene glycol. No products were observed that contained terminal acid groups; however, this would only be observed if initially formed aldehyde compounds were further oxidized or if substances containing ester groups were formed in the polyethylene oxide chain and subsequently hydrolyzed.

#### 3.1.3. Degradation Products of the Soft Segment (PO Phase)

**Figure 9:**
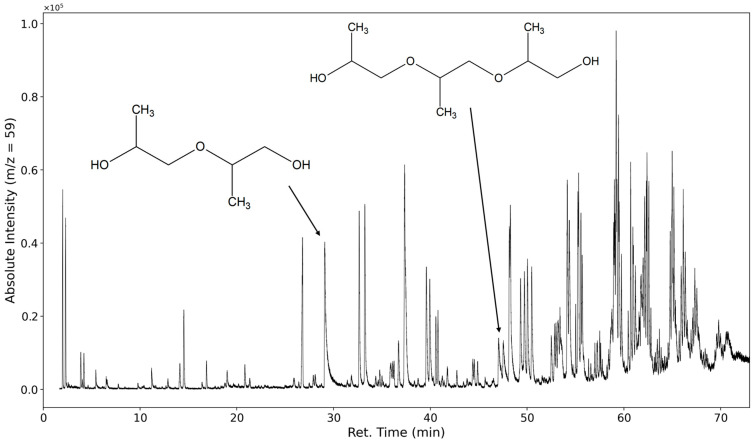
Selected ion chromatogram of the mass-to-charge ratio 59. Dipropylene glycol and tripropylene glycol are marked.

In Figure 9, the selected ion chromatogram of *m*/*z* = 59 is shown. This ion is a strong indicator of a fragmentation product of the polypropylene oxide segment of the polyether. It is formed from the cleavage of the C-O bond of a terminal repeating unit (C_3_H_7_O or HO-CH-(CH_3_)-CH_2_-).

At 30 min, dipropylene glycol is detected, and at 47 min, tripropylene glycol is detected. Several formates and acetates of higher oligomeric propylene glycols are identified as well; however, we were unable to baseline separate them due to the abundance of highly similar compounds.

**Figure 10:**
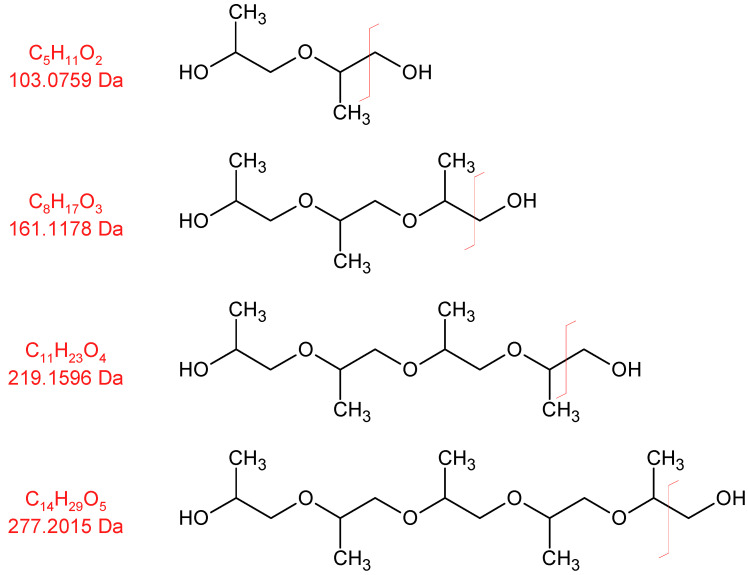
Electron impact ionization fragments formed by oligomeric propylene glycols. Red line indicates bond cleaveage leading to the observed ion fragment.

**Figure 11:**
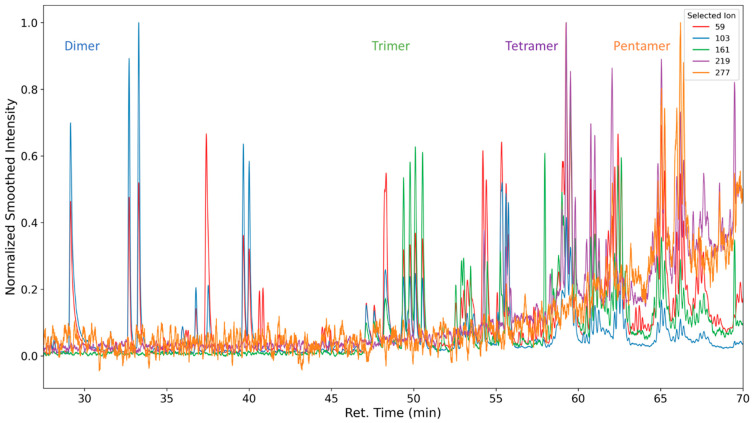
Selected ion chromatograms of propylene glycol oligomers emitted from thermo-oxidized polyurethane foams. Intensity values were smoothed with a rolling average with a window size of 75. Intensity values for selected ion 219 for the retention times 62.95 to 63.15 were removed from the dataset due to an analyte interfering with normalization that was not propylene-glycol-related.

Oligomeric propylene glycols of molecular weights greater than 277 g/mol (C_14_H_29_O_5_) are detected. Polypropylene glycol fragments with a length of at least five repeating units were identified (Figure 10 and Figure 11). These high-molecular compounds are only observed under oxidizing conditions.

**Figure 12:**
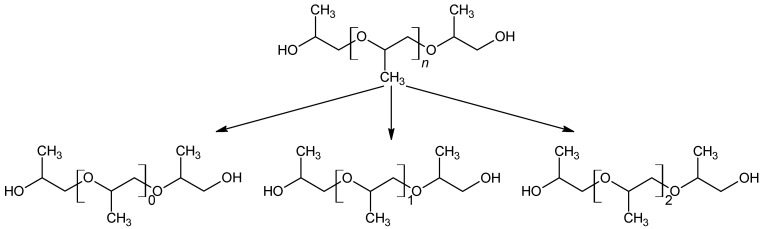
Selective formation of a selection of defined configurational isomers from polypropylene oxide.

As the formation takes place from the poly propylene oxide segment of the polyether polyol, which consists predominantly of head–tail connections of propylene oxide, only the respective positional isomers (methyl group position) are observed (see Figure 12).

Higher molecular weight products can be detected, but the chain length distribution of VOC emissions primarily consists of derivatives of the monomeric glycols. In regular non-convective sampling VOC analysis, this result could easily be explained by the limited vapor pressure of higher glycol derivatives. We assume that in this setup, the majority of emission rates are not limited by the compounds’ vapor pressure and therefore are in equilibrium with their formation rates. Consequently, a degradation pathway favoring the production of low molecular weight products seems to dominate. The ion fragment *m/z* 43 is assumed to be mostly formed by groups with the molecular formula of C_2_H_3_O, which is commonly encountered in acetates and alpha-methyl ketones.

**Figure 13:**
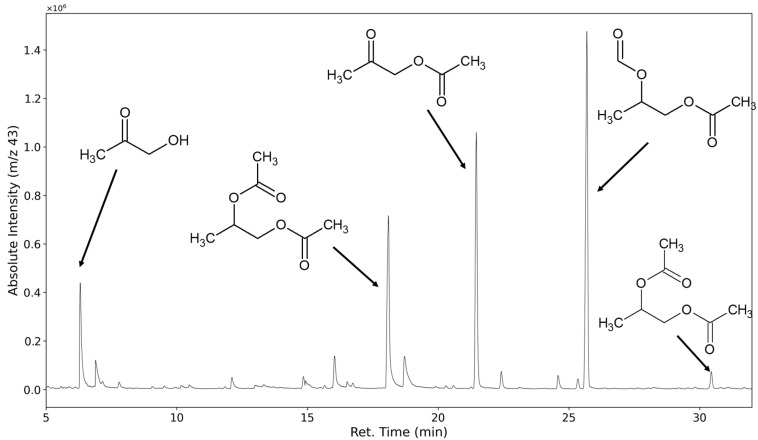
Selected ion chromatogram (*m/z* = 43) of polyurethane sample thermo-oxidized at 120 °C for one week.

In Figure 13, the selected ion chromatogram of *m/z* = 43 shows hydroxyacetone at 6.73 min, the monoacetates of propylene glycol at 18.9 min and 19.5 min, the mixed acetate and formates of propylene glycol at 26.4 min, and its diacetate at 31.35 min.

#### 3.1.4. Degradation of the Hard Phase Segment

The volatile oxidation products of the hard segment are formed and emitted in far lower concentrations compared to the products formed by the soft segment oxidation. Under oxidizing conditions, aniline and toluidine are not detected, or they are detected only with low peak areas, which is probably due to the consecutive reactions. Therefore, measurements were conducted under inert and oxidizing atmospheres to present the versatility of the presented method.

**Figure 14:**
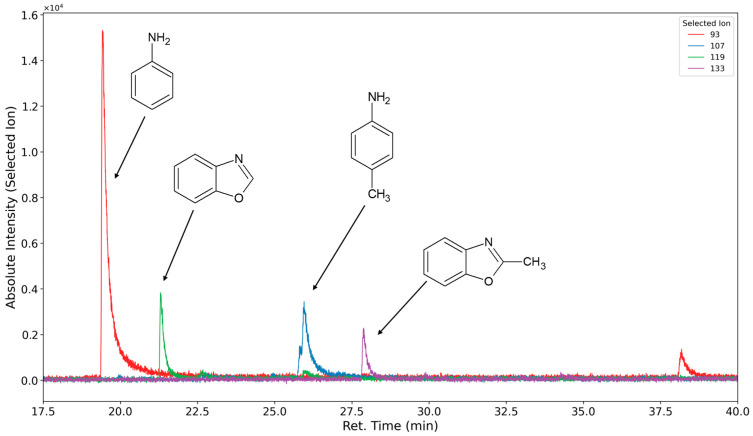
Selected ion chromatograms (*m*/*z* = 93 in red, 107 in blue, 119 in green, and 133 in purple) of polyurethane sample flushed with nitrogen at 120 °C for one week.

**Figure 15:**
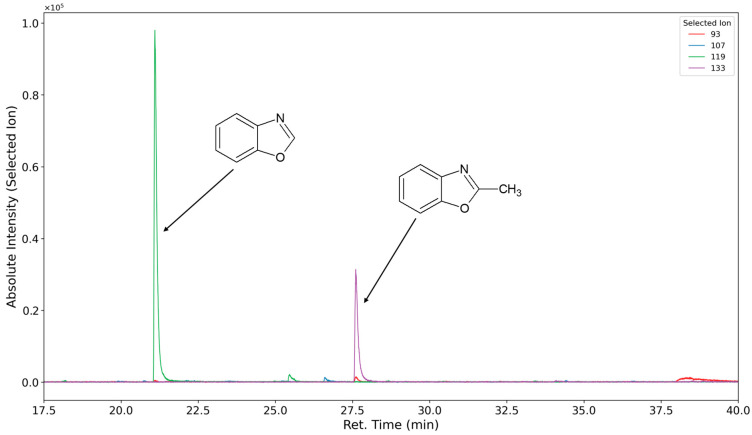
Selected ion chromatograms (*m*/*z* = 93 in red, 107 in blue, 119 in green, and 133 in purple) of polyurethane sample thermo-oxidized with oxygen at 120 °C for one week.

In Figure 14 (inert atmosphere) and Figure 15 (oxygen atmosphere), the selected ion chromatograms of the ions with *m/z* = 93 (caused by aniline ionization (RT 20.5 min)), the ions with *m/z* = 107 (caused by toluidine ionization (RT 25.9 min)), the ions with *m/z* = 119 (caused by benzoxazole ionization (RT 22.2 min)), and the ion with *m/z* = 133 (caused by methyl benzoxazole ionization (RT 28.7 min)) are shown.

Toluidine displays a double peak that is not baseline resolved. This might be due to the formation of both o- and p-toluidine. An additional yet unidentified oxidation product (assumed molecular mass 121 g/mol) assumed to be formed of the hard segment is detected at 39.1 min (Figure 15). Formanilide and 4-aminobenzaldehyde, both compounds with a molecular mass of 121 g/mol, were synthesized for retention time comparisons. Neither compound showed the same retention time, nor was it observed in the emissions. The following table shows qualitatively identified compounds emitted in the oxidative degradation experiments (seven-day equilibration period at 120 °C within a constant air stream) of the polyurethane foam (Table 3). Compounds listed as polyether-related products in Roman letters are products of the breakdown of the PPO segment, while PEO segment fragments are written in italic text.

#### 3.1.5. Classification of Emittents as Oxidation Products

**Figure 16:**
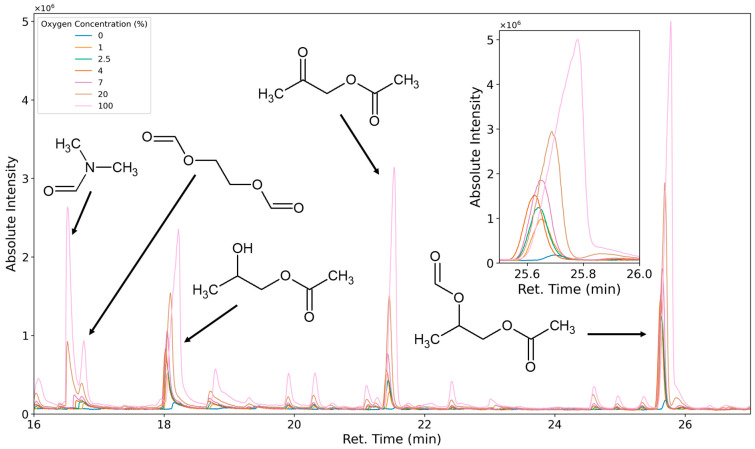
Chromatogram of selected peaks observed as emissions under 0% oxygen (blue), 1% oxygen (orange), 2.5% oxygen (green), 4% oxygen (brown), 7% oxygen (purple), 20% oxygen (beige), and 100% oxygen (pink).

To characterize VOCs as thermal degradation products or oxidative degradation products, the carrier gas/purging gas/reactant gas composition was varied. The test specimen was flushed and equilibrated with 0%, 1%, 2.5%, 4%, 7%, 20%, and 100% oxygen (the residual atmosphere was nitrogen), and the emissions were tested. When equilibrating the system with nitrogen, only a few compounds, such as aniline and toluidine, are emitted at higher levels than under oxidation. A variety of additional compounds are formed when oxygen is introduced. These compounds’ emission rates greatly increase when pure oxygen is used as the carrier gas (Figure 16). At 0% oxygen, the peak areas of compounds like 1,2-propanediol-1-acetate and 1,2-propanediol-1-acetate-2-formate are only small. This could be caused by minor oxygen contaminations of the analytical system or thermal degradation leading to the same compounds. It is also possible that low amounts of these compounds are only very slowly desorbed from the foam sample due to small dead volumes in the sample. All analytes in Table 3, except for 1,4-dioxane, 2,5-dimethyl-1,4-dioxane, aniline, isoquinoline, acridine, and diazabicyclooctane, were classified as oxidation products through atmosphere variation.

### 3.2. Reproducibility

Common analytical methods suffer from low reproducibility. This can be attributed to irreproducible sample properties, such as the sample surface, humidity, tortuosity, geometry, initial loading, temperature, and age. Additionally, the sampling procedure can suffer from temperature variations, volume and volume flow differences, and irregularities in the adsorption medium. Lastly, the thermodesorption of the GC injector system adds another error, as leakages between the thermodesorption tube and the analytical device can detrimentally impact the analytical results.

To investigate the reproducibility of this method, its overall reproducibility was investigated through five measurements on five foams. The data encompass the inhomogeneity of the original foam slab, the reproducibility of sample preparation (inter-sample comparability), the reproducibility of gas sampling, and the TD-GC-MS’s system error (intra-sample reproducibility).

**Figure 17:**
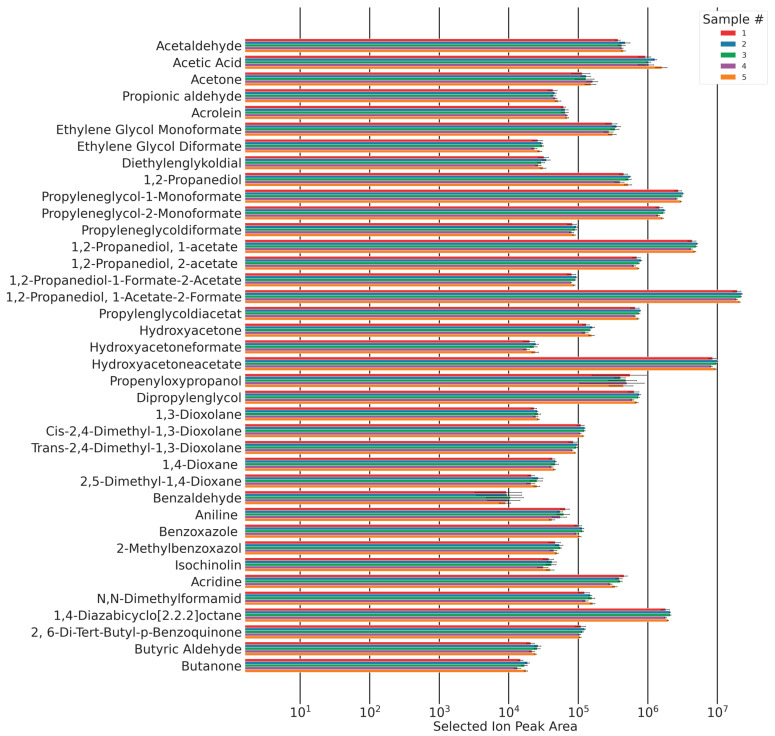
Chromatographic peak area and confidence intervals (standard deviation (*n* = 5)) for all qualified analytes for five samples of the same composition.

The total sampling duration added up to 100 min. Within this time interval, the peak areas of each sampling showed no trend and remained approximately constant (Figure 17). This shows that due to the equilibration process, a steady state of autoxidation has been reached, and emissions were constant within this time frame. Five emission samples were taken of all five foam samples. The areas of the chromatographic peaks of the identified compounds are shown in Figure 17.

The majority of the relative standard deviations of the analytes’ peak areas are <10%. Relative standard deviations increase with a decrease in observed concentrations.

### 3.3. Results of Sampling Time Variation

**Figure 18:**
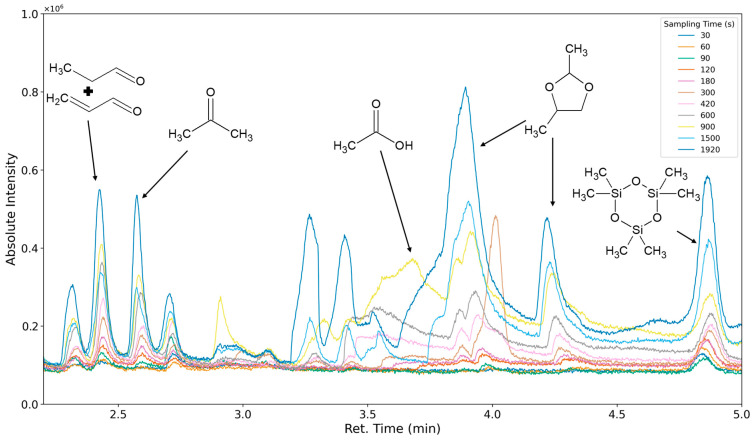
Overlay of chromatograms with various sampling times for retentions times of two to five minutes. Sampling times have been varied between 30 s and 1920 s.

**Figure 19:**
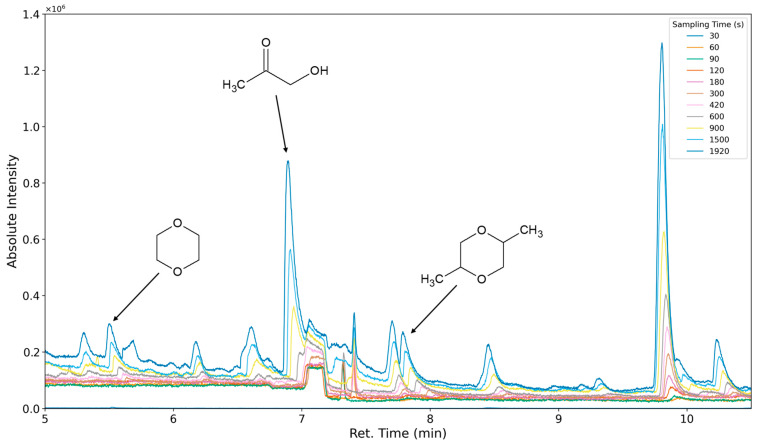
Overlay of chromatograms with various sampling times for retention times of five to ten and a half minutes. Sampling times have been varied between 30 s and 1920 s.

**Figure 20:**
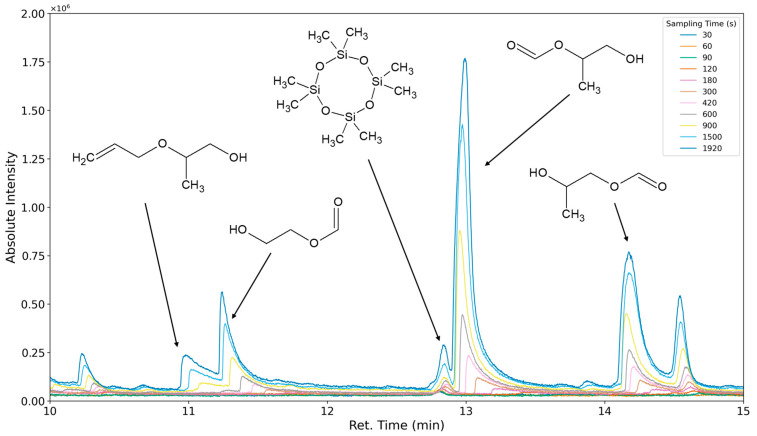
Overlay of chromatograms with various sampling times for retention times of ten to fifteen minutes. Sampling times have been varied between 30 s and 1920 s.

**Figure 21:**
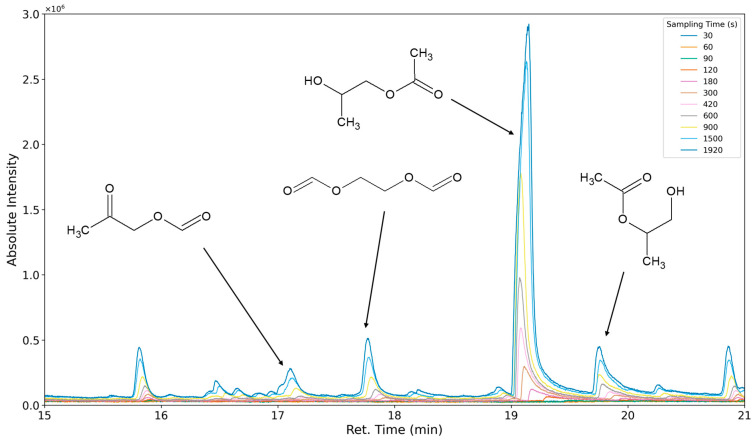
Overlay of chromatograms with various sampling times for retention times of fifteen to twenty-one minutes. Sampling times have been varied between 30 s and 1920 s.

**Figure 22:**
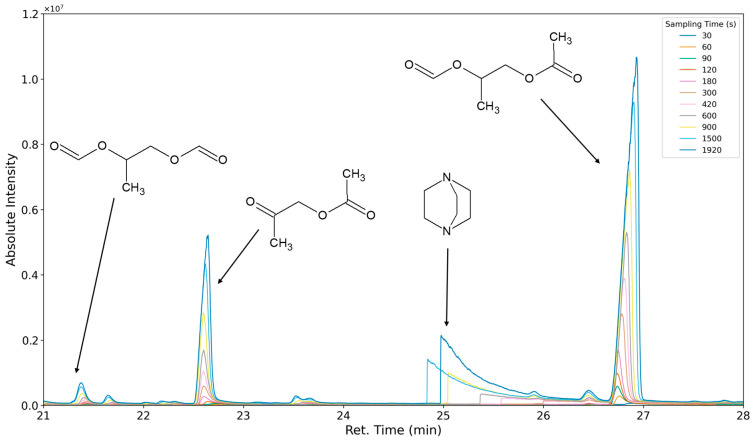
Overlay of chromatograms with various sampling times for retention times of twenty-one to twenty-eight minutes. Sampling times have been varied between 30 s and 1920 s.

**Figure 23:**
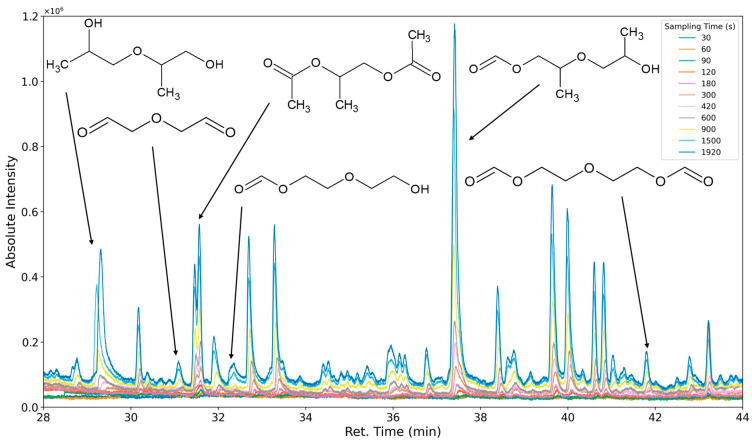
Overlay of chromatograms with various sampling times for retention times of twenty-eight to forty-four minutes. Sampling times have been varied between 30 s and 1920 s.

**Figure 24:**
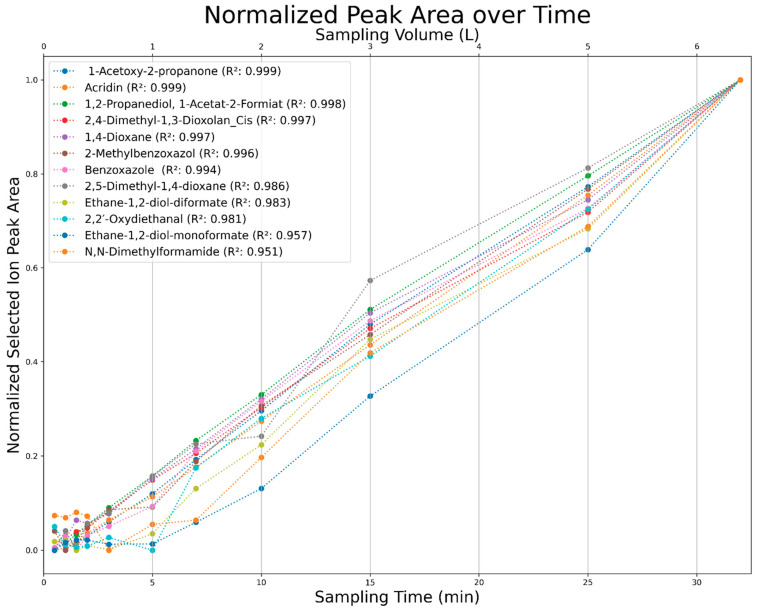
Normalized selected ion peak area over sampling time (min) and sampling volume (L) for a selection of analytes.

The aforementioned heteroscedasticity can be remedied by longer sampling times for analytes with low chromatographic peak areas (Figure 18, Figure 19, Figure 20, Figure 21, Figure 22, Figure 23 and Figure 24). In Figure 18 the chromatograms of a selection of qualified very volatile organic compounds (VVOCs) and VOCs with different sampling times are shown. Unfortunately, acetic acid does not produce well-defined Gaussian peaks in the GC-MS chromatogram. Figure 19 reveals the two formed dioxanes, 1,4-dioxane from polyethylene oxide and 2,5-dimethyl-1,4-dioxane from polypropylene oxide. Additionally, hydroxyacetone can be detected. The peak observed at 9.7 min could not be identified. Its mass spectra show a potential molecular ion peak at *m*/*z* = 74; therefore, it could be an isomer of hydroxyacetone, potentially lactaldehyde, or its formate ester. The irregular shape of the background from 7.0–7.1 min is a fragment of the thermodesorption system’s valve position changing. Figure 20 and Figure 21 show the esters of propylene glycol and ethylene glycol as well as a cyclic siloxane. In Figure 22 further esters can be seen and the emission of DABCO (only used in a catalyst in this foam). Figure 23 illustrates a variety of peaks caused by higher boiling species. The identified compounds here are dipropylene glycol and propylene glycol diacetate.

All observed analytes increase in the chromatographic peak area with increased sampling time. This allows for the accumulation of analytes over long periods, consequently allowing for an analysis above the limits of detection and quantification for nearly any analyte. This process is only limited by the breakthrough volumes of extremely volatile compounds. These compounds, however, due to their high volatility, can be analyzed at lower volume flows to allow for longer sampling times. It can also be seen that some compounds require over five minutes of sampling time to provide a sufficient signal-to-noise ratio (Figure 24).

### 3.4. Analyte Quantification

For a selection of analytes shown in Figure 17, calibrations were conducted to generate quantitative results of the respective analyte’s molar emission rates. The synthetic procedures for commercially unavailable compounds and the calibration data are found in Supplemental Material II.

## 4. Discussion

Typical polyurethane foam emission analyses in the automotive industry are bag or chamber methods, as in ISO 12219 [44], or miniature flow methods, like the VDA278 method. As in real life, these methods do not discriminate between initial loading, diffusion, and degradation processes. Therefore, they do not allow for the investigation of oxidation rates as a basis for understanding degradation mechanisms.

If the method described here is used to determine the initial loading of contaminations in a freshly synthesized foam, the collected analyte’s mass is much higher than that collected using contemporary analysis methods. This is due to the acceleration through convective transport rather than diffusion. Therefore, the application of this method to quantify the degradation products of a material offers huge benefits in speed and precision as required for the development of recipes with improved stability or for the introduction of materials from recycling.

A temperature of 120 °C is excessive compared to real use and for the known stability of polyurethanes in flexible foams, including, in particular, for the expected allophanate, biuret, and hydrogen bonding substructures. In a further, not yet published study, we investigated emissions over a wide range of temperatures (65 °C to 155 °C). Formation and emission rates strongly increase with increased temperature. Given sufficient equilibration time and sampling time, the majority of emissions can be expected to be observable at any temperature within that range. At decreased temperatures, investigating the same selection of analytes as presented here requires sampling times that increase linearly with the drop of formation/emission rates. Additionally, the equilibration of samples at lower temperatures requires a longer time. Our choice of temperature reflects this fact.

The possibility to run experiments under different atmospheric conditions allows for in-depth research into degradation mechanisms. In this study, we have chosen to present an unambiguous example: the difference in the formation and emission of analytes with oxygen concentration variations. Other atmospheric changes can easily be investigated, like ozone concentrations, humidity, NO_x_, and many more. The majority of analytes reported in this work increase in emissions when the samples are exposed to increased oxygen concentrations.

As the emissions stabilize in the steady state of polymer autoxidation, reproducible quantification becomes possible. The same reproducibility can be expected for the initial loading of contaminations, although these cannot be repeatedly measured as their concentrations rapidly change when the materials are purged. The measured emissions’ accuracy can be assumed to be constant for any phase of the foam’s history.

The method allows for the nearly unlimited accumulation of analyte mass through the variation of the sampling time. As the observed analytes are formed and emitted at a constant rate, there is a clear linear correlation between the chromatographic peak area and the sampling time. This feature is only limited by the breakthrough volume of some lower molecular analytes; however, those are potentially analyzed using thermo-desorption tubes filled with a stronger adsorbent or through chemisorption using silica cartridges filled with dinitrophenylhydrazine in the case of aldehydes and ketones.

The emissions comprise compounds associated with the soft segment, the hard segment, and catalysts. The majority of the detected compounds are derivatives of the polyetherol, such as low molecular weight aldehydes, acetic acid, dioxolanes, dioxanes, and esters of oxidized and non-oxidized derivatives of 1,2-ethanediol and 1,2-propanediol. The most prominent peaks are oxidation products of the PPO segment. This fact can be attributed to the polyether polyol compromising ~80% polypropylene oxide and its general lower stability towards oxidation than PEO. Associated with the isocyanate component are aniline, benzoxazole, 2-methyl benzoxazole, isoquinoline, and acridine.

The portfolio of volatiles formed during autoxidative breakdown of polyurethanes encompasses very volatile organic compounds, volatile organic compounds, and semi-volatile organic compounds. Very volatile organic compounds have low breakthrough volumes on thermo desorption tubes and can therefore not be reliably sampled. However, some of the VVOCs formed during the autoxidative breakdown of polyurethanes are carbonyle functional. Those compounds can reliably be accumulated on sampling cartridges using dinitrophenylhydrazine (DNPH). While sampling and analysis with thermo-desorption tubes and GC-MS are not analyte functionality selective, the DNPH method is selective for carbonyle compounds only. Therefore, in order to qualify and quantify all emissions, both methods need to be applied.

**Figure 25:**
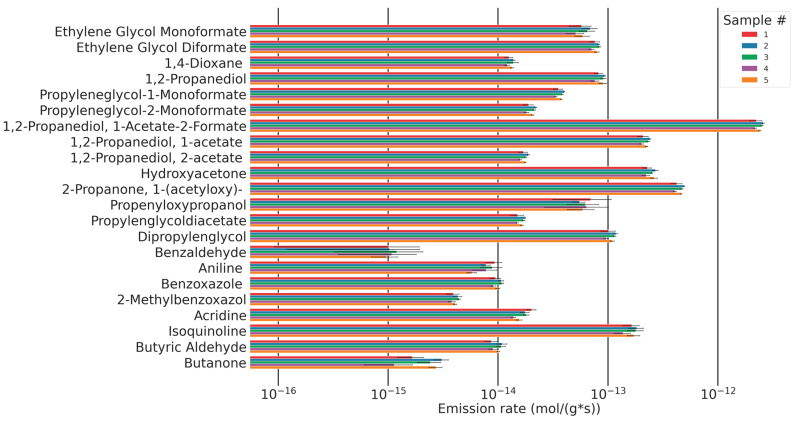
Molar emission rates and confidence intervals (standard deviation (*n* = 5)) for a selection of quantified analytes for five samples of the same composition.

A formerly published study investigating the emissions of formaldehyde and acetaldehyde using DNPH cartridges from the same samples investigated here, under the same flow conditions at 120 °C, found formaldehyde emissions of ~1.2 pmol/g·s and acetaldehyde emissions of 5.5 pmol/g·s [9]. In the study presented here, the main emission observed is 1,2-propanediol-1-acetate-2-formate with 2.4 pmol/g·s. This analyte has been reported before in the thermo-oxidative breakdown of polyethers, but, to our knowledge, never before in polyurethane foams [14]. The average molar emission rate of 1,2-propanediol-1-acetate-2-formate is twice the rate of formaldehyde (1,2-propanediol-1-acetate-2-formate: 2.4 pmol/g·s, formaldehyde: 1.2 pmol/g·s), five times higher than acetyloxyacetone, and ten times higher than the emission of hydroxyacetone. Other molar emissions are at least 25 times lower than 1,2-propanediol-1-acetate-2-formate (see Figure 25).

The total emission of quantified analytes resulting from the degradation of the soft segment at 120 °C is 3.793 ± 0.204 pmol/g·s. Assuming constant formation and emission rates, this corresponds to emissions of 6.88 µmol/g foam over three weeks of accelerated ageing at 120 °C. This relates to 10 mmol ether bonds per gram of foam. A total of 0.07% of ether bonds are broken and lead to VOC emissions. The ratio of the number of hydroperoxide groups leading to VOCs to the number of hydroperoxide groups leading to terminal esters, chain esters, alcohols, or chain branching is unknown. Therefore, this number is a minimum of broken ether bonds. It has been reported before that 1 mol of dibutyl ether carrying a hydroperoxide group degrades to 0.5 mol of butyl formate, 0.2 mol butanol, and 0.2 mol butanal. These three products at least indicate chain scission with a possible follow-up reaction leading to the formation of VOCs. Only 0.2 mol of butylbutyrate were formed which indicate no chain scission [10]. It is questionable whether or not these numbers can be applied to provide a ballpark estimate of the amount of hydroperoxides in polyurethane leading to VOCs.

A distinguishable feature of the chromatograms is that the majority of identified compounds are derivatives of glycol monomers. The emissions do not exhibit a continuous molecular mass distribution. If a purely statistical random chain scission mechanism caused the oxidative breakdown of the polymer chain, a higher abundance of compounds with higher molecular mass would be expected. This analytical method is ‘blind’ for any chain scission that does not produce volatile compounds; however, it is still noticeable that the highest emissions were observed for 1,2-propanediol-1-acetate-2-formate, acetyloxyacetone, hydroxyacetone, and 1,2-propanediol-1-acetate. The corresponding dipropylene glycol derivatives were not observed, nor are there any potential peaks in the chromatograms potentially representing those compounds. This is contrasted by the fact that the measurement of small emissions of compounds that derive from propylene glycol oligomers up to pentapropylene glycol was accessible.

The prevalence of low molecular weight derivatives of glycols might indicate a mechanism that favors their formation over higher mass derivatives.

Only a few analytes were qualified that derived from the hard segment of the polyurethane matrix. Aniline, toluidine, benzaldehyde, benzoxazole, 2-methylbenzoxazole, isoquinoline, and acridine were observed; however, their emissions are generally low. Acridine is a side product of MDI production formed from 2,2′-MDA [45]. The emissions of aniline and toluidine are lowered under oxidizing conditions. We assume an oxidation reaction to take place after their formation that substantially lowers the compounds’ volatility. This behavior does not allow us to investigate whether or not oxidizing conditions increase the formation of the aromatic amines themselves. They are continuously emitted under inert conditions, allowing for the conclusion that they are formed through thermal degradation.

## 5. Conclusions

The new analytical method presented in this paper significantly improves upon the results obtained using the ISO 12219 or VDA278 standard methods in terms of interpretability. The origin of emissions can be directly identified as initial loading or as an oxidative degradation product. Additionally, this method allows for extraordinarily high reproducibility of the analysis of analytes. The standard deviations of repeated measurements are commonly around or below 10%, with some analytes presenting with standard deviations as low as 1%. The use of forced convection enables reproducible qualitative and quantitative analysis of emissions from the entire volume of open-celled polyurethane foam materials.

New calibration methods for analytes have been introduced, and several previously unreported polyurethane oxidation products have been qualified, with their synthesis routes detailed. Importantly, the desired experimental conditions, i.e., atmosphere composition or temperature, of foam aging can be adapted precisely to the research requirements. The results that can be generated with this method are independent of external factors, such as the initial loading of the flexible foam sample or the adsorption of pollutants from the atmosphere. They provide quantitative insight into the compounds produced by the thermal and thermo-oxidative degradation of the polymer.

The variation of sampling times allows for an even more in-depth analysis of volatile compounds with very low emission rates or particularly low detector responses. By increasing the sampling time, the limits of qualification and quantification can be progressively lowered until an analyte’s breakthrough volume on the adsorbent tube is reached. If the accumulation of sufficient analyte mass is limited by the analyte’s breakthrough volume, the volume flow can be lowered or a stronger adsorbent can be used. An analyte with a low breakthrough volume is unlikely to be strongly adsorbed to the foam surface, as these compounds generally have a high vapor pressure. In this context, it has to be mentioned that VOCs in extremely low concentrations can contribute to the smell of a material. This research allows us to investigate several analytes that have not been evaluated for their olfactory profile before. This approach appears particularly useful in the analysis of compounds like aniline or dimethylformamide to evaluate potential human exposures caused by polymer degradation.

This work yields a tool to conduct targeted research on the degradation mechanisms of gas-permeable polyurethane soft foams. This is a necessary technology to perform targeted optimization of polyurethane formulations.

## Figures and Tables

**Table 1:** Foam formulation at index 90 and molar amounts of different ether moieties.

Polyether Polyol Mixture	g/mol	Mass [g]	mmol	mmol OH/NH	mmol PO	mmol EO
Propylene glycol ← PO58 ← EO13	4000	241.0	60	121	3073	663
Glycerol ← PO87 ← EO19	6000	120.5	20	60	1908	362
Glycerol ← PO70 ← EO15	4800	11.5	2	7	182	34
Water	18	18.3	1017	2033		
Triethanolamine	149	1.0	7	20		
Triethylenediamine	112	1	0.009			
Dabco NE300 (Evonik)	203	0.4	2	2		
Polyether-modified polydimethylsiloxane Tegostab 8734 LF 2 (Evonik)	500	3.7	7	7	unknown	unknown
TOTAL		396.4		2251	5163	1058
**Isocyanate**				**mmol NCO**		
A blend of 4,4′-MDI, 2,4′-MDI, 2,2′-MDI, and polymeric MDI (2,2 mol NCO/mol)		253.5		2028		

**Table 2:** Analytes synthesized in situ from alcohols and carboxylic acids.

Target Analyte	Limiting Compounds	Excess Compound/Solvent
Monomer esters		
1,2-Ethanediol monoformate	Formic acid	1,2-Ethanediol
1,2-Ethanediol diformate	1,2-Ethanediol	Formic acid
1,2-Propanediol-1-formate	Formic acid	1,2-Propanediol
1,2-Propanediol-2-formate	Formic acid	1,2-Propanediol
1,2-Propanediol diformate	1,2-Propanediol	Formic acid
Hydroxyacetoneformate	Hydroxyacetone	Formic acid
Dimer esters		
Diethylene glycol monoformate	Formic acid	Diethylene glycol
Diethylene glycol diformate	Diethylene glycol	Formic acid
Dipropylene glycol monoformate	Formic acid	Dipropylene glycol
Dipropylene glycol diformate	Dipropylene glycol	Formic acid
Trimer esters		
Triethylene glycol monoformate	Formic acid	Triethylene glycol
Triethylene glycol diformate	Triethylene glycol	Formic acid
Tripropylene glycol monoformate	Formic acid	Tripropylene glycol
Tripropylene glycol diformate	Tripropylene glycol	Formic acid

**Table 3:** Volatile products detected through TD-GC-MS. Structures related to the ethylene oxide end block segment are in italics. Reference identification is based on NIST database comparison. Synthetic reference means that the compound has been synthesized by us for reference. The emission rate is given as yield in fmol·g^−1^·s^−1^, and one standard deviation of the five measurements is given as the confidence interval. The identification method is given as (a) identified based on commercial reference; (b) identified based on NIST mass spectral database reference; or (c) identified based on synthesized reference.

Peak #	Identified Compound	Retention Time (min)	M (g/mol)	Selected Ion	Yield (fmol/g·s) ± 1 σ
	Polyether related products				
1 ^(a)^	Acetaldehyde	2	44	44	
2 ^(a)^	Acetic acid	6	60	60	
3 ^(a)^	Propionic aldehyde	2.4	58	29	
4 ^(a)^	Acrolein	2.4	56	56	
5^(a)^	Acetone	2.58	58	43	
6 ^(c)^	cis-2,4-Dimethyl-1,3-dioxolane	3.6	102	87	
7 ^(c)^	trans-2,4-Dimethyl-1,3-dioxolane	3.9	102	87	
8 ^(c)^	*1,4-Dioxane (no oxidation product)*	5.33	88	88	13.1 ± 0.8
9 ^(c)^	2,5-Dimethyl-1,4-dioxane (no oxidation product)	7.53	116	116	
10 ^(c)^	*Ethyleneglycolmonoformate*	10.4		60	60 ± 10
11 ^(c)^	*Ethyleneglycol diformate*	17.4		72	79 ± 4
12 ^(c)^	*Diethyleneglycol dialdehyde*	30		102	
13 ^(a)^	1,2-Propyleneglycol	7.5	76	45	87 ± 6
14 ^(a)^	Hydroxyacetone	6.73	74	74	248 ± 16
15 ^(c)^	Propenyloxypropanol	11.1	116	45	62 ± 24
16 ^(c)^	1,2-Propyleneglycol-1-formate	13		45	37 ± 1
17 ^(c)^	1,2-Propyleneglycol-2-formate	14.2		45	20 ± 1
18 ^(c)^	Hydroxyacetone formate	16.85		102	
19 ^(c)^	Hydroxyacetone acetate	22.19		43	451 ± 18
20 ^(c)^	1,2-Propyleneglycol-1-acetate	18.86	118	43	222 ± 10
21 ^(c)^	1,2-Propyleneglycol-2-acetate	19.5	118	43	17 ± 1
22 ^(c)^	1,2-Propyleneglycol diformate	21	132	60	
23 ^(c)^	1,2-Propyleneglycol-1-formate-2-Acetate	26	146	87	
24 ^(c)^	1,2-Propyleneglycol-1-acetate-2-formate	26.4	146	43	2386 ± 106
25 ^(a)^	1,2-Propyleneglycol diacetate	31.35	160	43	16 ± 1
26 ^(a)^	Dipropyleneglycol	29.5		59	108 ± 7
27^(b)^	Propenyloxydipropyleneglycol	32.3		41	
	Isocyanate related compounds				
28 ^(a)^	Aniline (no oxidation product)	20		93	8 ± 1
29 ^(a)^	Benzaldehyde	21.55		106	1 ± 0.7
30 ^(a)^	Benzoxazole	21.83		119	10 ± 1
31 ^(a)^	2-Methylbenzoxazole	28.39		133	4 ± 0.3
32 ^(a)^	Isoquinoline (no oxidation product)	37.8		129	167 ± 26
33 ^(a)^	Acridine (no oxidation product)	64		179	17 ± 1.25
	Additive-related compounds				
34 ^(a)^	Diazabicyclooctane (no oxidation product)	26.1		112	
35 ^(a)^	BHT (oxidized)	53.43		220	
36 ^(a)^	Dimethylformamide	18	73	73	

## Data Availability

The data presented in this study are available upon request from the corresponding author due to being subject to further publications.

## Supplementary Materials

The following supporting information can be downloaded at https://www.mdpi.com/article/10.3390/polym16233342/s1, Supplementary Material I: Reference material synthesis and characterization (docx); Supplementary Material II: External calibration method and graphs (docx).
