# Supplementary material for: The Thermo-Oxidative Degradation of Polyurethane Open-Cell Soft Foam Investigated Through Gas Chromatography and Mass Spectrometry of Volatile Organic Compounds
Source: Polymers (Basel). 2024 Nov 28;16(23):3342. doi: 10.3390/polym16233342 (PMC11644683; doi:10.3390/polym16233342)
Supplement: Supplementary file 1 [file polymers-16-03342-s001.zip › Supplemental Material I - Reference material synthesis.pdf]

The Thermo-Oxidative Degradation of Polyurethane Open-Cell Soft Foam Investigated Through Gas Chromatography and Mass Spectrometry of Volatile Organic Compounds

Authors:

Christian Stefan Sandten M.Sc.[a,*] C.Sandten@FH-Muenster.de
Prof. Dr. Martin Kreyenschmidt[a] Martin.Kreyenschmidt@FH-Muenster.de
Dr. Rolf Albach[b] Rolf.Albach@Covestro.com
Prof. Dr. Ursula E. A. Fittschen[c] Ursula.Fittschen@TU-Clausthal.de

a: University of Applied Sciences Muenster Hüfferstraße 27, 48149 Münster, Germany
b: Covestro Deutschland AG Kaiser-Wilhelm-Allee 60, 51373 Leverkusen, Germany
c: Clausthal University of Technology Adolph-Roemer-Straße 2A, 38678 Clausthal-Zellerfeld, Germany

*Corresponding author (Phone: +49 02551 962291)

Supplemental Material I – Synthesis of reference materials

In this supplemental section, we present the synthesis of numerous compounds suspected to arise from the oxidation of polyethers. This synthesis endeavor was necessitated by the intricate nature of their mass spectra, which often exhibit subtle distinctions that hinder definitive structural assignment. By generating synthetic reference materials and meticulously characterizing their retention times and mass spectra, we aimed to facilitate accurate identification. The comprehensive inclusion of all synthesized compounds in this study provides a foundational repository for future investigations, enabling researchers to navigate previously explored compounds and enhance the scope of inquiry. Additionally, providing the synthetic pathways for compounds not observed in our main study aims to prevent redundant investigations and supports efficient scientific progress.

If possible, the odour of the synthetic products has been assessed by a panel of volunteers.

Synthesis of 1,2-Propanediol-1-acetate-2-formate

Esterification of hydroxyacetone (observed) to acetoxyacetone (observed)

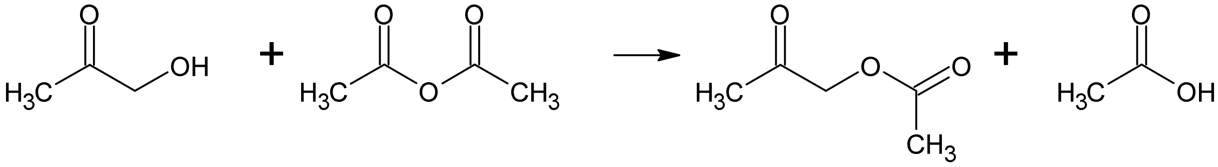

41.35 g of acetic anhydride (0.4 mol) and 4 drops of concentrated sulfuric acid are transferred into a 500 mL round bottom flask. The flask is cooled to 5 °C in an ice bath and 30 g of hydroxyacetone (0.4 mol) are slowly added using a dropping funnel while maintaining a reaction temperature below 20 °C. After full addition, the ice bath is removed and a magnetic heating stirring plate with an oil bath is added. The oil bath is heated to 100 °C and the reaction mixture is heated for three hours. After letting the mixture cool to room temperature, the mixture is poured into 100 mL of 0 °C water. The solution is saturated with sodium chloride and sodium bicarbonate and was extracted three times with dichloromethane. The organic phases were combined and dried with anhydrous sodium sulfate. The

dichloromethane was removed in a rotary evaporator under vacuum. The purity of the resulting pale-yellow liquid was checked by GC-MS. No other compounds were detected.

Odor: Hydroxyacetone: mildly unpleasant, sour and musty/stale. Acetoxyacetone: foot odor, vomit and rancid butter.

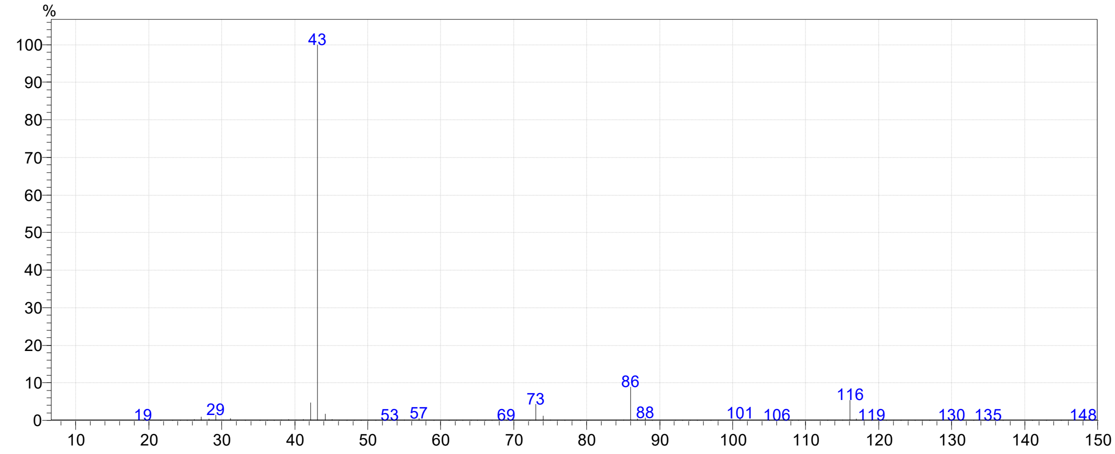

Figure S 1 Mass spectra of acetoxyacetone

Reduction of acetoxyacetone to 1,2-propanediol-1-acetate (observed)

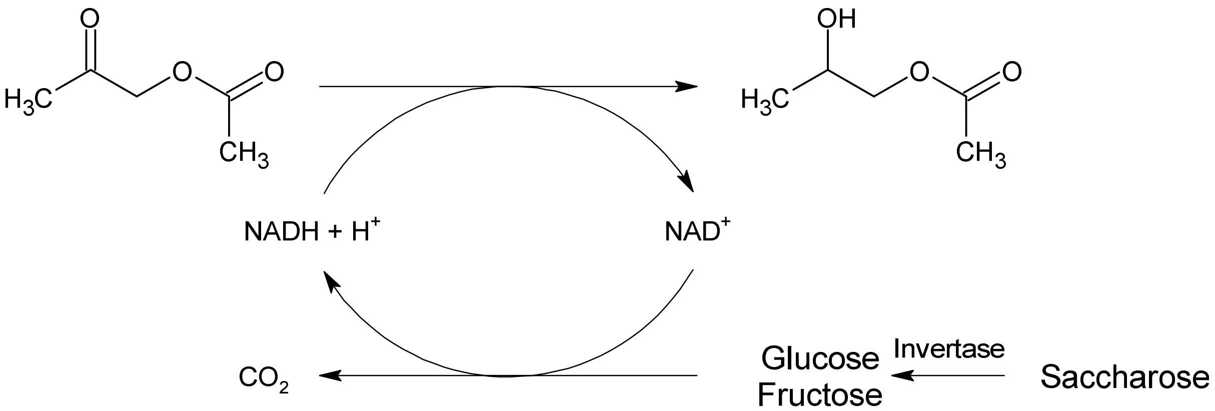

Various attempts with sodium borohydride lead to intramolecular transesterification. We assume that the hydride addition and alkoxide formation promotes an ester group transfer through a cyclic transition state. To avoid this, an enzymatic reduction was performed. 100 g of saccharomyces cerevisae were added to a 2 L round bottom flask with 800 mL of tap water to which 150 g of saccharose (0.44 mol) were added. The round bottom flask was equipped with a three neck adapater to which an overhead stirrer, a fermentation lock and a stopper were added. The yeast suspension was heated to 30 °C with a water bath for one hour. Afterwards, 10 g of acetoxyacetone (0.09 mol) were added and the mixture was stirred for 24 h at room temperature. After 24 hours a solution of 150 g of saccharose (0.44 mol, total of 0.88 mol) and 800 mL tap water was added. Then 15 g of acetoxyacetone (0.13 mol, total of 0.22 mol) were added and the mixture was stirred for another three days. Afterwards, the stirring was turned off and the yeast was allowed to settle for 24 h. The supernatant was decanted, separated into centrifuge vials, and centrifuged at 4000 rpm for ten minutes to precipitate freely floating yeast at the bottom of the vials. Afterwards, a liquid-liquid extraction with 15 times 30 mL of dichloromethane was conducted. During the extraction, the mixture tends to form stable emulsions that can be broken with a centrifuge. The organic phases were combined, dried with

anhydrous sodium sulfate, filtered and stripped of the dichloromethane using rotary evaporation. The purity of the resulting pale-yellow liquid was checked by GC-MS. No other compounds were detected.

Odor: no strong smell, mildly rancid and/or rotten.

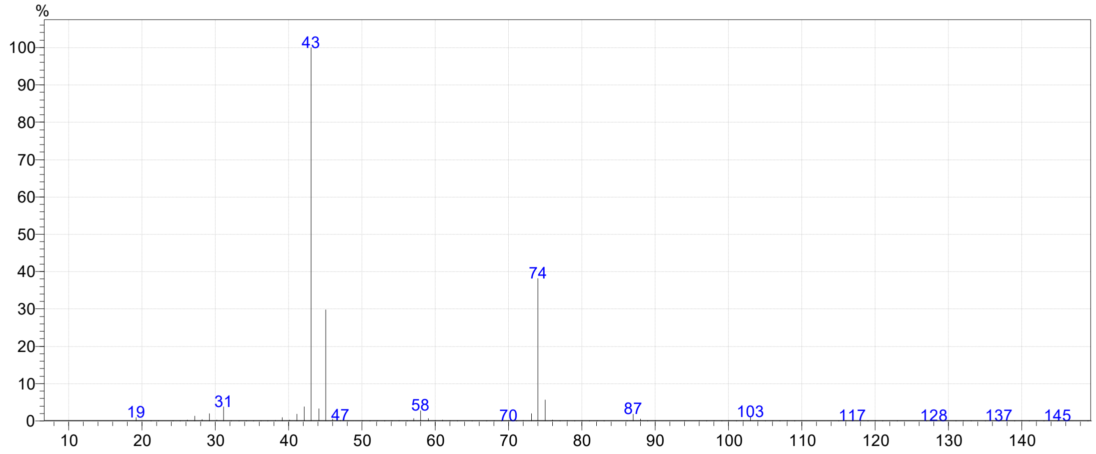

Figure S 2 Mass spectra of 1,2-propandiol-1-acetate

Esterification of 1,2-propanediol-1-acetate to 1,2-propanediol-1-acetate-2-formate (observed)

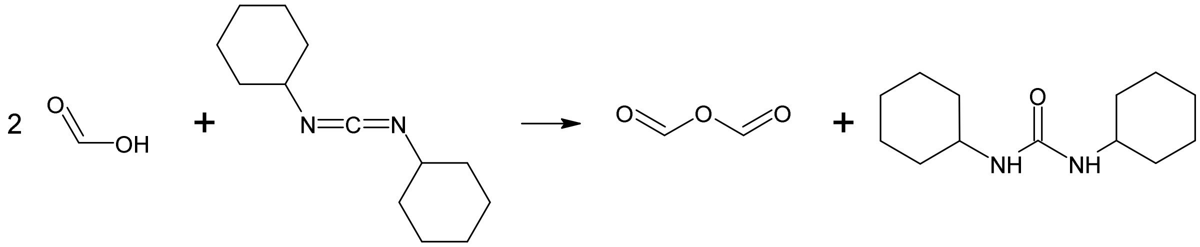

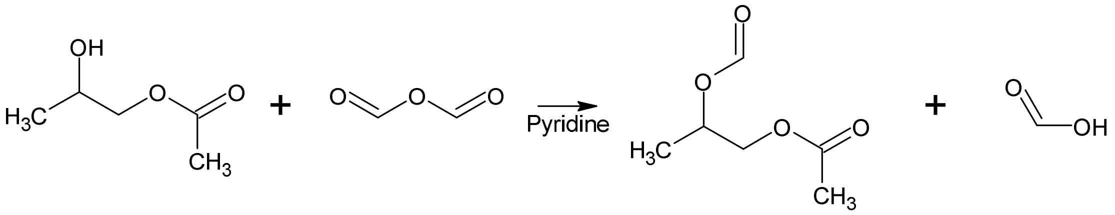

As 1,2-propanediol-1-acetate is prone to transesterification under acid catalysis the addition of formic acid was not possible. Therefore, a base-catalyzed esterification with formic acid anhydride using pyridine as catalyst was done. Formic acid anhydride is not stable at room temperature. Therefore, the reaction mixture was chilled to -20° C.

27.29 g of formic acid (0.59 mol) were poured into a glass beaker and dissolved in 120 mL of diethylether. The solution was cooled to -20 °C in an ice sodium chloride bath. Then 61.1 g of N,N'-dicyclohexylcarbodiimide (0.295 mol) were dissolved in 225 mL of diethylether and cooled down to -20 °C. The N,N'-dicyclohexylcarbodiimide solution was slowly poured into the formic acid solution and stirred for three hours below a temperature of -5 °C. After three hours the mixture was filtered while maintaining a temperature below -5 °C to remove the formed dicyclohexylurea.

A solution of 7 g of 1,2-propanediol-1-acetate (0.059 mol) with 56 g of pyridine (0.71 mol) was created and cooled to -20 °C. The former solution of formic acid anhydride in diethylether was slowly poured into the pyridine solution while maintaining a temperature below -5 °C. After complete transfer of the anhydride solution the mixture is stirred for another hour and filtered due to the formation of

additional dicyclohexylurea. The solution is allowed to return to room temperature. The excess of formic acid anhydride decomposes to carbon monoxide and formic acid. The formation of bubbles can be observed. When the formation of bubbles ceases, the solution is transferred into a separation funnel. The pyridine is removed with dilute hydrochloric acid until the organic phase does not react basic to universal pH indicator strips anymore. The organic phase is dried with sodium sulfate and the ether is removed using a rotary evaporator. The yield was 8.1 g (93 %).

Odor: sharp acidic fermented fruit smell.

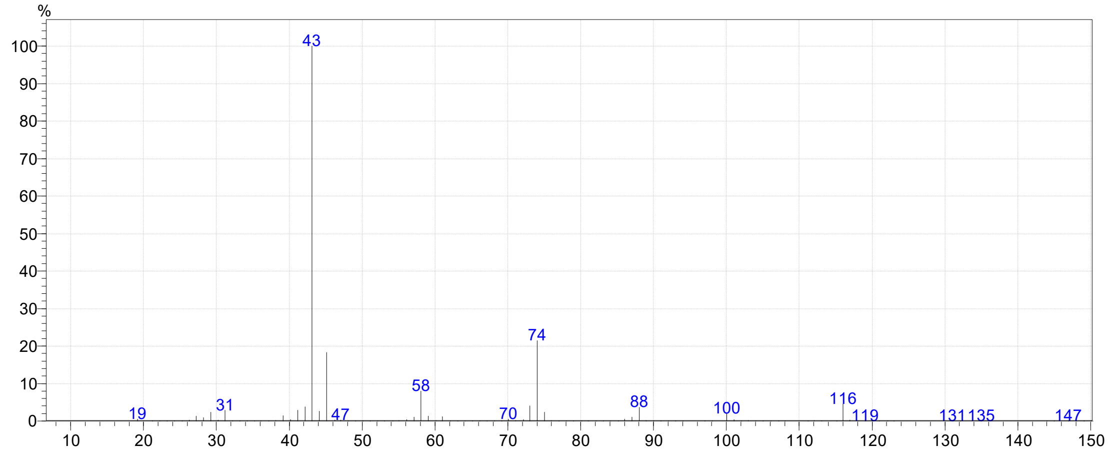

Figure S 3 Mass spectra of 1,2-propandiol-1-acetate-2-formate

Synthesis of Propenyloxypropanol / 1-(Allyloxy)-2-propanol

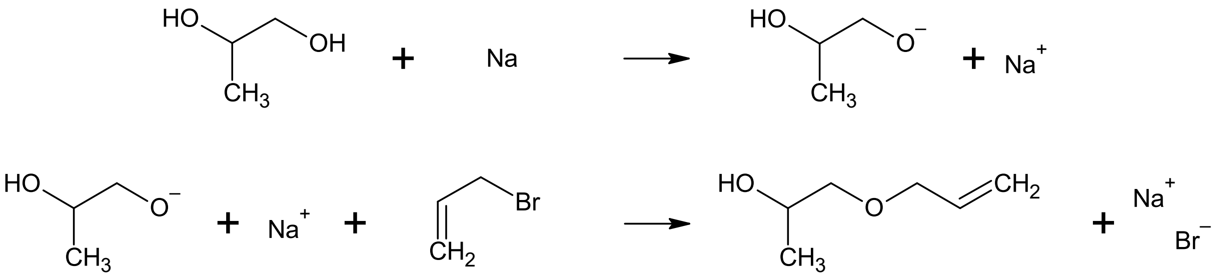

In a 100 mL two neck round bottom with a reflux condenser flask were transferred 45 g of propane-1,2-diol (0.6 mol). To this a total of 2.9 g of sodium (0.12 mol) was slowly added in small pieces. Then a dropping funnel with 24 g of allylbromide (0.1 mol) was added. This was slowly dropped into the round bottom flask over the course of an hour. The reaction mixture was kept at 70 °C for three hours. Then the reaction mixture was cooled to room temperature and the contents of the round bottom flask were transferred into a separation funnel with 250 mL of water. The resulting mixture was washed three times with MTBE, the organic phases were combined and dried with anhydrous sodium sulfate. The MTBE was removed via rotary evaporation and the resulting clear and colorless liquid was distilled at 90 °C at 100 mbar. The yield was 8.3 g (36 % yield with regards to allylbromide).

Odor: Unpleasant, burnt plastic

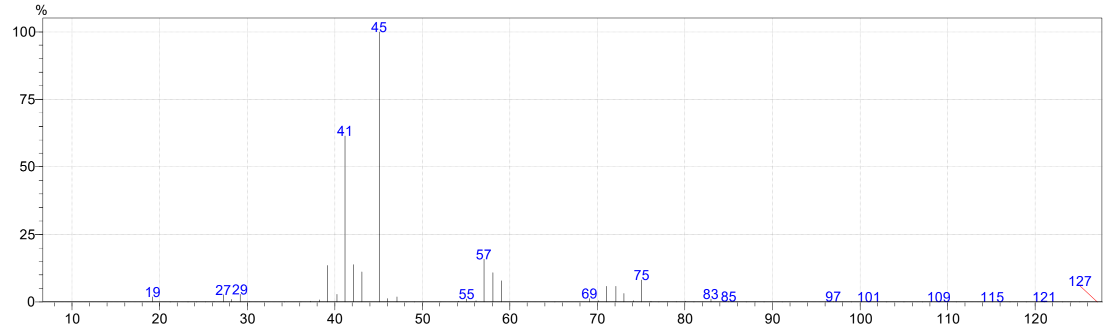

Figure S 4 Mass spectra of Propenyloxypropanol / 1-(Allyloxy)-2-propanol

Synthesis of dioxolanes

1,3-dioxolane

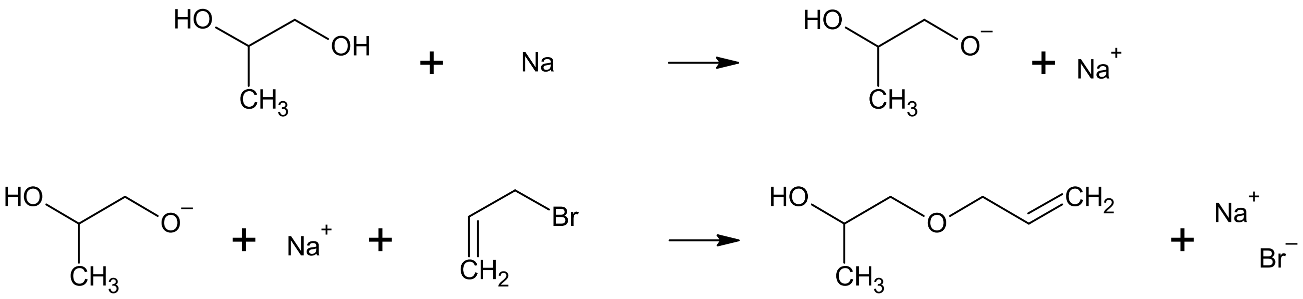

33.8 g of ethyleneglycol (0.54 mol) were transferred into a 100 mL round bottom flask. 8.57 g of paraformaldehyde (0.28 mol formaldehyde equivalent) were added. The mixture was cooled to 0 °C in an icebath and a drop of concentrated sulfuric acid was added. A stopper was inserted loosely in the flask and the mixture was heated to 90 °C. Upon heating the mixture clarified within 10 min at 90 °C. No formation of gas was observed. The reaction mixture was let cooled down to room temperature and stirred overnight. The next day a distillation apparatus and an oil bath were added. The oil bath was set to 120 °C and the distillate starting to come over at 80 °C head temperature was directly distilled into a separation funnel. 1,3-Dioxolane and water are miscible. After the head temperature dropped, a saturated NaC solution was added to the separation funnel which broke the mixture into a two-phase system. The water was separated and the organic phase was washed twice with saturated NaCl solution. The organic phase is dried with anhydrous sodium sulfate and filtered. The yield was 5.6 g (26.5 % yield with regards to paraformaldehyde).

Odor: Unpleasant, cold dry sweat, rancid.

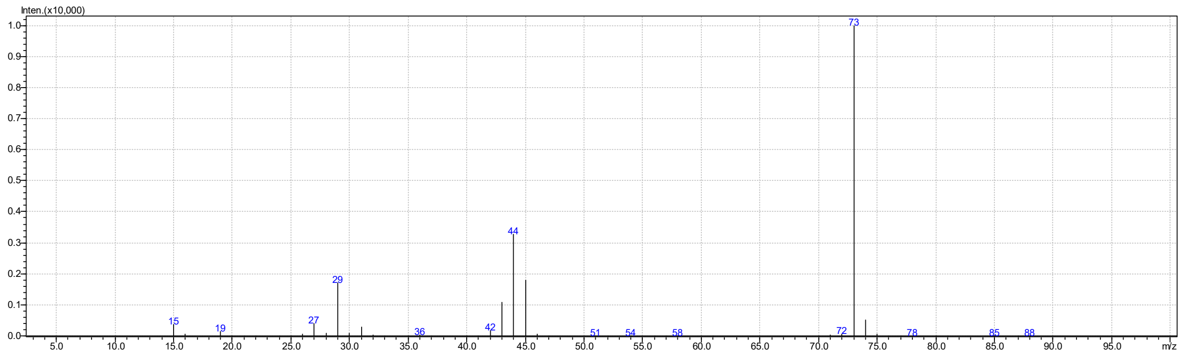

Figure S 5 Mass spectra of 1,3-Dioxolane

2-Methyl-1,3-dioxolane

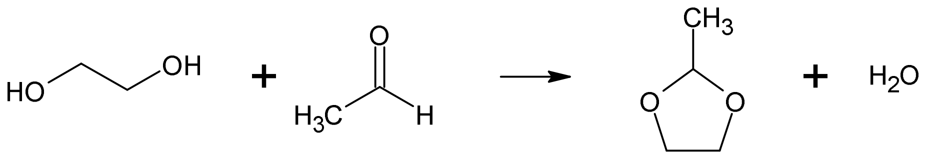

38 g of ethyleneglycol were added into a 100 mL round bottom flask and 13.3 g acetaldehyde were added. Upon addition the solution warmed up noticeably. The mixture was cooled to 0 °C in an icebath and a drop of concentrated sulfuric acid was added. The mixture turns a pale yellow after sulfuric acid addition. The reaction mixture is stirred overnight. On the next day a distillation apparatus is added and an oil bath at a 120 °C is used to heat the solution. At 72 °C a milky distillate comes over which separates into two phases in the separatory funnel. After the head temperature drops the distillate is washed twice with saturated NaCl solution. The organic phase is dried with anhydrous sodium sulfate and filtered. The yield was 15.08 g (57 % yield with regards to acetaldehyde).

Odor: rotten fruit, almond.

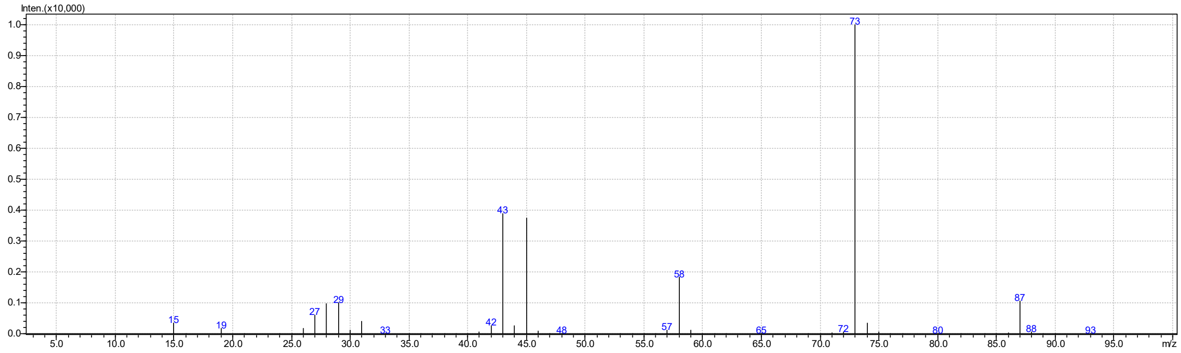

Figure S 6 Mass spectra 2-Methyl-1,3-dioxolane

4-Methyl-1,3-dioxolane

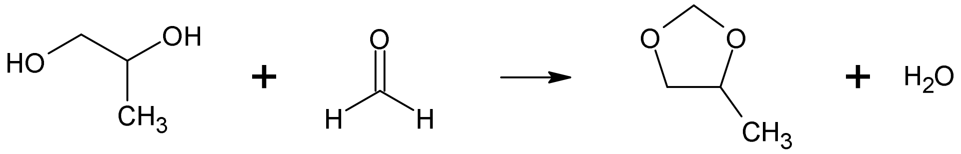

34.14 g of propyleneglycol (0.45 mol) were added into a 100 mL round bottom flask and 6.74 g of paraformaldehyde were added (0.22 mol formaldehyde equivalent). The mixture was cooled to 0 °C in an icebath and a drop of concentrated sulfuric acid was added. A stopper was inserted loosely in the flask and the mixture was heated to 90 °C. Upon heating the mixture clarified within 10 min at 90 °C. No formation of gas was observed. The reaction mixture was let cooled down to room temperature and stirred over night. The next day a distillation apparatus and an oil bath were added. The oil bath was set to 120 °C and the milky distillate starting to come over at 78 °C head temperature was directly distilled into a separation funnel where it forms two phases. The distillate is washed twice with saturated NaCl solution and dried with anhydrous sodium sulfate. Yield is 13.92 g (70 °% with regards to formaldehyde).

Odor: Acrylate glue, oily.

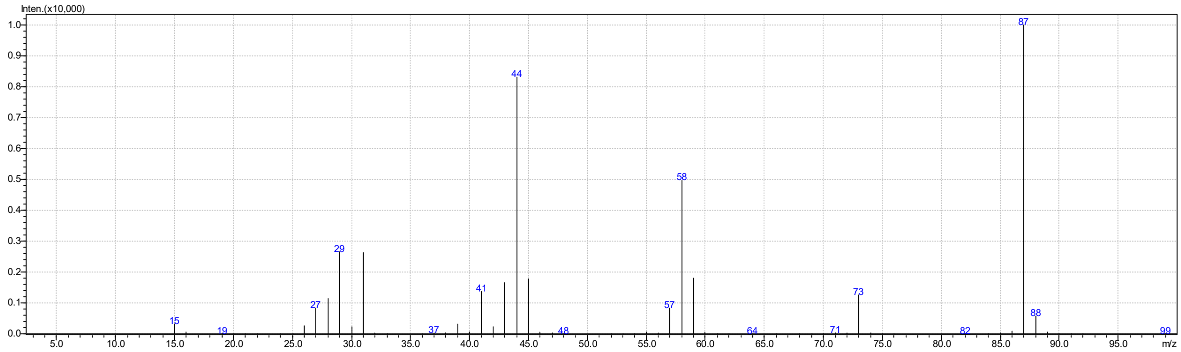

Figure S 7 Mass spectra of 4-Methyl-1,3-dioxolane

2,4-Dimethyl-1,3-Dioxolane (observed)

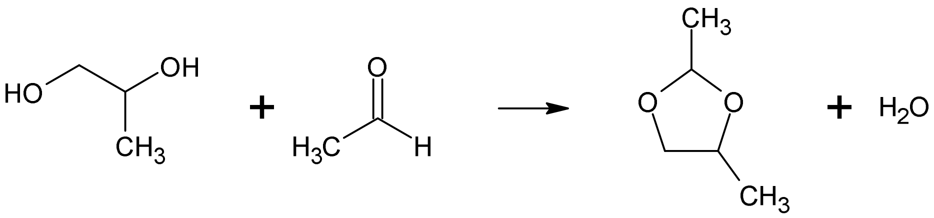

33.73 g of propylene glycol and 9.7 g acetaldehyde were filled into a 100 mL round bottom flask and cooled to 0 °C in an ice bath. One drop of sulfuric acid was added and the mixture was stirred at room temperature over night. The next day the reaction mixture was distilled and at 78 °C a milky distillate came over. The distillate was washed twice with saturated NaCl solution and the organic phase was dried with sodium sulfate. The yield was 12.81 g (57 % with regards to acetaldehyde).

Odor: spray adhesive, fruity, honey.

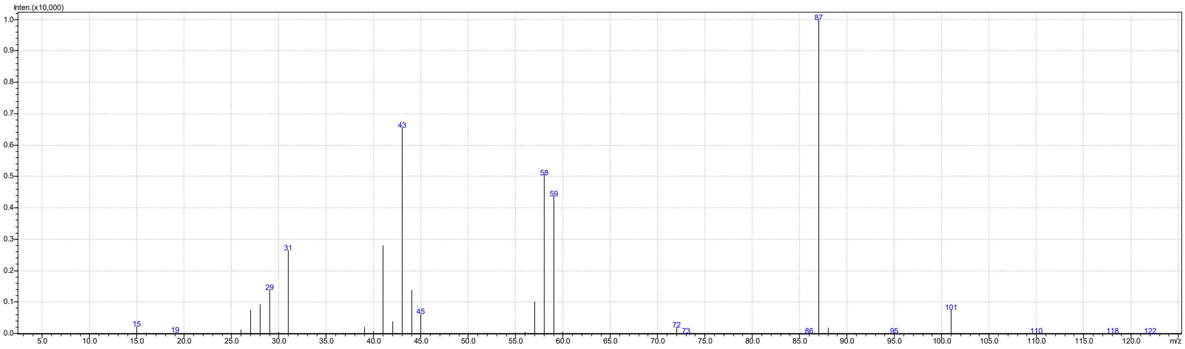

Figure S 8 Mass spectra of 2,4-Dimethyl-1,3-dioxolane

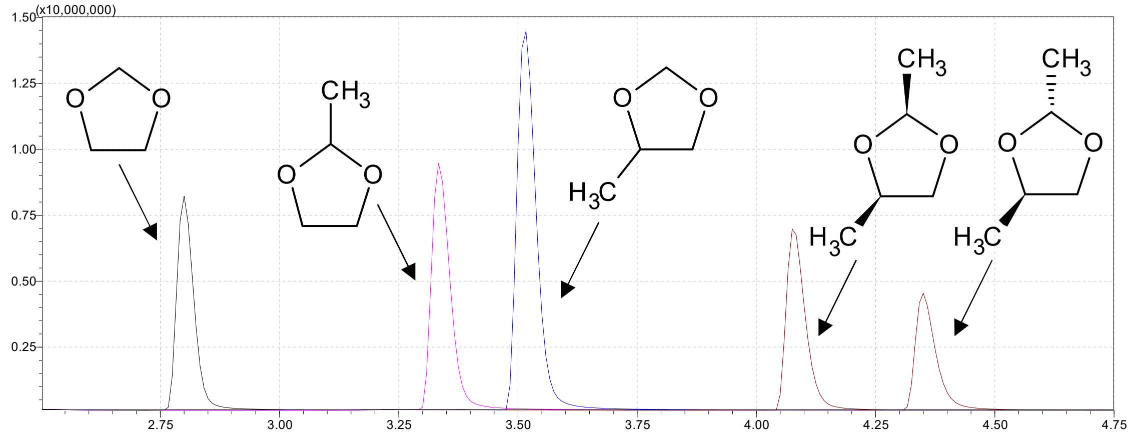

2-Ethyl-1,3-dioxolane

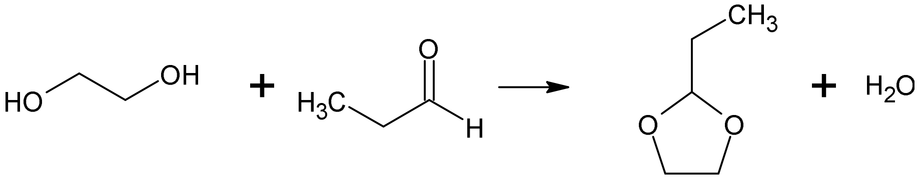

31 g of ethylene glycol (0.5 mol) and 12.3 g of propionic aldehyde (0.25 mol) were filled into a 100 mL round bottom flask. The mixture immediately starts warming up. The reaction mixture is stirred over night at room temperature. The next day it is distilled with an oil bath at 120 °C. A distillate is collected from 72 °C to 100 °C. The two-phase distillate is washed twice with saturated NaCl solution and the organic phase is dried with sodium sulfate. The yield was 6.17 g (24 % with regards to propionic aldehyde).

Odor: sweet, disgusting.

2-Ethyl-4-Methyl-1,3-dioxolane

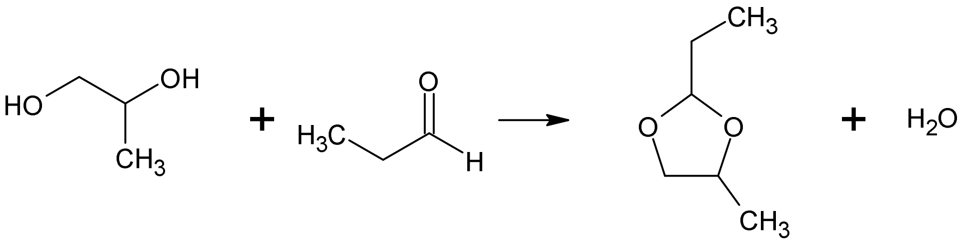

41 g of propylene glycol (0.54 mol) and 16.8 g of propionic aldehyde (0.27 mol) were filled into a 100 mL round bottom flask. The mixture immediately warms up. The reaction mixture is stirred over night at room temperature. The next day it is distilled with an oil bath at 120 °C. First unreacted propionic aldehyde is distilled off at 49 °C. A second fraction is collected from 70 °C to 100 °C. The two-phase distillate is washed twice with saturated NaCl solution and the organic phase is dried with sodium sulfate. The yield was 5.67 g (18 % with regards to propionic aldehyde).

Odor: coating solvent, intense, sharp.

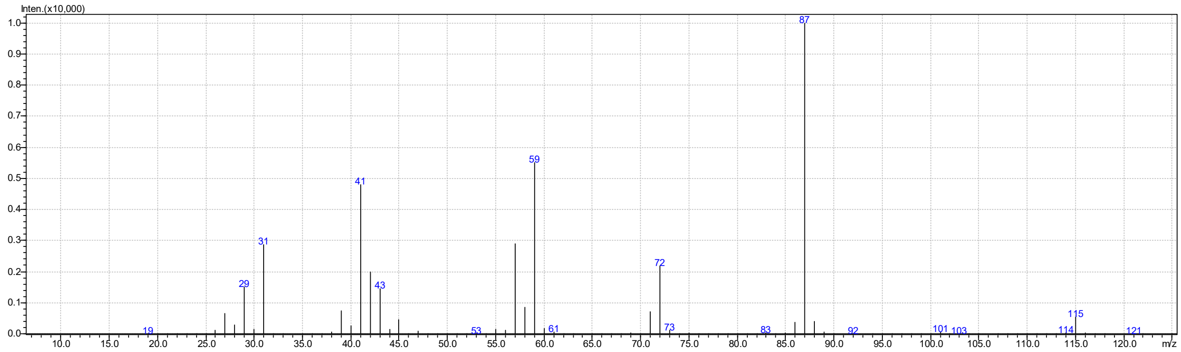

Figure S 9 Mass spectra of 2-Ethyl-4-Methyl-1,3-dioxolane

Synthesis of 2,5-Dimethyl-1,4-dioxane (observed)

In a 100 mL round bottom flask there was added 30 mL of dipropylene glycol isomer mixture and three drops of concentrated sulfuric acid. A distillation apparatus was attached and the solution was heated to 180 °C. A mixture of water, 2,5-dimethyl-1,4-dioxane and 2-ethyl-4-methyl-dioxolane was distilled over at head temperatures between 100 and 140 °C. The distillate separated into two layers.

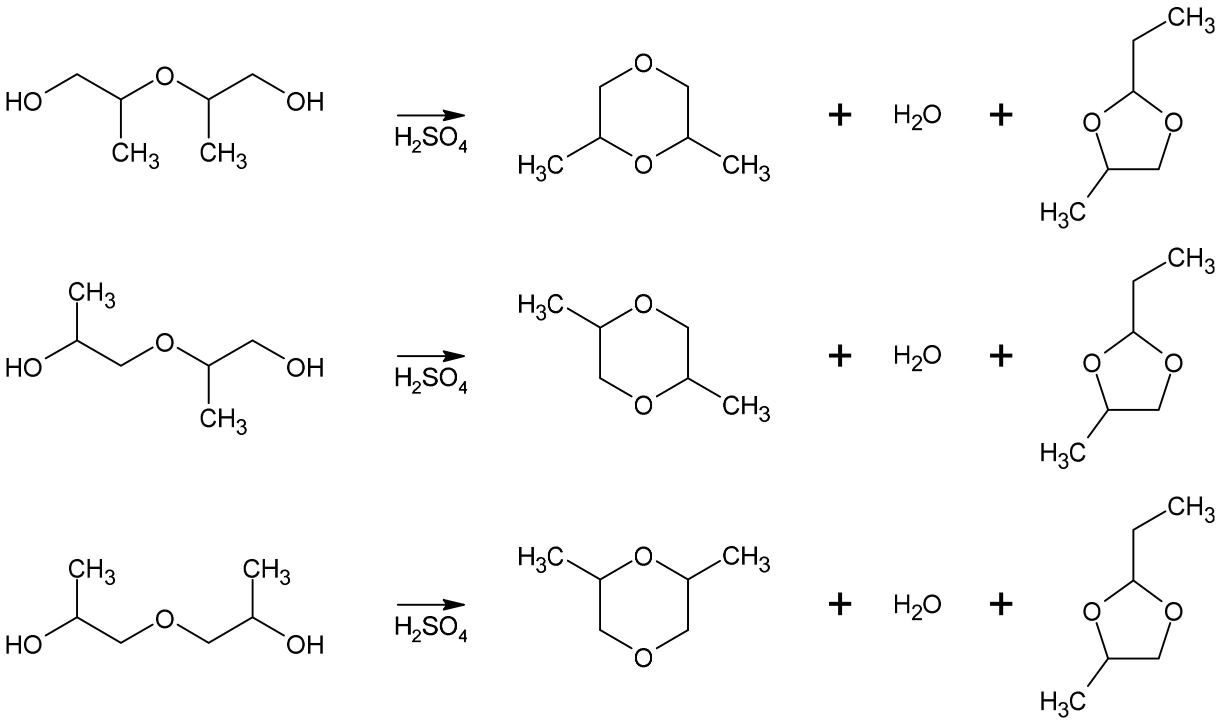

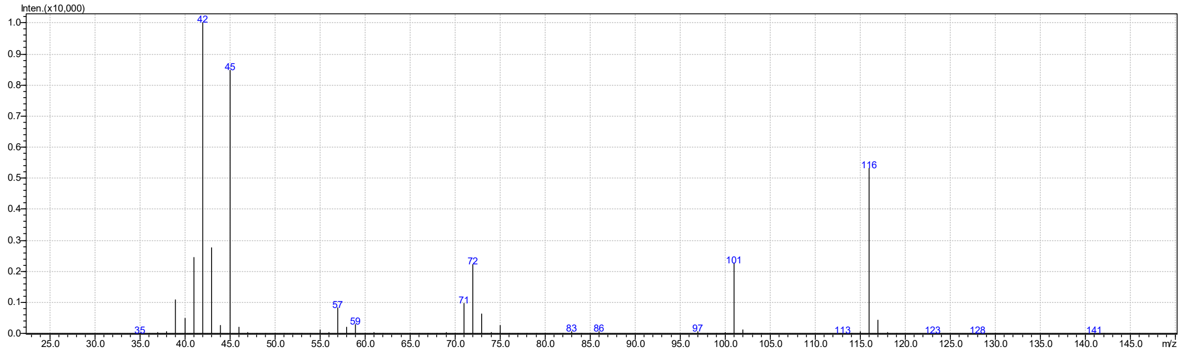

Figure S 10 Mass spectra of 2,5-Dimethyl-1,4-dioxane

1,3,6-Trioxocane

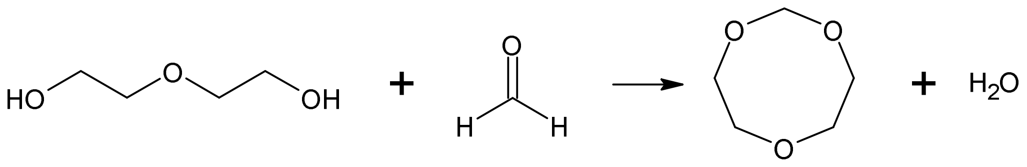

26,86 g of diethyleneglycol (0,25 mol) and 4,46 g paraformaldehyde (0.15 mol) were filled into a 100 mL round bottom flask. The flask was cooled to 0 °C and sulfuric acid was added. Then the mixture was heated to 90 °C to depolymerize the paraformaldehyde. After the mixture clarified it was cooled to room temperature and stirred overnight. The next day the mixture was neutralized with sodium carbonate to avoid the formation of dioxanes during distillation. The distillation was performed with an oil bath at 200 °C. At a head temperature of 87 °C the first oily repulsively smelling drops started to distill over. The distillation is very slow, the reaction mixture turns brown. After distillation no two-phase system is observed and the addition of saturated NaCl solution does not lead to the formation of two-phases. The distillate is washed twice with diethylether. The organic phases were combined, dried and the ether was removed. The residue was extremely small. A solution in chloroform was created to generate a mass spectrum. No yield was calculated.

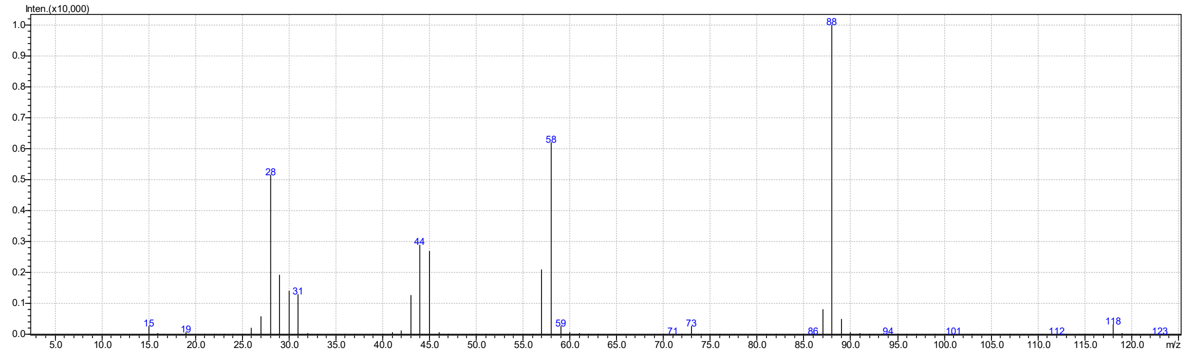

Figure S 11 Mass spectra of 1,3,6-Trioxocane

2-Methyl-1,3,6-Trioxocane

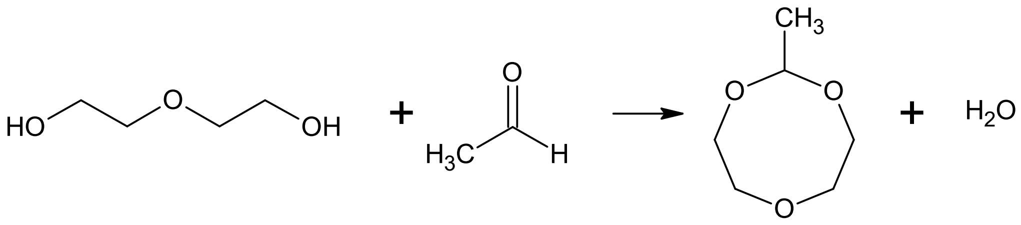

30 g of diethyleneglycol (0,28 mol) and 6.27 g paraformaldehyde (0.14 mol) were filled into a 100 mL round bottom flask. The flask was cooled to 0 °C and sulfuric acid was added. The mixture was kept at room temperature and stirred overnight. The next day the mixture was neutralized with sodium carbonate to avoid the formation of dioxanes during distillation. The distillation was performed with an oil bath at 200 °C. After non-reacted acetaldehyde is distilled, at a head temperature of 82 °C a minor amount of a yellow liquid is distilled over. A solution in chloroform was created to generate a mass spectrum. No yield was calculated.

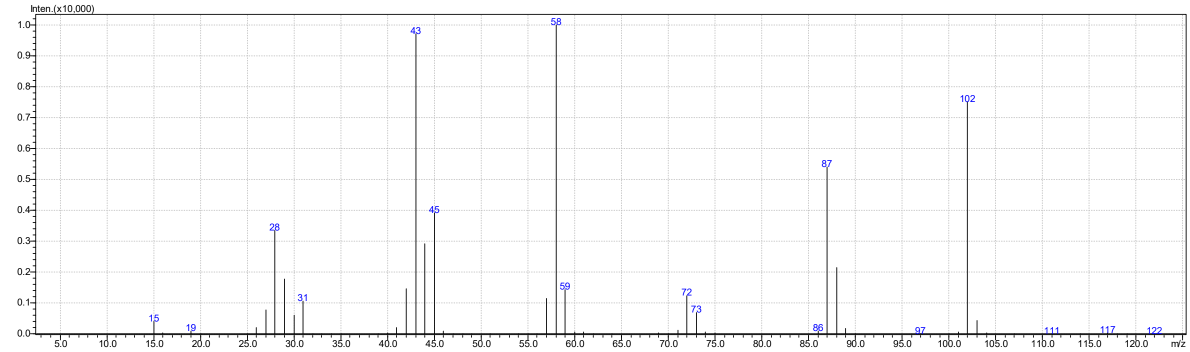

Figure S 12 Mass spectra of 2-Methyl-1,3-6-trioxocane

Dimethyltrioxocane isomer mixture synthesis

22.5 g of dipropylene glycol isomer mixture (0.17 mol) and 3.2 g paraformaldehyde (0.08 mol) were filled into a 100 mL round bottom flask. The flask was cooled to 0 °C and sulfuric acid was added. The mixture was kept at room temperature and stirred overnight. The next day the mixture was neutralized with sodium carbonate to avoid the formation of dioxanes during distillation. The distillation was performed with an oil bath at 200 °C and at a pressure of 5 mbar. A small amount of liquid distilled over at 168 °C. A solution in chloroform was created to generate a mass spectrum. No yield was calculated.

Odor: rancid glue.

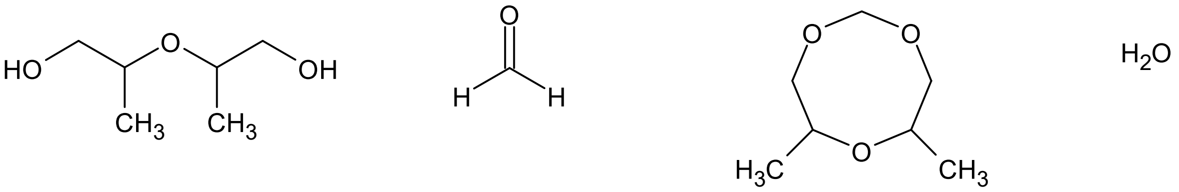

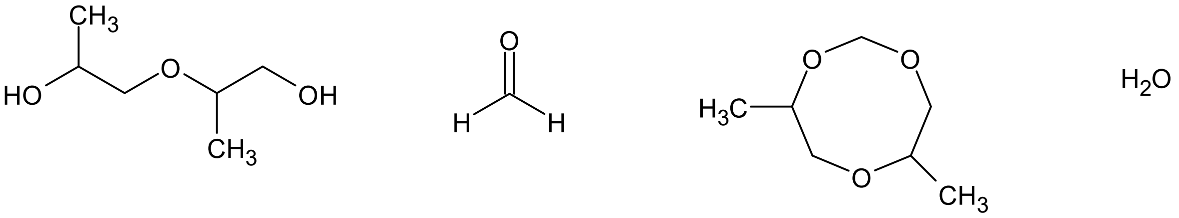

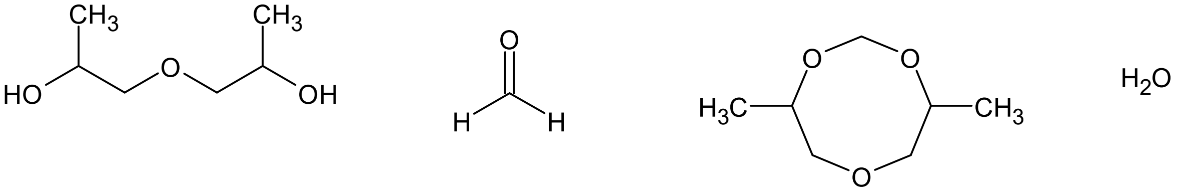

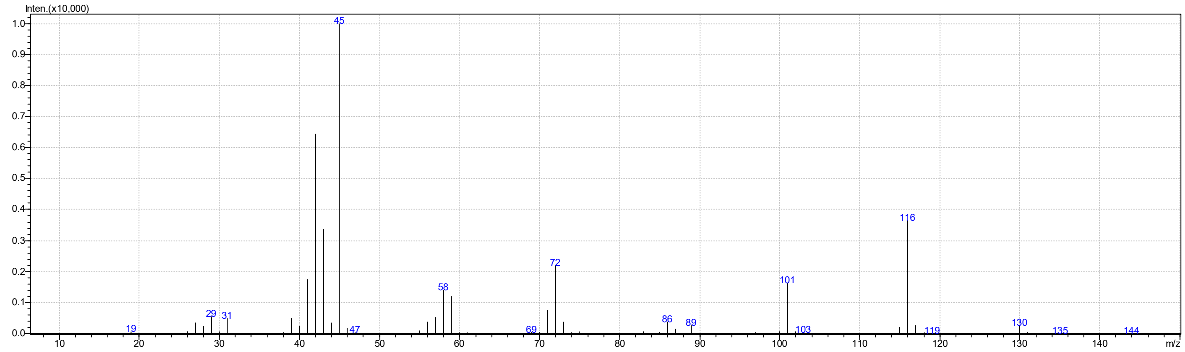

Figure S 13 Mass spectra of Dimethyl-1,3,6-Trioxocane isomeric mixture

Trimethyltrioxocane isomer mixture synthesis

26.5 g of dipropylene glycol isomer mixture (0.19 mol) and 6.5 g acetaldehyde (0.1 mol) were filled into a 100 mL round bottom flask. The flask was cooled to 0 °C and sulfuric acid was added. The mixture was kept at room temperature and stirred overnight. The next day the mixture was neutralized with sodium carbonate to avoid the formation of dioxanes during distillation. The distillation was performed with an oil bath at 200 °C and at a pressure of 5 mbar. A small amount of liquid distilled over at 152 °C. A solution in chloroform was created to generate a mass spectrum. No yield was calculated.

Odor: watercress, toilet cleaner.

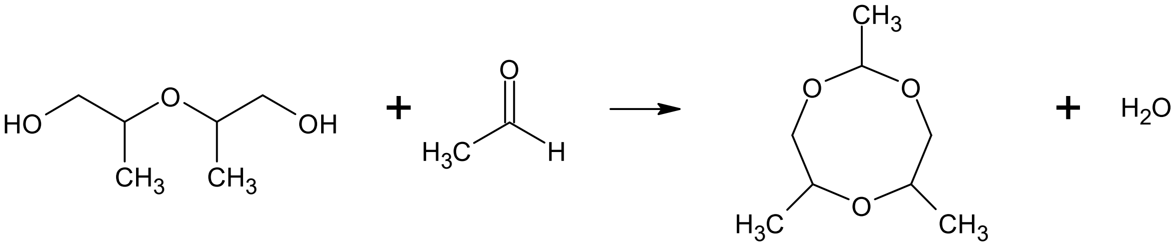

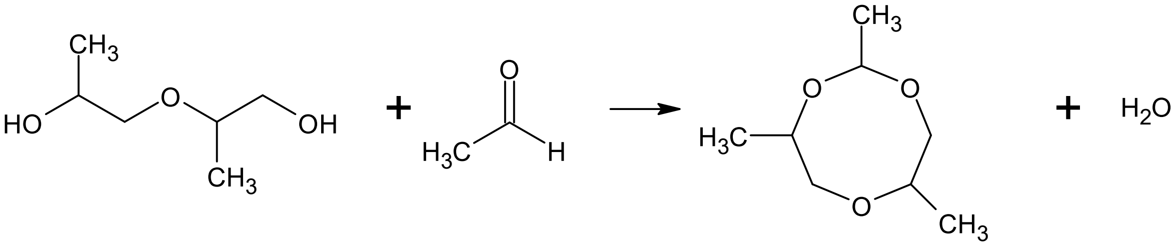

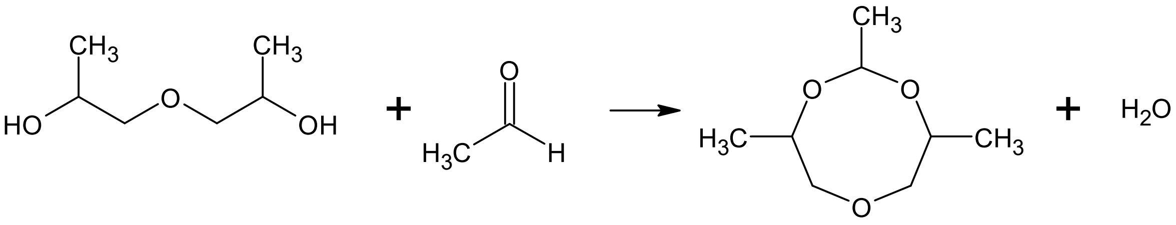

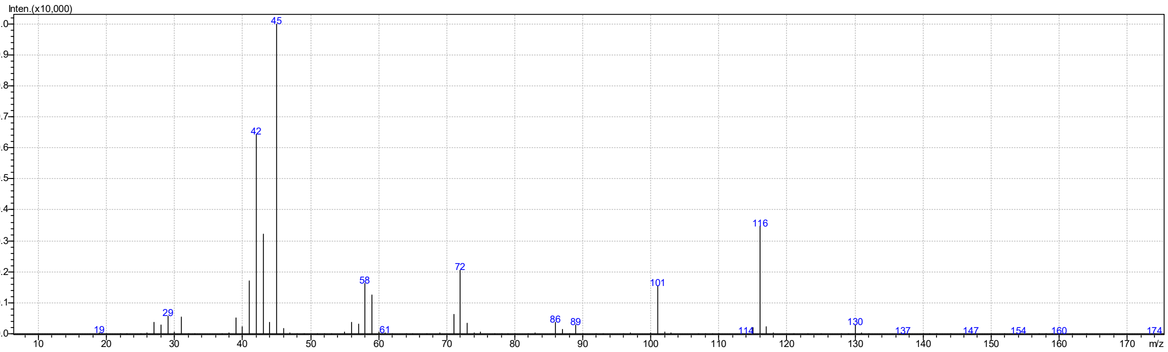

Figure S 14 Mass spectra of Trimethyl-1,3,6-Trioxocane isomeric mixture

Ethyldimethyltrioxocane isomer mixture synthesis

44.2 g of dipropylene glycol isomer mixture (0.32 mol) and 9.22 g of propionic aldehyde (0.16 mol) were filled into a 100 mL round bottom flask. The flask was cooled to 0 °C and sulfuric acid was added. The mixture was kept at room temperature and stirred overnight. The next day the mixture was neutralized with sodium carbonate to avoid the formation of dioxanes during distillation. The distillation was performed with an oil bath at 200 °C and at a pressure of 5 mbar. A small amount of liquid distilled over at 145 °C. A solution in chloroform was created to generate a mass spectrum. No yield was calculated.

Odor: sweet, ethereal, musty.

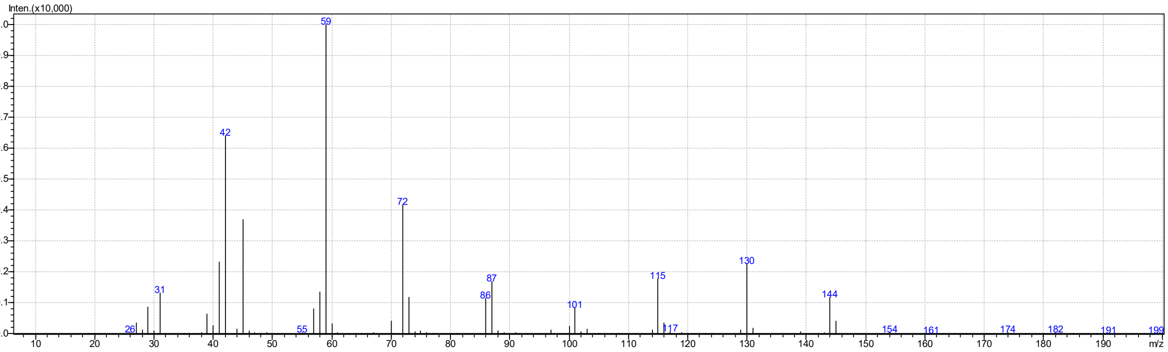

Figure S 15 Mass spectra of 2-Ethyl-4,7-dimethyl-1,3,6-trioxocane

Aldehyde synthesis

Diethyleneglycol dialdehyde (observed)

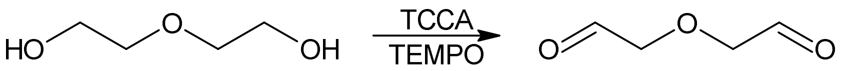

To an ice-cooled solution of 2 g diethylene glycol (19 mmol) in 150 mL dichloromethane, some crystals of TEMPO were added. To this solution, 8.77 g trichloroisocyanuric acid (38 mmol) was added. After stirring for 45 minutes, the reaction mixture was filtered through a preparative chromatographic column with a 2 cm layer of celite packing. The organic phase was shaken three times with 10 mL of saturated sodium bicarbonate solution. The solution decolorized, and gas evolution was observed in the separating funnel. The organic phase was then dried with sodium sulfate and concentrated on a rotary evaporator at 50 °C. The residue was an amber-colored, low-viscosity liquid. Mass 1.8 g, yield 94%.

Odor: unpleasant

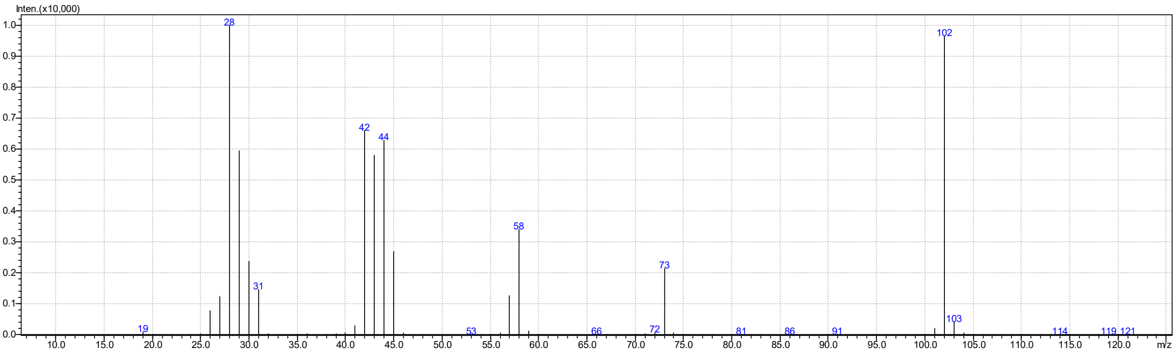

Figure S 16 Mass spectra of Diethyleneglycoldialdehyde

Synthesis of (dipropylene glycol aldehyde ketone) (observed)

To an ice-cooled solution of 2 g dipropylene glycol isomer mixture (14.9 mmol) in 150 mL dichloromethane, some crystals of TEMPO were added. To this solution, 6.93 g trichloroisocyanuric acid (29.8 mmol) was added. After stirring for 45 minutes, the reaction mixture was filtered through a preparative chromatographic column with a 2 cm layer of celite packing. The organic phase was shaken three times with 10 mL of saturated sodium bicarbonate solution. The solution decolorized, and gas evolution was observed in the separating funnel. The organic phase was then dried with sodium sulfate and concentrated on a rotary evaporator at 50 °C. The residue was an amber-colored, low-viscosity liquid. Mass 1.78 g, yield 92%. No pure product could be isolated, due to the formation of the bis aldehyde and the bis ketone.

Odor: pleasant, fruity, whisky-like

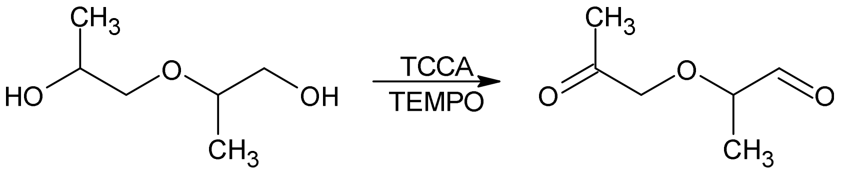

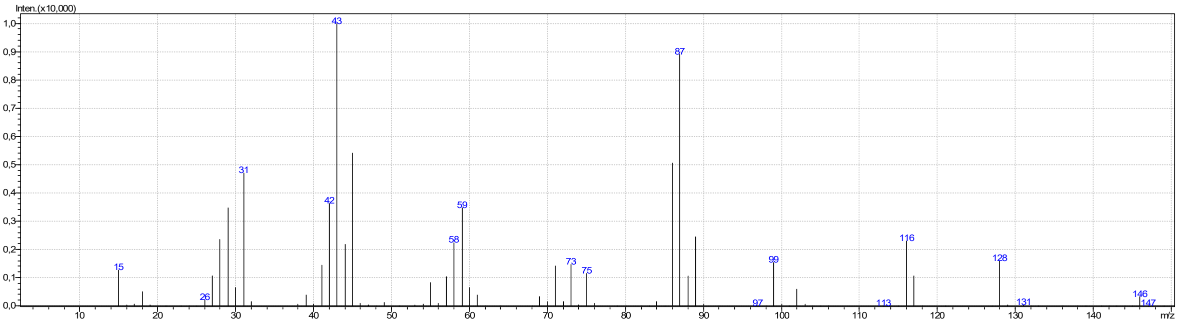

Figure S 17 Mass spectra pf dipropylene glycol aldehyde ketone

In-situ generated reference materials

Mass spectra of polyalkylenoxide products

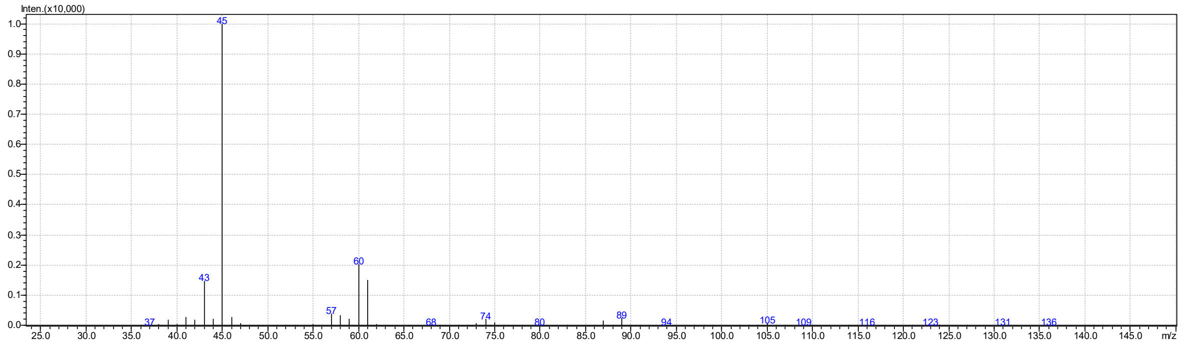

Figure S 18 Mass spectra of 1,2-Propanediol-1-Formate (observed)

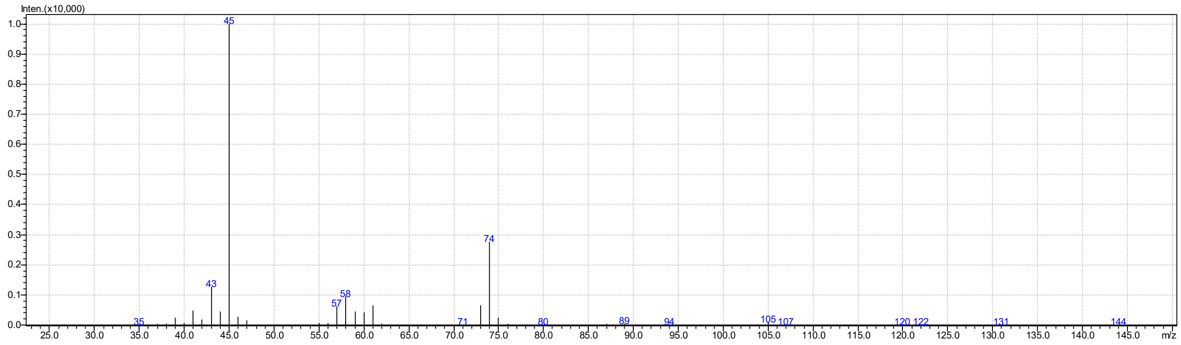

Figure S 19 Mass spectra of 1,2-Propandiol-2-Formate (observed)

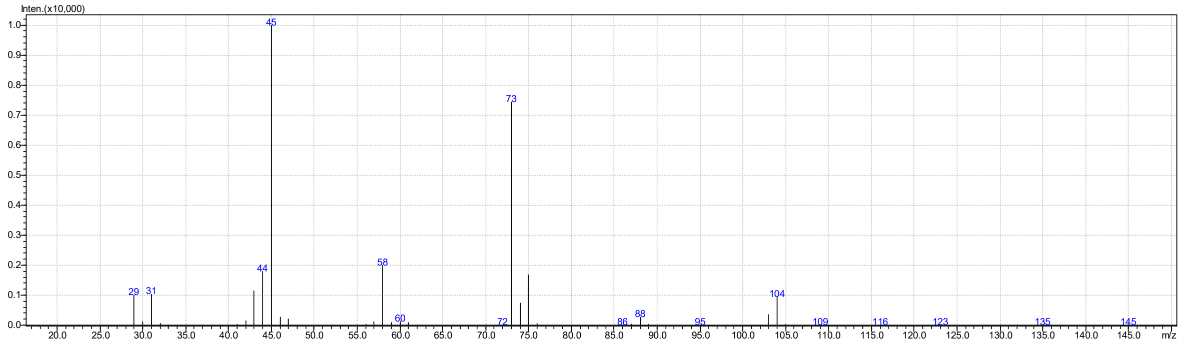

Figure S 20 Mass spectra of Diethyleneglycolmonoformate

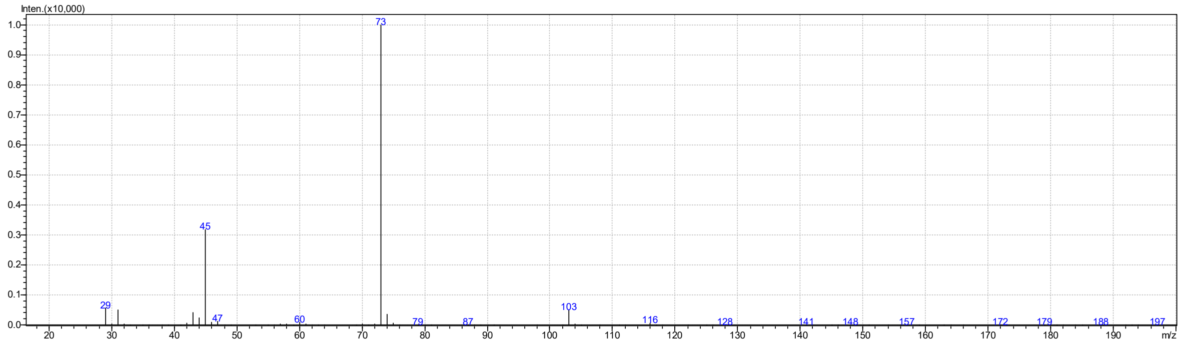

Figure S 21 Mass spectra of Diethyleneglycoldiformate

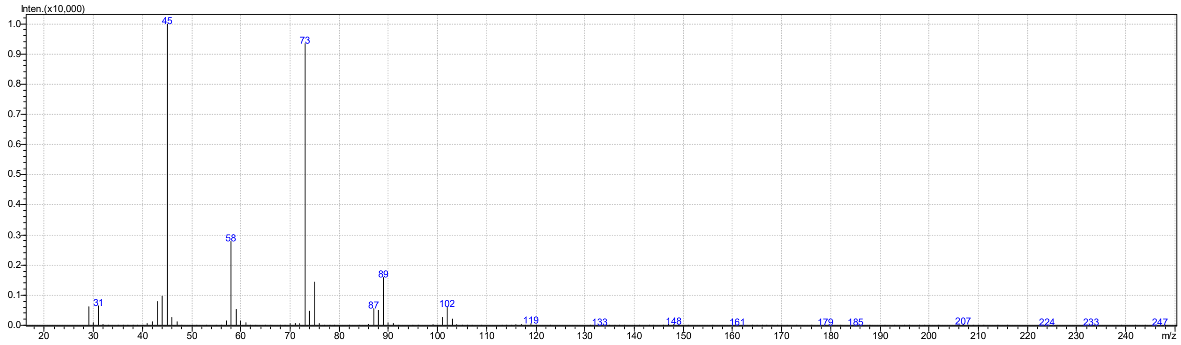

Figure S 22 Mass spectra of Triethyleneglycolmonoformate

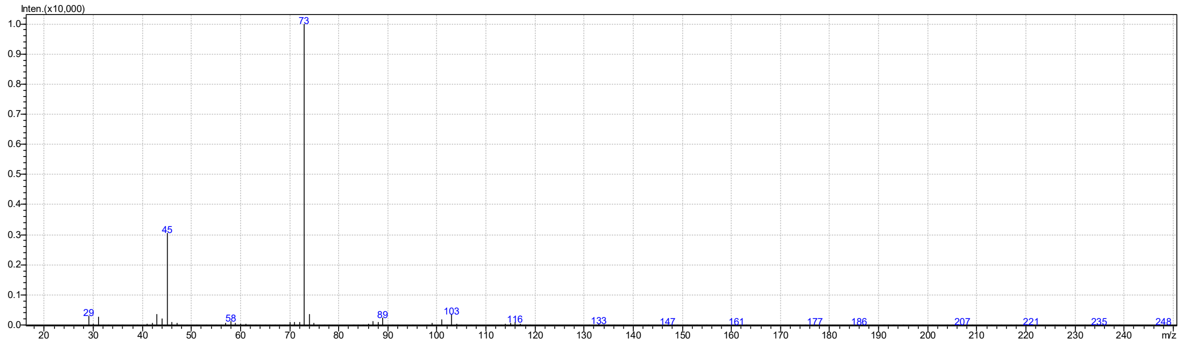

Figure S 23 Mass spectra of Triethyleneglycoldiformate

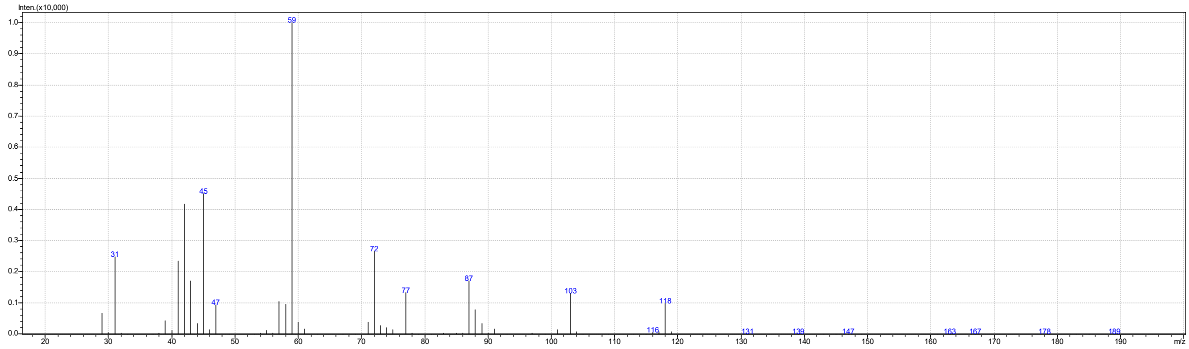

Figure S 24 Mass spectra of Dipropyleneglycolmonoformate isomeric mixture

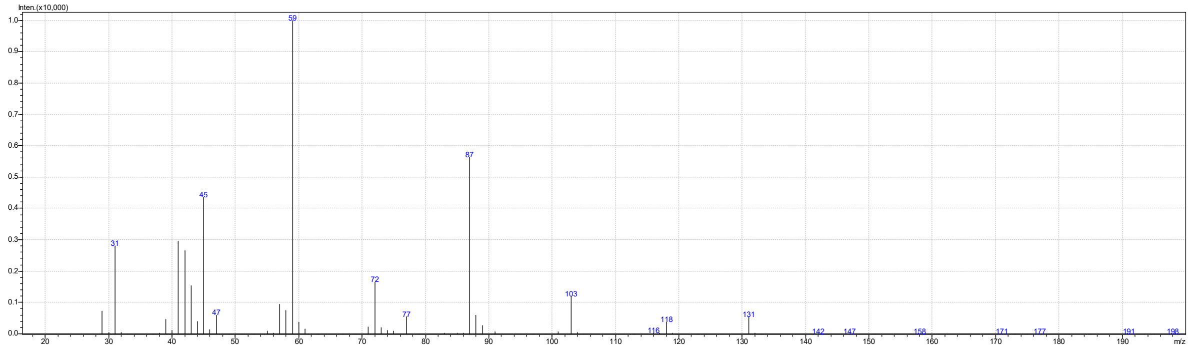

Figure S 25 Mass spectra of Dipropyleneglycolmonoformate isomer 1

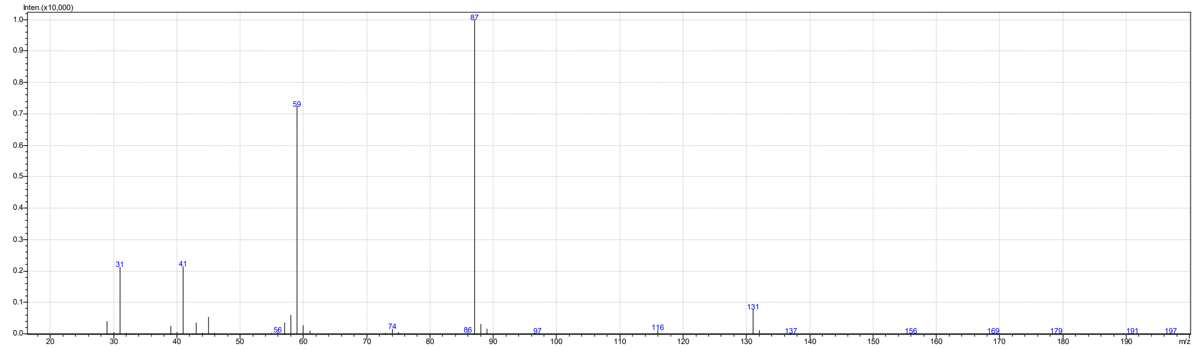

Figure S 26 Mass spectra of Dipropyleneglycolmonoformate isomer 2 (observed)

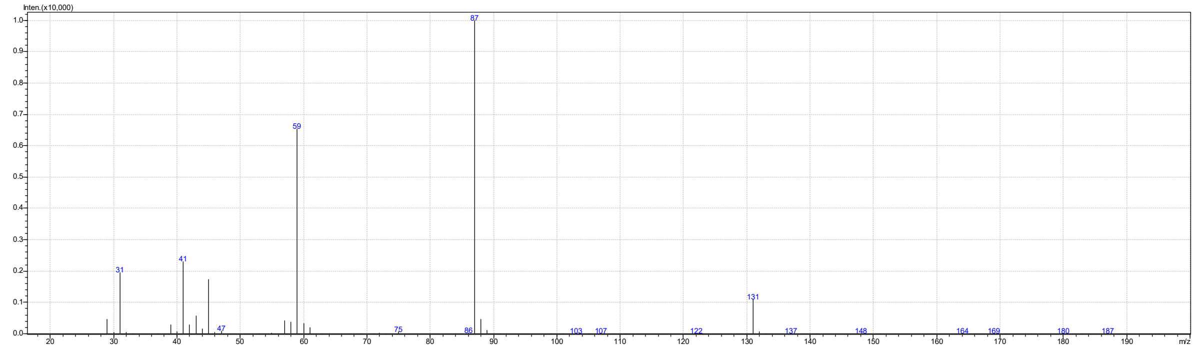

Figure S 27 Mass spectra of Dipropyleneglycoldiformate isomer 1

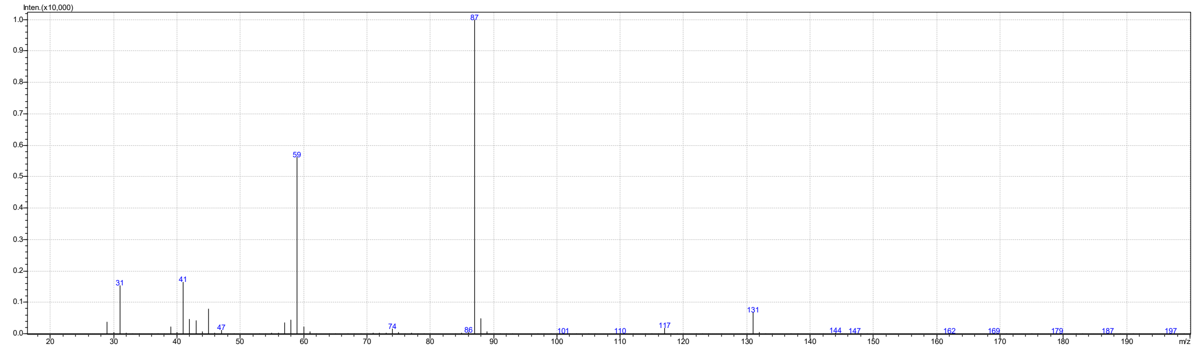

Figure S 28 Mass spectra of Dipropyleneglycoldiformate isomer 2

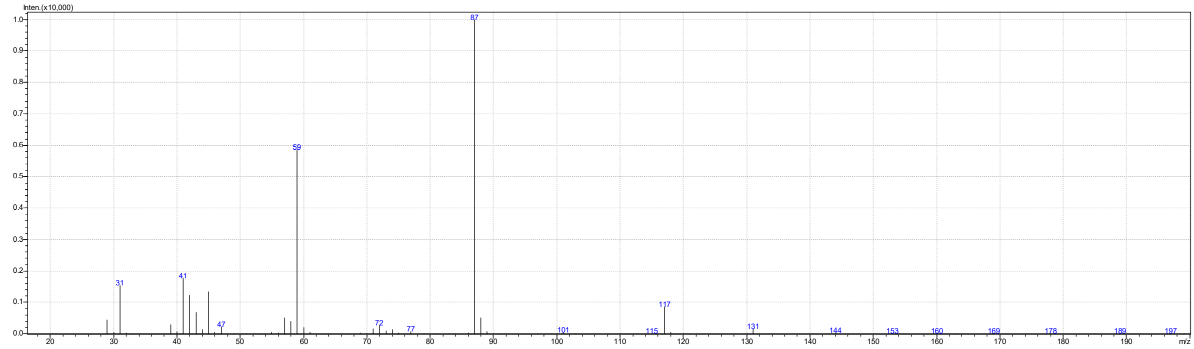

Figure S 29 Mass spectra of Dipropyleneglycoldiformate isomer 3

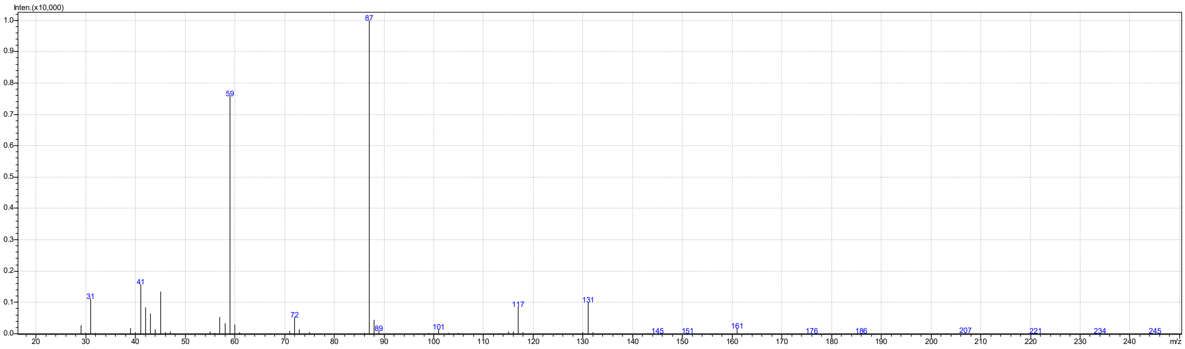

Figure S 30 Mass spectra of tripropyleneglycolmonoformate isomer 1

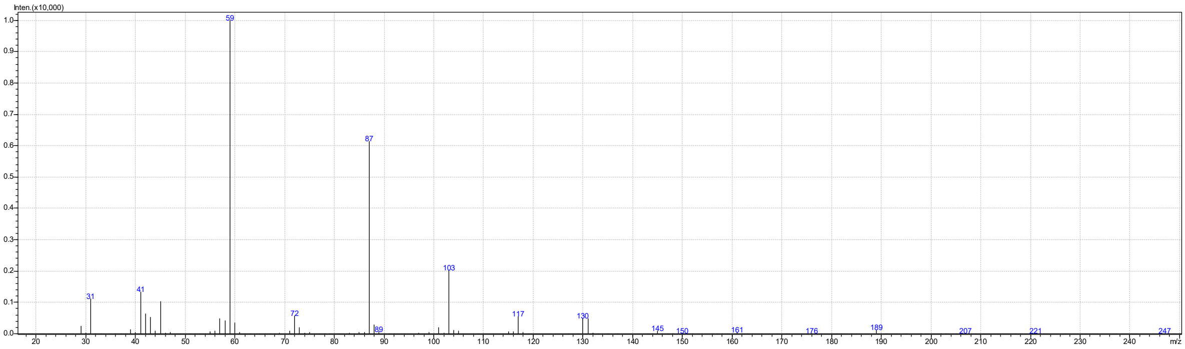

Figure S 31 Mass spectra of tripropyleneglycolmonoformate isomer 2

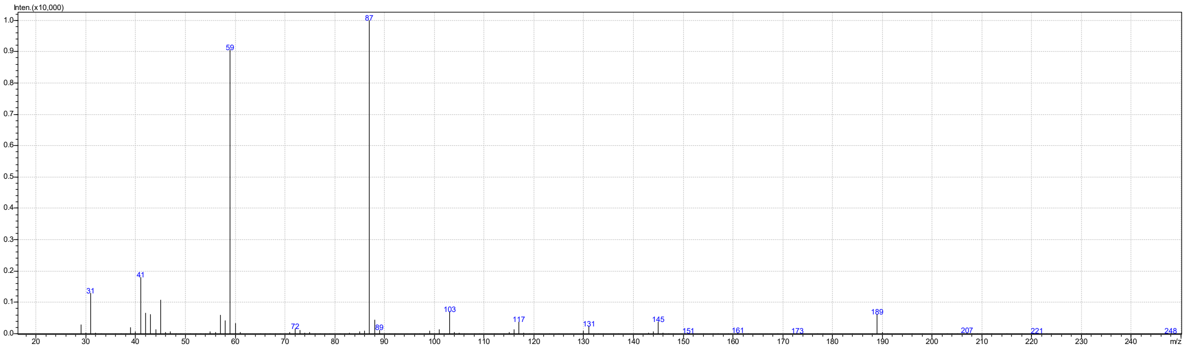

Figure S 32 Mass spectra of tripropyleneglycolmonoformate isomer 3

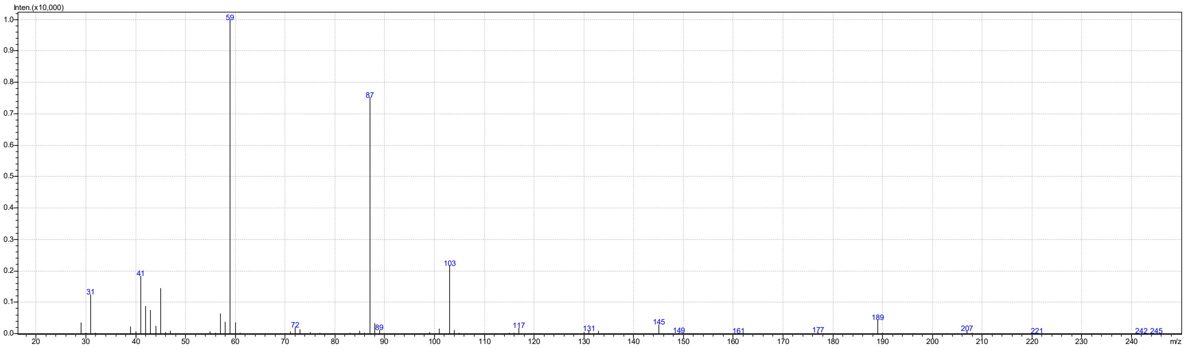

Figure S 33 Mass spectra of tripropyleneglycolmonoformate isomer 4

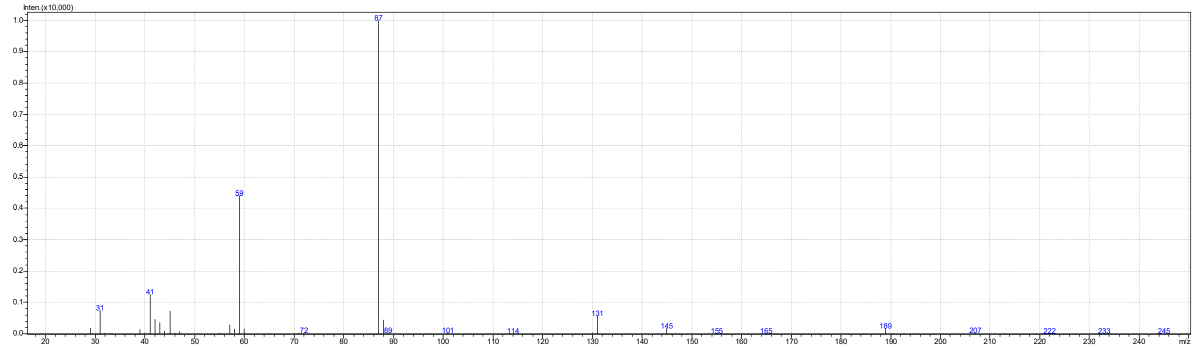

Figure S 34 Mass spectra of tripropyleneglycoldiformate isomer 1

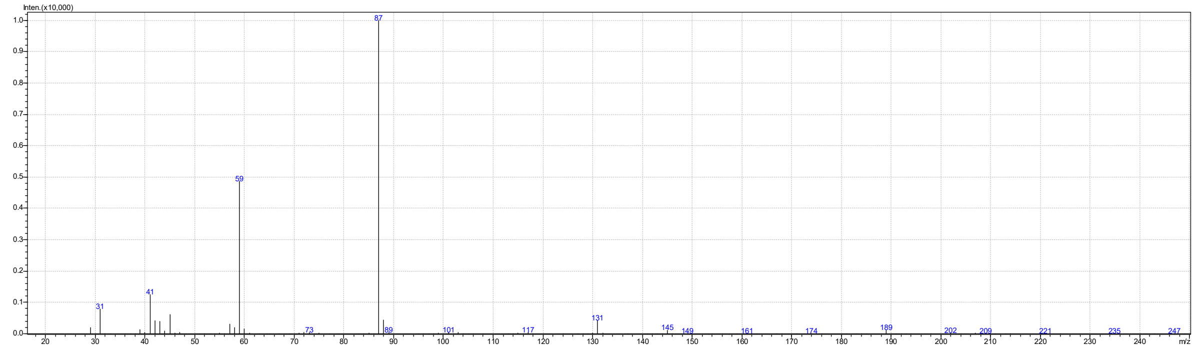

Figure S 35 Mass spectra of tripropyleneglycoldiformate isomer 2

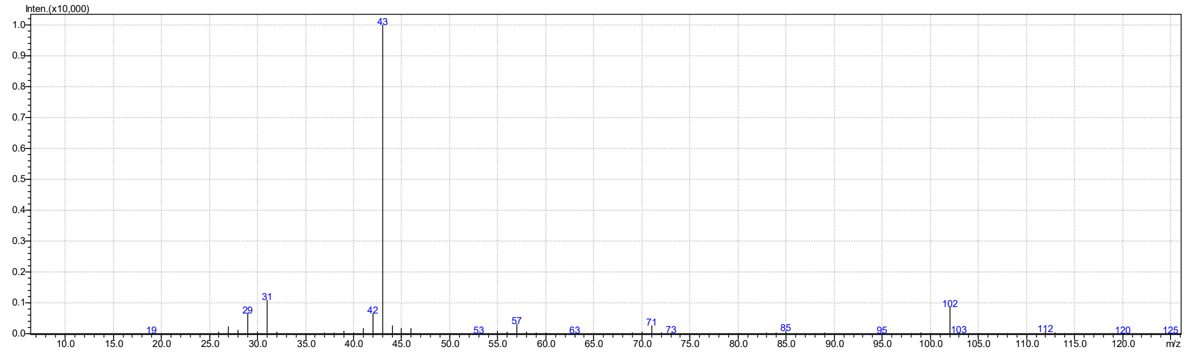

Figure S 36 Hydroxyacetoneformate (observed)

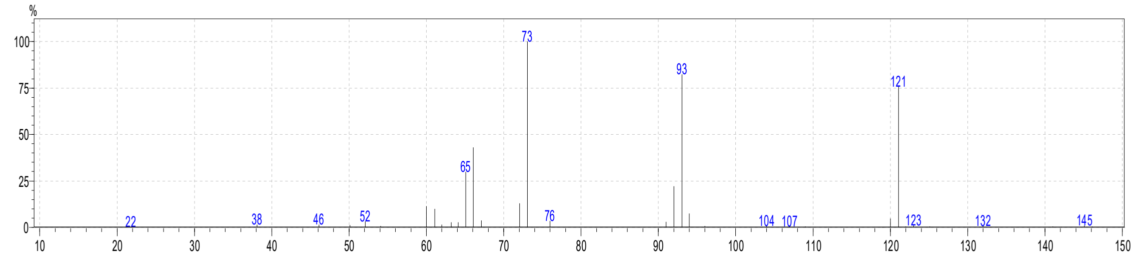

Figure S 37 Mass spectrum of unidentified product of hard-segment oxidation (RT 39 min)
