# Supplementary material for: The Thermo-Oxidative Degradation of Polyurethane Open-Cell Soft Foam Investigated Through Gas Chromatography and Mass Spectrometry of Volatile Organic Compounds
Source: Polymers (Basel). 2024 Nov 28;16(23):3342. doi: 10.3390/polym16233342 (PMC11644683; doi:10.3390/polym16233342)
Supplement: Supplementary file 1 [file polymers-16-03342-s001.zip › Supplemental Material II - Calibration data.pdf]

The Thermo-Oxidative Degradation of Polyurethane Open-Cell Soft Foam Investigated Through Gas Chromatography and Mass Spectrometry of Volatile Organic Compounds

Authors:

Christian Stefan Sandten M.Sc.[a,*] C.Sandten@FH-Muenster.de
Prof. Dr. Martin Kreyenschmidt[a] Martin.Kreyenschmidt@FH-Muenster.de
Dr. Rolf Albach[b] Rolf.Albach@Covestro.com
Prof. Dr. Ursula E. A. Fittschen[c] Ursula.Fittschen@TU-Clausthal.de

a: University of Applied Sciences Muenster Hüfferstraße 27, 48149 Münster, Germany
b: Covestro Deutschland AG Kaiser-Wilhelm-Allee 60, 51373 Leverkusen, Germany
c: Clausthal University of Technology Adolph-Roemer-Straße 2A, 38678 Clausthal-Zellerfeld, Germany

*Corresponding author (Phone: +49 02551 962291)

Supplemental II – Calibration functions

All calibration curves utilized in this study are presented here. The target ion, retention time (min), slope, and intercept are provided for each curve. Additionally, the coefficient of determination (R^2) is included. An estimation of the limit of detection (LOD) and limit of quantification (LOQ) is also provided. However, no samples were analyzed to determine the actual LOD and LOQ; hence, the actual values are likely significantly lower than the calculated estimates.

LOD and LOQ were calculated as:

LOD = 3.3 standard deviation (residuals) / slope

LOQ = 10 standard deviation (residuals) / slope

All quantified sample measurements were within the calibrated range.

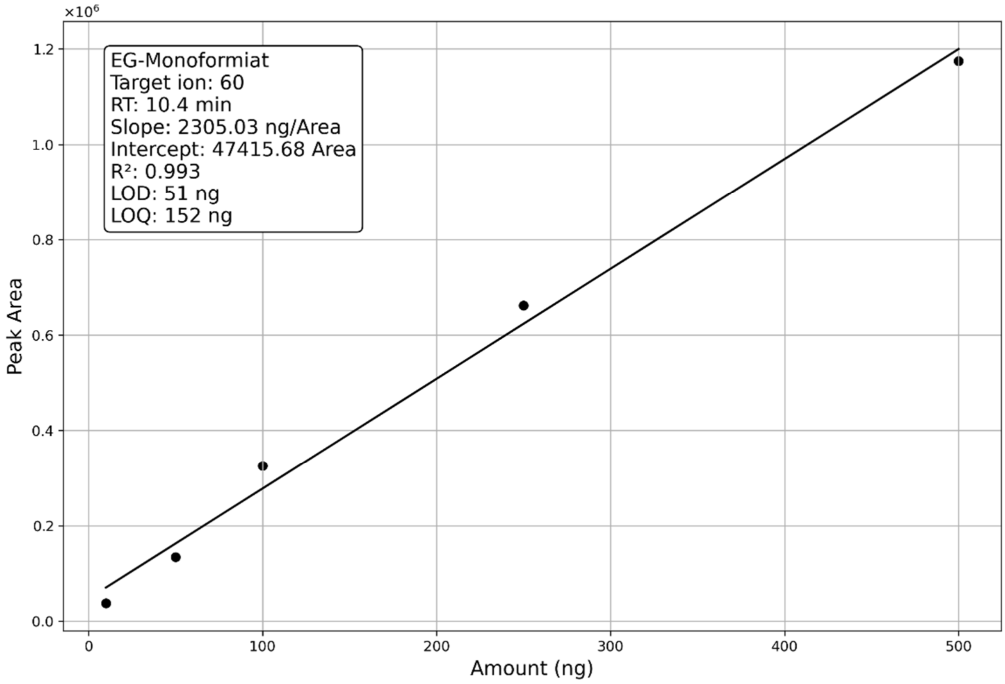

Figure S 1 Calibration curve of 1,2-ethylenediol-monoformate

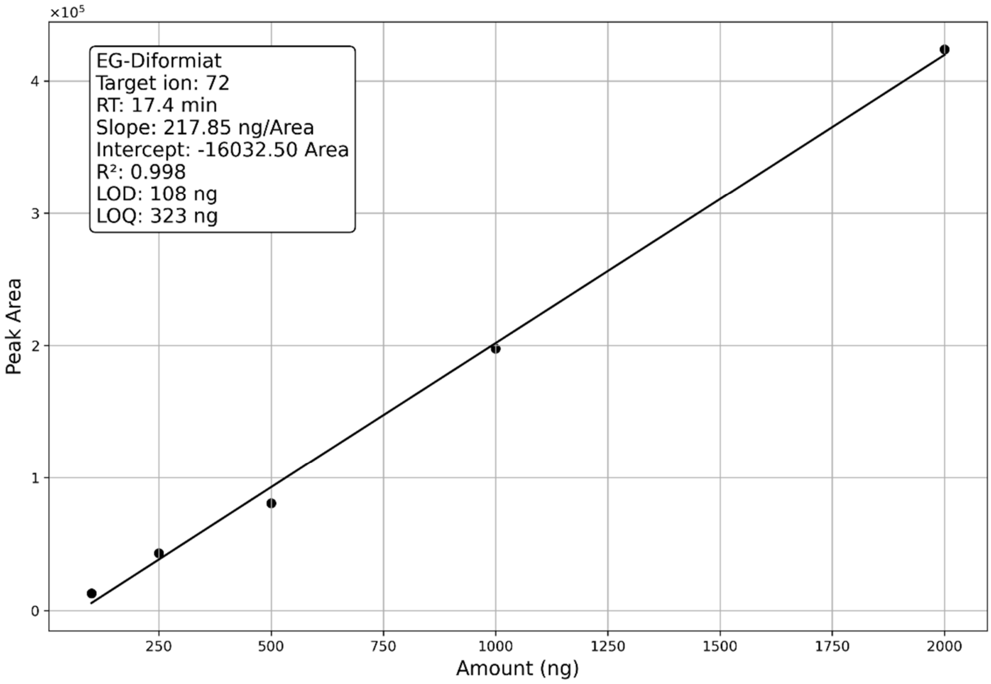

Figure S 2 Calibration curve of 1,2-ethylenediol-diformate

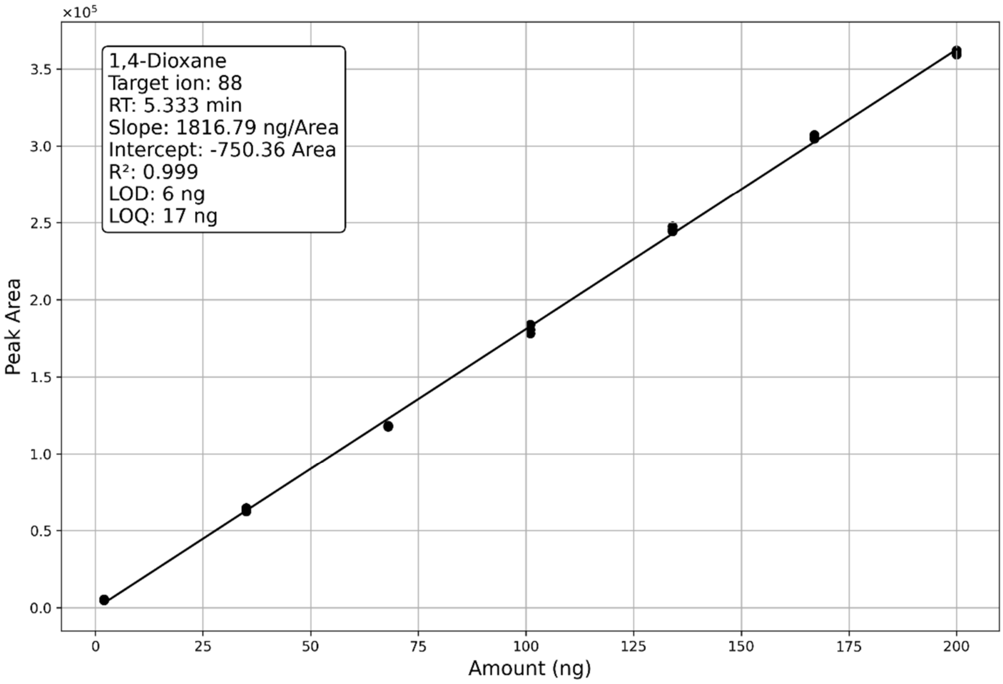

Figure S 3 Calibration curve of 1,4-dioxane

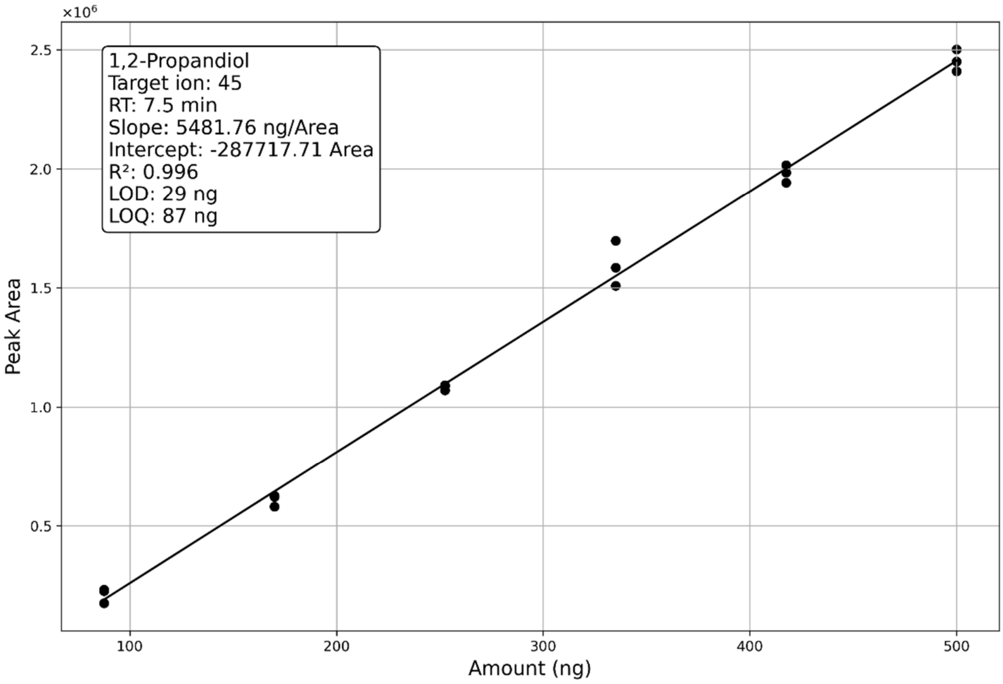

Figure S 4 Calibration curve of 1,2-propanediol

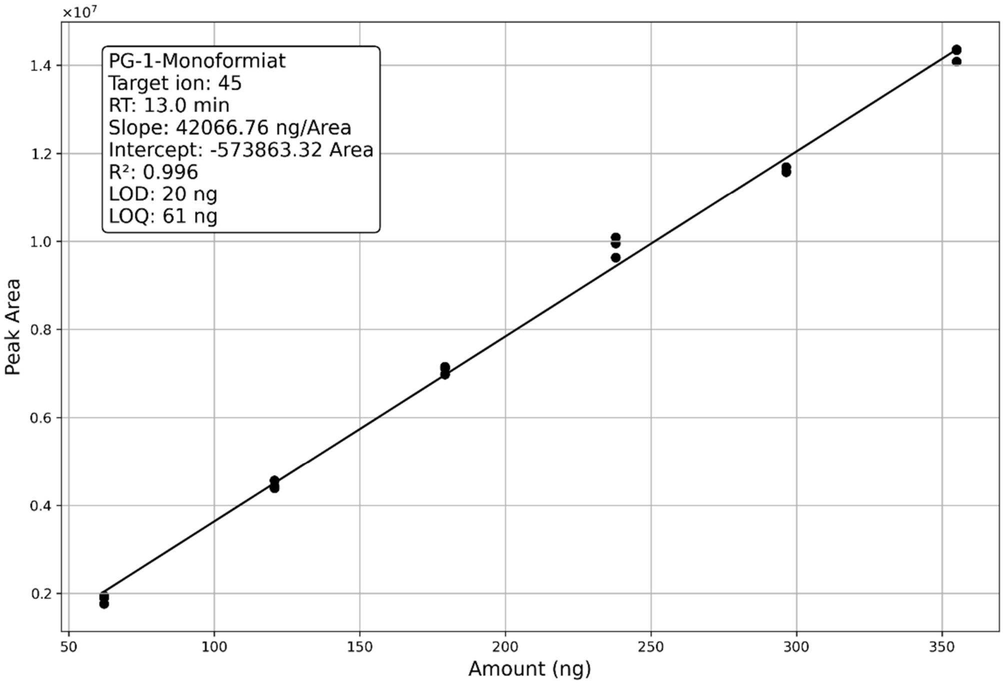

Figure S 5 Calibration curve of 1,2-propanediol-1-monoformate

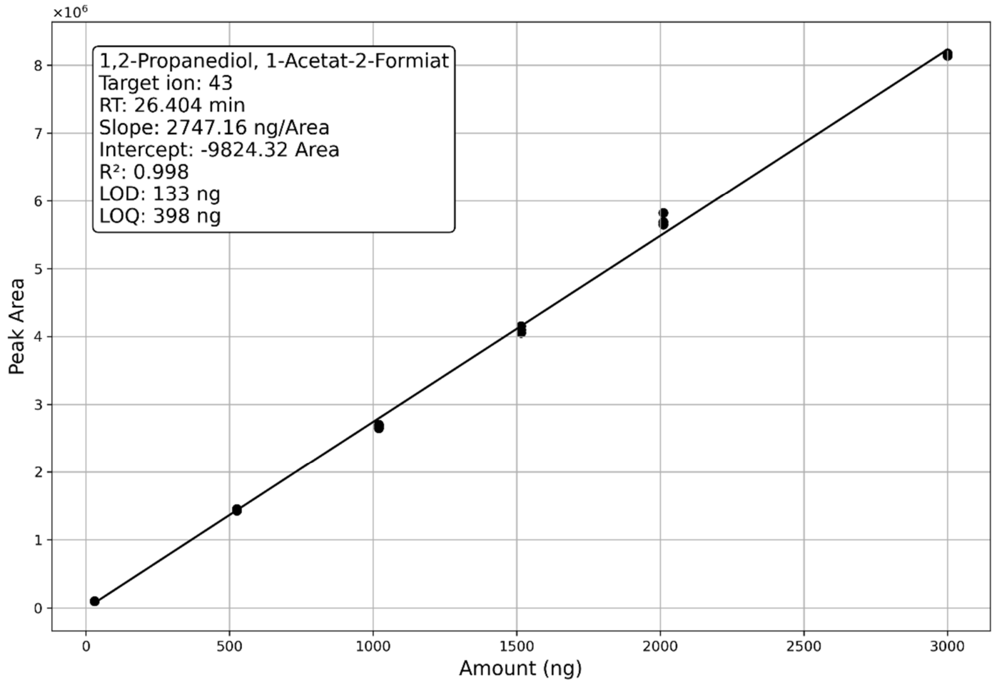

Figure S 6 Calibration curve of 1,2-propanediol-1-acetate-2-formate

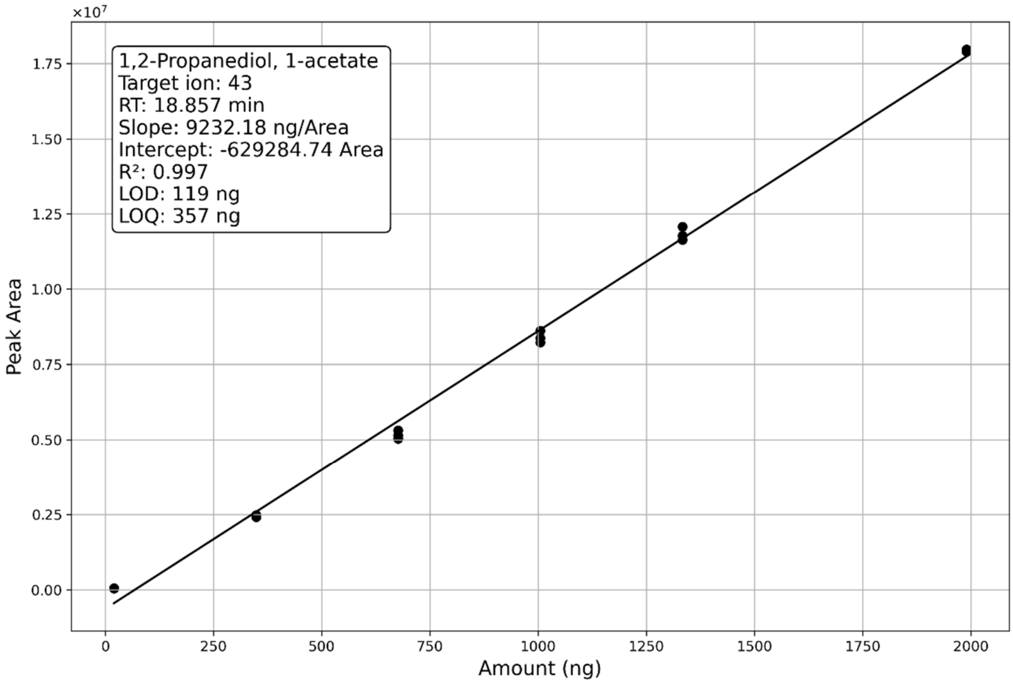

Figure S 7 Calibration curve of 1,2-propanediol-1-acetate

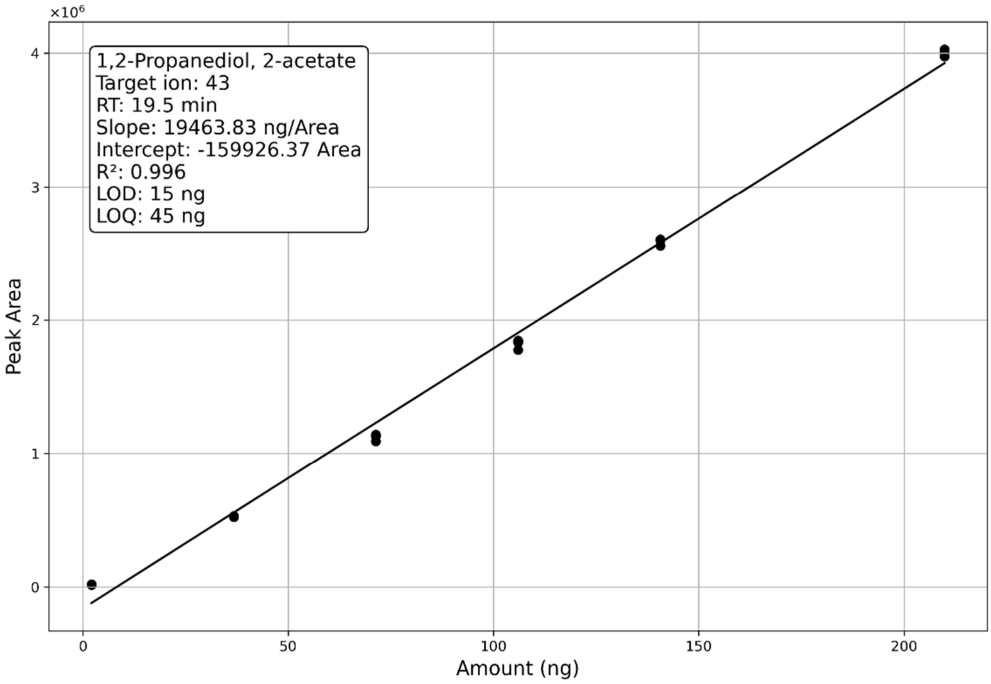

Figure S 8 Calibration curve of 1,2-propanediol-2-acetate

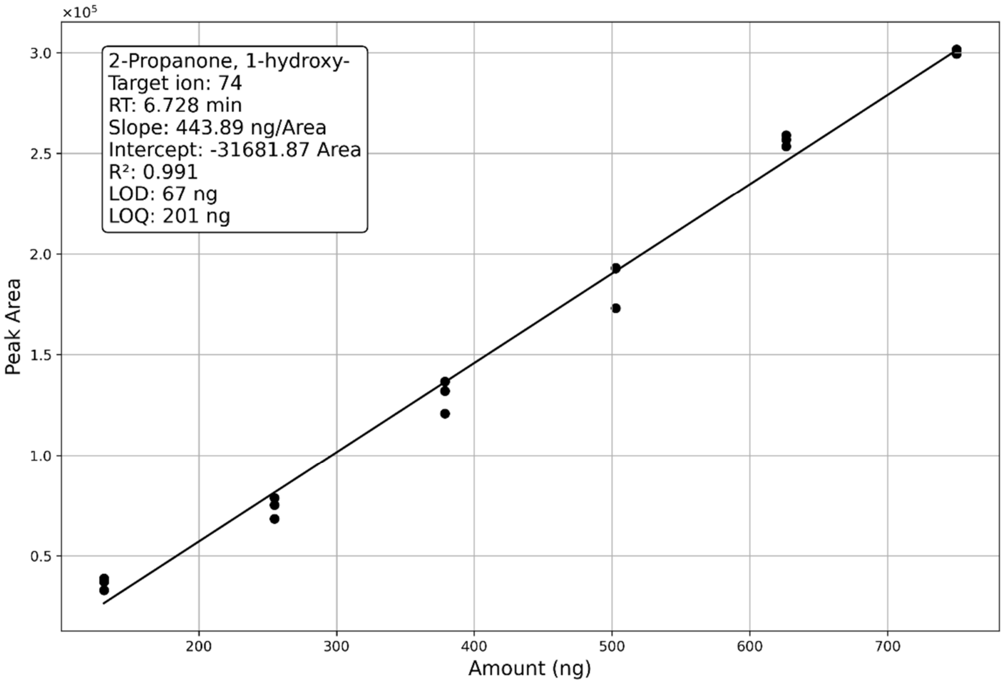

Figure S 9 Calibration curve of 1-hydroxy-2-propanone

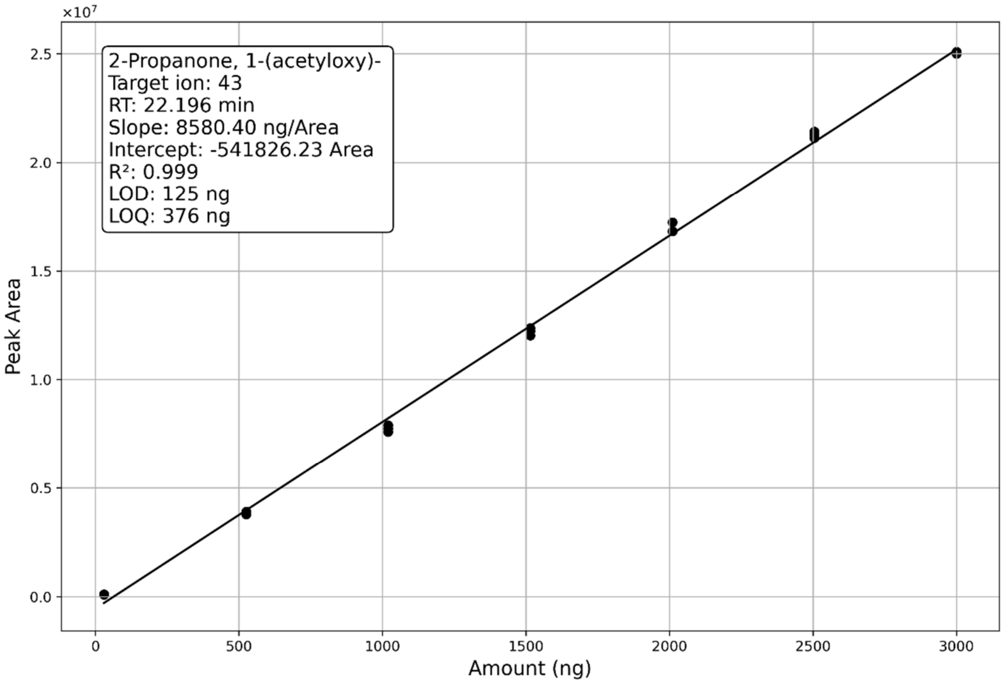

Figure S 10 Calibration curve of hydroxyacetoneacetate / 1-(acetyloxy)-2-propanone

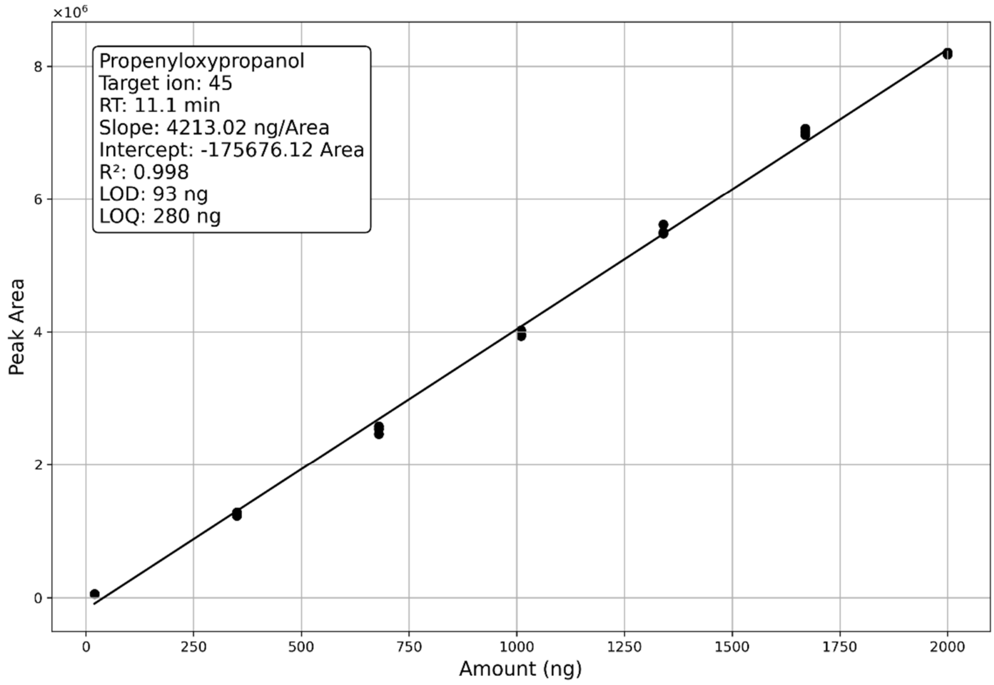

Figure S 11 Calibration curve of propenyloxypropanol

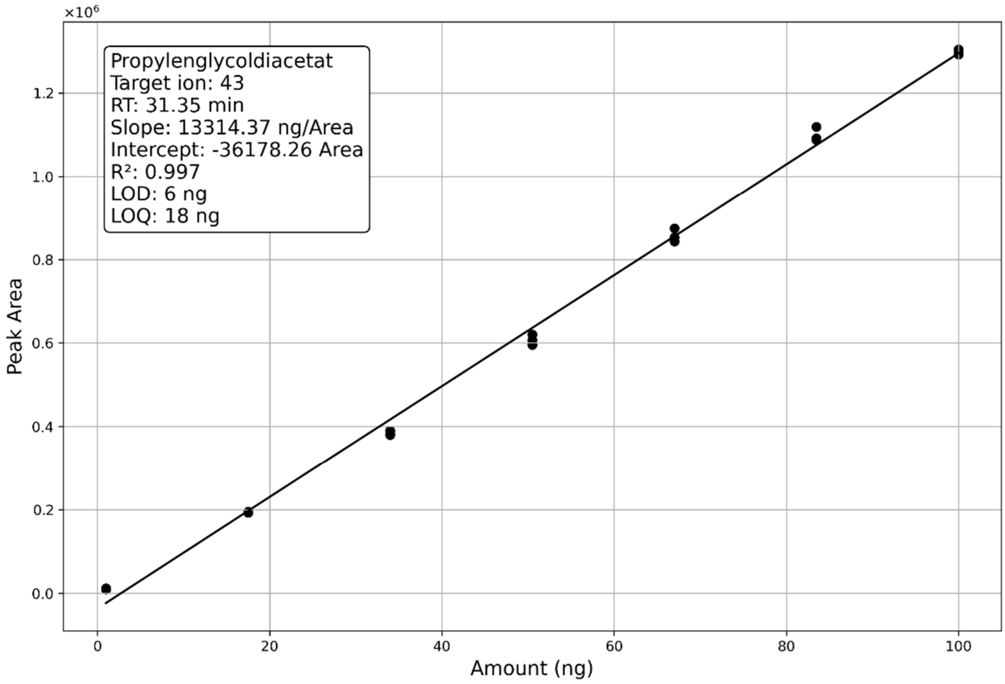

Figure S 12 Calibration curve of propyleneglycoldiacetate

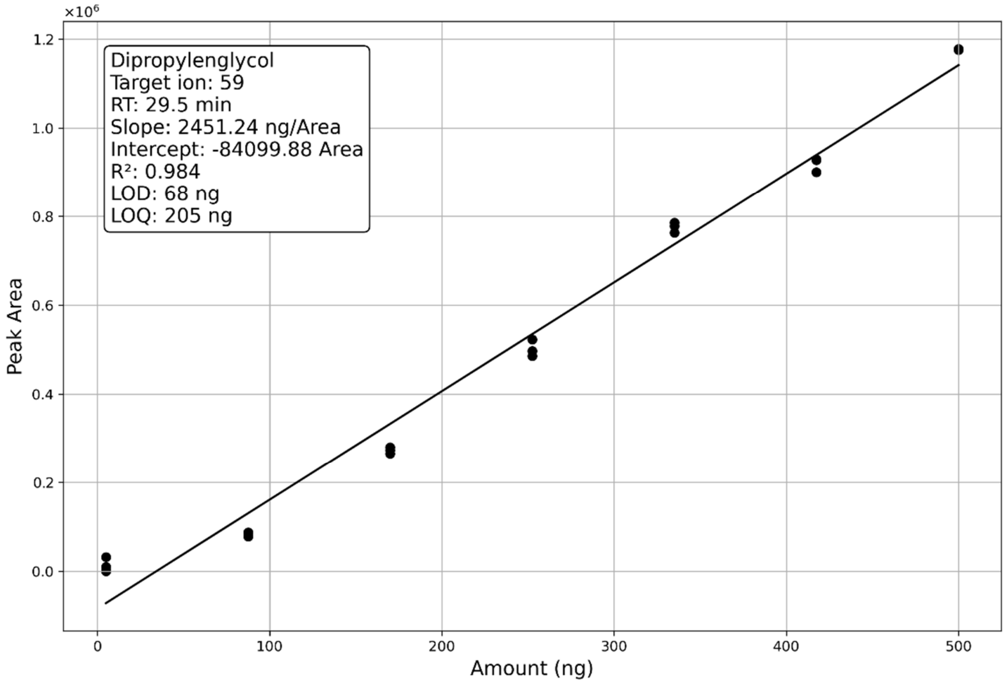

Figure S 13 Calibration curve of dipropylene glycol

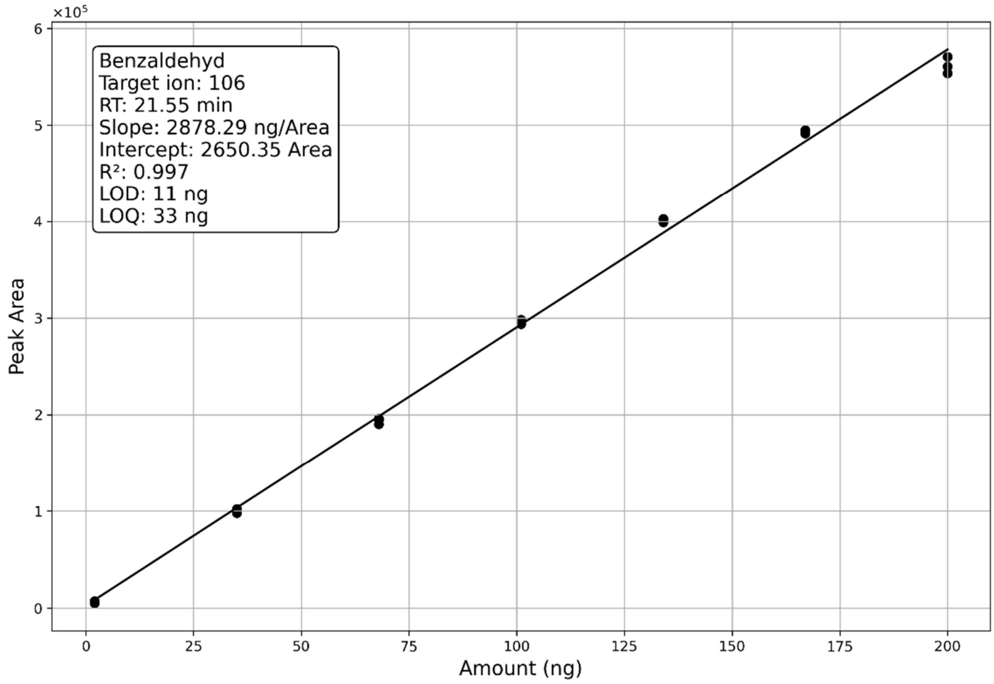

Figure S 14 Calibration curve of benzaldehyde

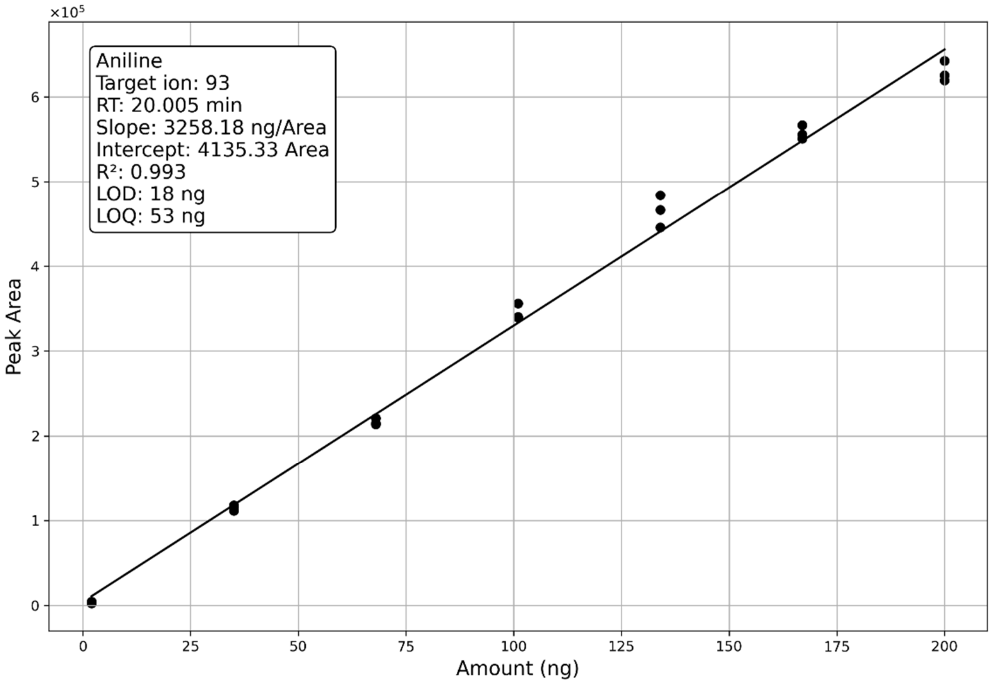

Figure S 15 Calibration curve of aniline

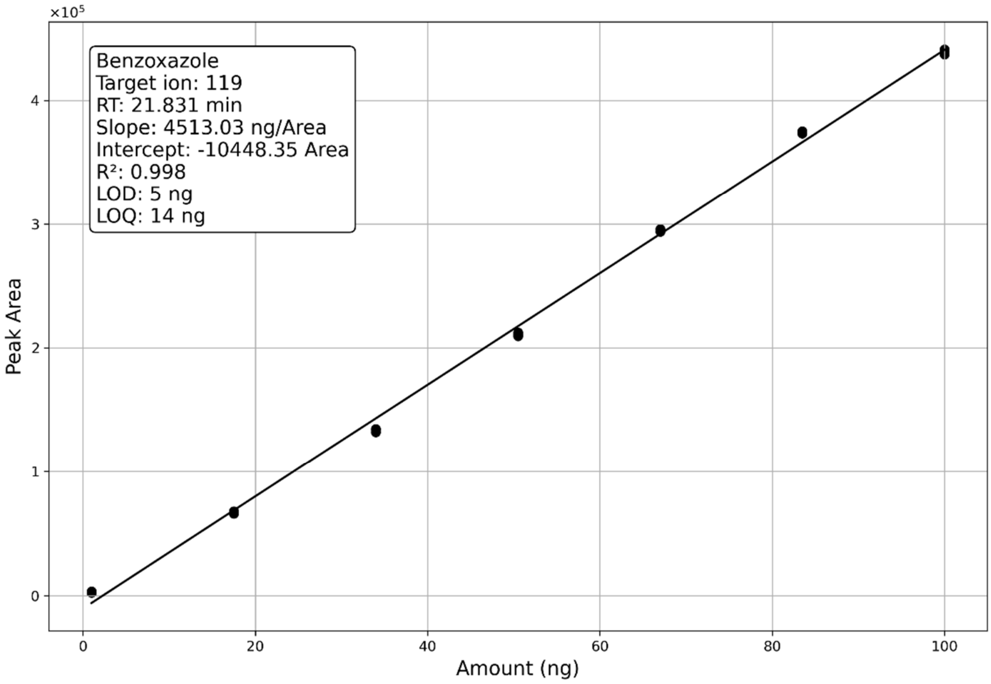

Figure S 16 Calibration curve of benzoxazole

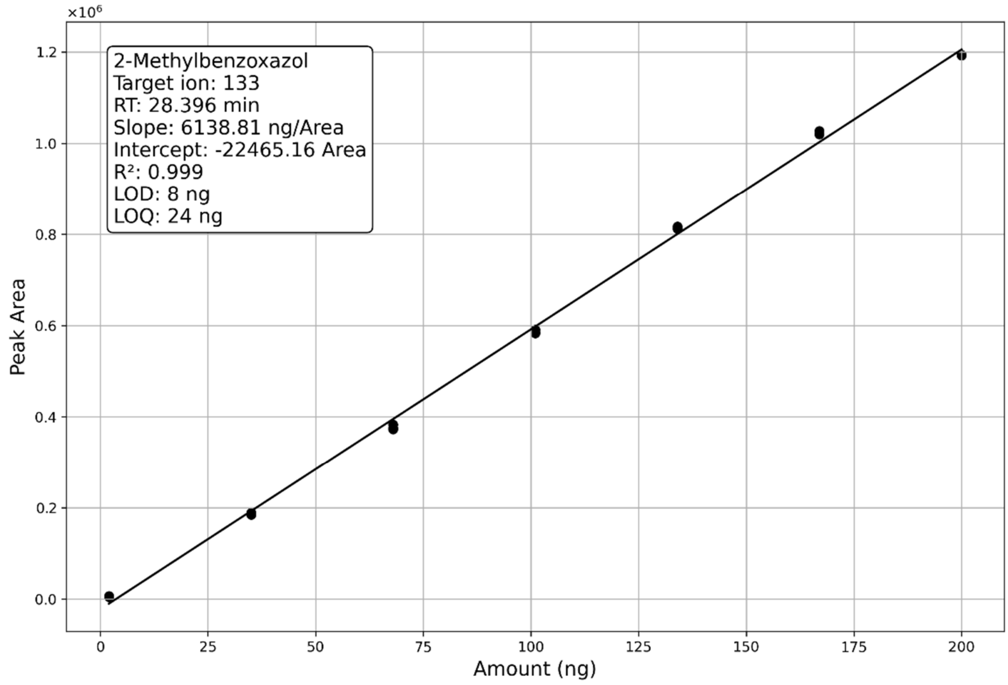

Figure S 17 Calibration curve of 2-methylbenzoxazole

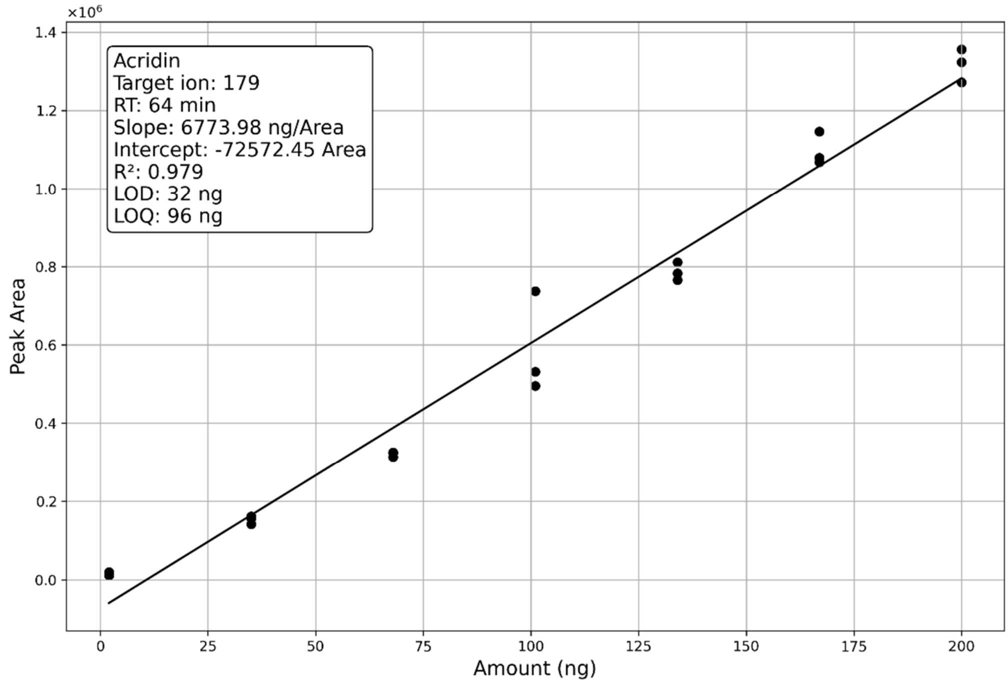

Figure S 18 Calibration curve of acridine

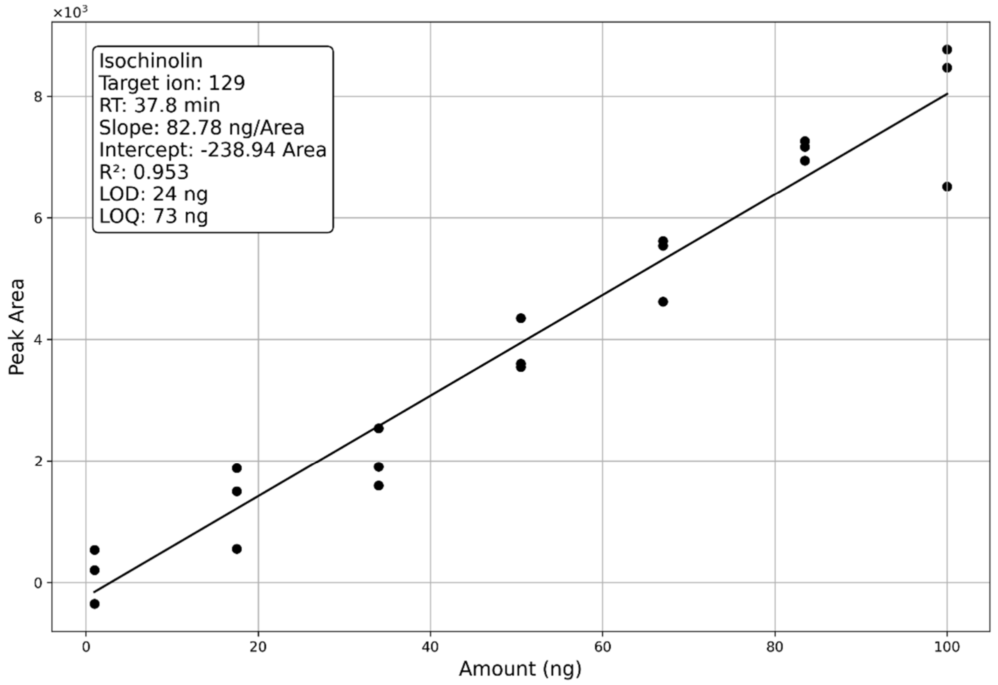

Figure S 19 Calibration curve of isoquinone

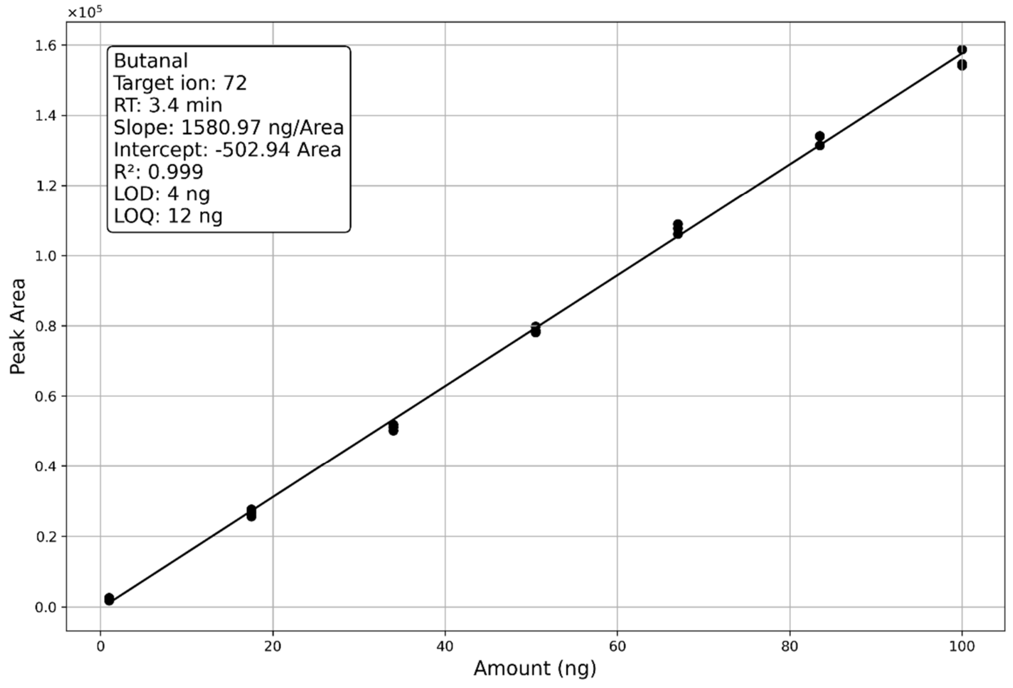

Figure S 20 Calibration curve of butyric aldehyde

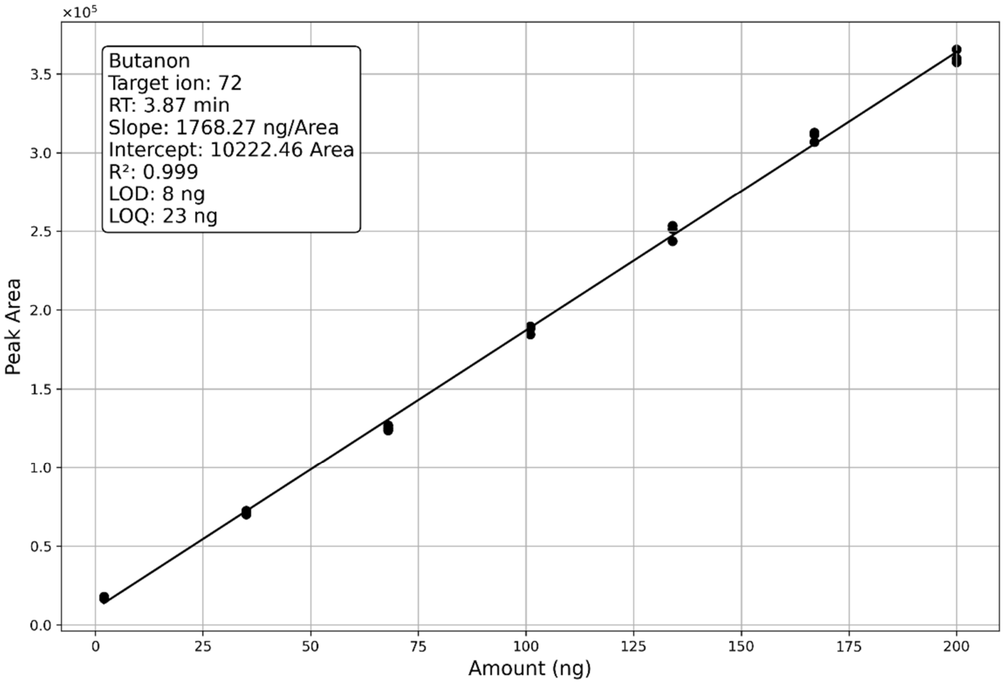

Figure S 21 Calibration curve of butanone
